# Unrepaired base excision repair intermediates in template DNA strands trigger replication fork collapse and PARP inhibitor sensitivity

**DOI:** 10.15252/embj.2022113190

**Published:** 2023-07-26

**Authors:** Almudena Serrano‐Benitez, Sophie E Wells, Lylah Drummond‐Clarke, Lilian C Russo, John Christopher Thomas, Giovanna A Leal, Mark Farrow, James Michael Edgerton, Shankar Balasubramanian, Ming Yang, Christian Frezza, Amit Gautam, Jan Brazina, Kamila Burdova, Nicolas C Hoch, Stephen P Jackson, Keith W Caldecott

**Affiliations:** ^1^ Cancer Research UK Cambridge Institute University of Cambridge Cambridge UK; ^2^ The Wellcome and Cancer Research UK Gurdon Institute and Department of Biochemistry University of Cambridge Cambridge UK; ^3^ Genome Damage and Stability Centre, School of Life Sciences University of Sussex Falmer UK; ^4^ Departament of Biochemistry, Chemistry Institute University of São Paulo São Paulo Brazil; ^5^ Yusuf Hamied Department of Chemistry University of Cambridge Cambridge UK; ^6^ CECAD Research Center, Faculty of Medicine University Hospital Cologne Cologne Germany

**Keywords:** DNA repair, DNA replication stress, DNA strand breaks, genome instability, PARP‐1, Cancer, DNA Replication, Recombination & Repair

## Abstract

DNA single‐strand breaks (SSBs) disrupt DNA replication and induce chromosome breakage. However, whether SSBs induce chromosome breakage when present behind replication forks or ahead of replication forks is unclear. To address this question, we exploited an exquisite sensitivity of SSB repair‐defective human cells lacking PARP activity or XRCC1 to the thymidine analogue 5‐chloro‐2′‐deoxyuridine (CldU). We show that incubation with CldU in these cells results in chromosome breakage, sister chromatid exchange, and cytotoxicity by a mechanism that depends on the S phase activity of uracil DNA glycosylase (UNG). Importantly, we show that CldU incorporation in one cell cycle is cytotoxic only during the following cell cycle, when it is present in template DNA. In agreement with this, while UNG induces SSBs both in nascent strands behind replication forks and in template strands ahead of replication forks, only the latter trigger fork collapse and chromosome breakage. Finally, we show that BRCA‐defective cells are hypersensitive to CldU, either alone and/or in combination with PARP inhibitor, suggesting that CldU may have clinical utility.

## Graphical abstract

Cells lacking single‐strand break repair or BRCA1/BRCA2 are hypersensitive to CldU, alone and in combination with PARP inhibitor.

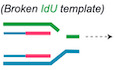

## Introduction

Cancer cells harboring mutations in genes involved in homologous recombination (HR)‐mediated DNA repair such as *BRCA1* and *BRCA2* are exquisitely sensitive to poly(ADP‐ribose) polymerase (PARP) inhibitors (Bryant *et al*, 2005; Farmer *et al*, 2005). However, the precise molecular mechanism/s of this sensitivity remain unclear (Chaudhuri & Nussenzweig, 2017; Hanzlikova & Caldecott, 2019). Several roles for PARP1 during DNA replication fork metabolism have been described that could explain the “synthetic lethal” impact of PARP inhibitors in HR‐deficient cells, such as regulating replication fork reversal (Chaudhuri *et al*, 2012; Berti *et al*, 2013), MRE11‐mediated fork resection and re‐start (Haince *et al*, 2008; Bryant *et al*, 2009), and toxic NHEJ at reversed or collapsed forks (Hochegger *et al*, 2006; Sugimura *et al*, 2008; Patel *et al*, 2011; Somyajit *et al*, 2015; Balmus *et al*, 2019).

Another likely mechanism for PARP inhibitor‐induced cytotoxicity is a delay in DNA single‐strand break repair (SSBR) imposed by loss of PARP activity and/or trapped PARP1 on SSBs, which increases SSB accumulation and their encounter with DNA replication forks, causing replication fork collapse and DNA double‐strand break (DSB) formation. The collapse of DNA replication forks and DSB formation at site‐specific SSBs has been demonstrated in both prokaryotes and higher eukaryotes (Kuzminov, 2001; Ledesma & Aguilera, 2006; Vrtis *et al*, 2021). However, the extent to which fork collapse occurs during replication of the human genome, and the source and sites of SSBs that trigger such collapse, are unclear. For example, the primary sources of PARP1 activity in proliferating cells are unligated Okazaki fragments, which are located in nascent strands behind DNA replication forks and which, in the presence of PARP inhibitors, impede nascent strand maturation (Hanzlikova *et al*, 2018; Hanzlikova & Caldecott, 2019; Vaitsiankova *et al*, 2022). Persistent SSBs and/or gaps in nascent strands could trigger replication fork collapse as described above, if they persist long enough to become located in template DNA strands during the subsequent S phase (Hanzlikova & Caldecott, 2019; Simoneau *et al*, 2021). Alternatively, nascent‐strand SSBs and/or gaps may trigger cytotoxicity during the same S phase in which they arise, when located behind replication forks and independently of fork collapse (Cong *et al*, 2021; Panzarino *et al*, 2021; Cong & Cantor, 2022). The existence of such a mechanism is supported by studies in *Escherichia coli* (Flores *et al*, 2001; Kouzminova & Kuzminov, 2012).

A major problem when comparing the impact of unrepaired SSBs present behind replication forks versus those present ahead of replication forks is the difficulty in selectively generating such breaks. Here, by incubating human cells with the thymidine analogue 5‐chloro‐2′‐deoxyuridine (CldU) for a single S phase, followed by a second S phase in the absence of CldU, we have achieved this. We show that genomic chlorouracil (ClU) rapidly induces UNG‐dependent base excision repair (BER) during S phase, which if impeded by incubation with PARP inhibitor or XRCC1 deletion results in extreme cytotoxicity. Critically, we show that while UNG‐induced SSBs and PARP activity are induced both behind and ahead of DNA replication forks, only the latter result in DNA replication fork collapse and chromosome breakage if BER is impeded. In addition, our data highlight CldU as a potent source of DNA breakage in S phase that should be employed cautiously in DNA replication experiments and reveal that BRCA1/BRCA2‐defective cells are hypersensitive to CldU alone and/or in combination with PARP inhibitor.

## Results

### Exquisite sensitivity to CldU in SSBR‐defective human cells lacking XRCC1 or PARP activity

**Figure 1:**
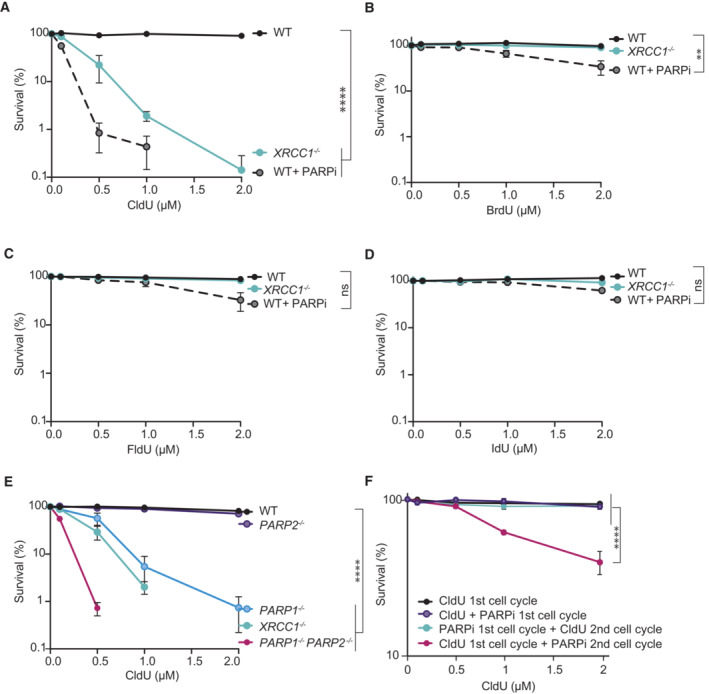
Hypersensitivity to CldU in RPE‐1 cells lacking XRCC1 or treated with PARP inhibitor A–DClonogenic survival of wild type (WT) and *XRCC1* ^−/−^ RPE‐1 cells after continuous treatment with the indicated thymidine analogues at the indicated doses. Where indicated, cells were also continuously incubated with PARP inhibitor (PARPi; 0.5 μM olaparib).EClonogenic survival of WT, *XRCC1* ^−/−^, *PARP1* ^−/−^, PARP2^−/−^, and *PARP1* ^−/−^ */PARP2* ^−/−^ RPE‐1 cells after continuous treatment with CldU at the indicated doses.FClonogenic survival of WT RPE‐1 cells after treatment with CldU at the indicated doses in the first cell cycle (first 24 h) or during the second cell cycle (following 24 h). Where indicated, cells were treated with PARP inhibitor (PARPi; 0.5 μM olaparib) during the first cell cycle (first 24 h) or the second cell cycle (following 24 h). Data information: (A–F), Error bars are the mean (±SEM) of *n* = 3 independent biological repeats. Statistical significance was assessed using Prism 9 software by two‐way ANOVA with Dunnett's multiple comparisons test. **P < 0.01, ****P < 0.0001. Source data are available online for this figure.

**Figure EV1:**
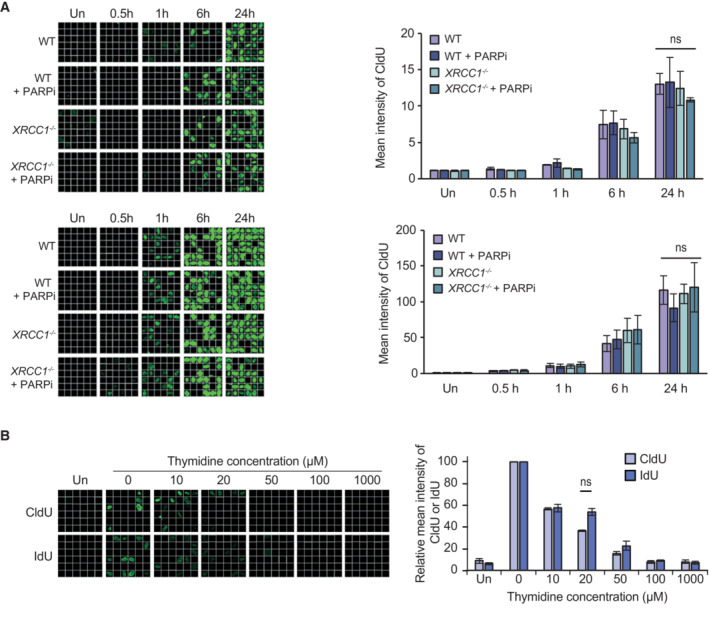
CldU and IdU are incorporated at similar rates Representative single‐cell galleries from scanR high‐content imaging (left) and quantification (right) of CldU in wild type (WT) and *XRCC1* ^−/−^ RPE‐1 cells incubated with 1 μM (top), or 10 μM (bottom) CldU for the indicated times. CldU was detected following DNA denaturation by rat anti‐BrdU (ab6326) antibody.Representative images (left) and quantification (right) as above of CldU or IdU in RPE‐1 cells incubated for 1 h with 10 μM CldU or 10 μM IdU and the indicated concentrations of thymidine. CldU was detected as above and IdU was detected following DNA denaturation by mouse anti‐BrdU (BD 347580) antibody. The width of each box (a single‐cell) in the scanR image galleries is 10 μm. Data information: (A, B) Data are the means (±SEM) of three independent biological repeats with > 1,000 cells (technical replicates) scored per sample per experiment, by scanR software. Statistical significance was assessed by (A) one‐way ANOVA with Tukey's multiple comparisons test or (B) two‐tailed paired *T*‐test. ns, not significant; *P* > 0.05.

The single‐strand break repair (SSBR) protein XRCC1 is critical for survival of CHO (Chinese hamster ovary) cells in media containing the thymidine analogue CldU (Dillehay *et al*, 1984). To examine if this is also the case in human cells, we compared the impacts of CldU, and the related thymidine analogues bromodeoxyuridine (BrdU), fluorodeoxyuridine (FldU), and iododeoxyuridine (IdU), on the survival of wild‐type and *XRCC1* ^−/−^ RPE‐1 cells. Indeed, human *XRCC1* ^−/−^ RPE‐1 cells were extremely sensitive to CldU (Fig 1A), with < 1% of cells surviving continuous incubation with 2 μM of this nucleoside; a concentration that was largely non‐toxic in wild‐type RPE‐1 cells. In contrast, BrdU, FldU, and IdU exerted little or no cytotoxicity over the concentrations employed, in either wild‐type or *XRCC1* ^−/−^ RPE‐1 cells (Fig 1B–D). The exquisite sensitivity of *XRCC1* ^−/−^ cells to CldU was not a result of elevated CldU uptake or incorporation, which was similar in wild type and *XRCC1* ^−/−^ cells (Fig EV1A). Nor did the different analogues differ in their uptake or incorporation, as suggested by the similar efficiency with which CldU and IdU incorporation was suppressed in RPE‐1 cells by exogenous thymidine competitor (Fig EV1B). Indeed, CldU and IdU incorporation were both suppressed by ~50% by an equimolar concentration of exogenous thymidine (10 μM), arguing that the three nucleotides are incorporated in RPE‐1 cells with similar efficiencies.

**Figure EV2:**
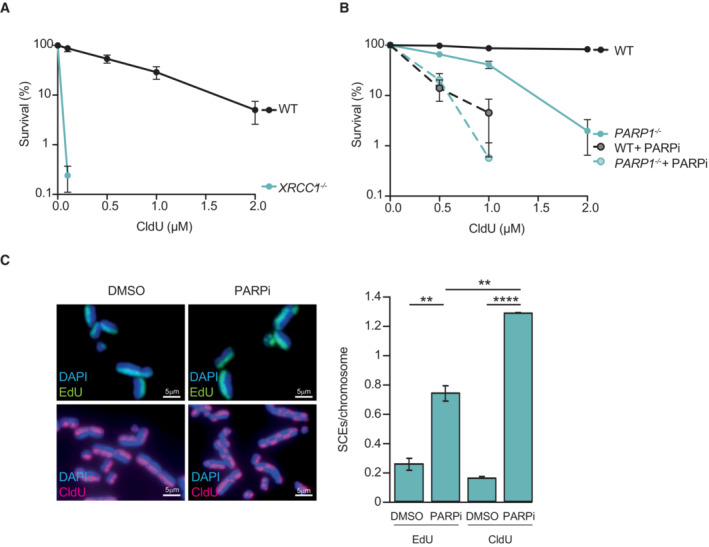
Hypersensitivity to CldU in U2OS cells lacking XRCC1 or treated with PARP inhibitor Clonogenic survival of wild type (WT) and *XRCC1* ^−/−^ U2OS cells following continuous treatment with the indicated concentrations of CldU.Clonogenic survival of WT and *PARP1* ^−/−^ U2OS cells following continuous treatment with the indicated concentrations of CldU in the absence or presence of 0.5 μM PARPi, as indicated.Representative images (left) and quantification (right) of sister chromatid exchanges detected by EdU click chemistry or anti‐CldU immunofluorescence in U2OS cells treated with either EdU or CldU for the first 24 h and then with DMSO or 0.5 μM Ku58948 (PARPi) for the subsequent 24 h. Scale bars are 5 μm. Data information: (A, B) Data are the mean of three independent biological repeats (±SEM). (C) Data are the means (±SEM) of two independent biological repeats, with 500 chromosomes (technical replicates) scored per sample per experiment. Statistical significance was determined by one‐way ANOVA with Tukey's multiple comparisons test. **P < 0.005; ****P < 0.0001.

To examine whether the hypersensitivity of *XRCC1* ^−/−^ cells to CldU is a feature of cells with other defects in SSBR, we employed the PARP inhibitor (PARPi) olaparib. Indeed, incubation of wild‐type RPE‐1 cells with olaparib exerted a similar, if not greater, impact on CldU sensitivity than did loss of XRCC1, while once again showing little impact in the presence of BrdU, FldU, or IdU (Fig 1A–D). The impact of olaparib did not depend on PARP trapping, because *PARP1* ^−/−^ RPE‐1 cells were also hypersensitive to CldU, and additional deletion of PARP2 increased this sensitivity to a level similar to that observed in wild‐type cells incubated with olaparib, which inhibits both PARP1 and PARP2 (Fig 1E). Collectively, these data demonstrate that in human RPE‐1 cells both PARP activity and XRCC1 are important for cell survival in the presence of CldU. This phenotype was not specific to RPE‐1 cells, because similar results were observed with human U2OS cells (Fig EV2A and B).

We reasoned that the cytotoxicity of CldU in SSBR‐defective cells likely reflected the impact of unrepaired SSBs on DNA replication. To better understand how CldU induced cytotoxicity, we compared the impact of adding PARPi for 24 h during CldU treatment with adding PARPi for 24 h after CldU treatment, to inhibit SSBR during the first or second S phase following CldU addition, respectively. Strikingly, only in RPE‐1 cells in which PARPi was present after CldU treatment was CldU cytotoxic (Fig 1F). This result suggests that CldU was toxic only if SSBR was inhibited during the S phase that followed chlorouracil (ClU) incorporation, when unrepaired SSBs were present in template DNA strands.

### SSBR defects trigger DSBs and SCEs during the replication of template DNA containing chlorouracil

**Figure 2:**
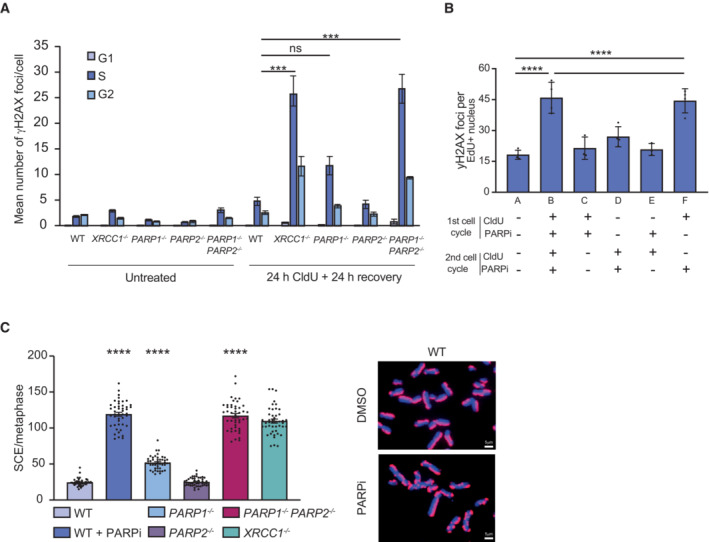
PARP inhibition triggers sister chromatid breakage and exchange during the replication of CldU‐substituted template DNA AQuantification from scanR high‐content imaging of H2AX‐pSer139 (γH2AX) foci in wild type (WT), *XRCC1* ^−/−^, *PARP1* ^−/−^, *PARP2* ^−/−^, and *PARP1* ^−/−^ */PARP2* ^−/−^ RPE‐1 cells prior to (untreated) and after 24 h incubation with 1 μM CldU followed by a further 24 h in the absence of 1 μM CldU. Cells were pulse labeled with EdU for the final 30 min and both EdU and DNA content (DAPI staining) used to determine cell cycle stage.BQuantification of yH2AX foci in RPE‐1 cells treated with 1 μM CldU and/or 0.5 μM olaparib (PARPi) for an initial 24 h (1st cell cycle) and/or a subsequent 24 h (2nd cell cycle), as indicated. yH2AX foci were scored in S phase cells as described above.CRepresentative images (right) and quantification (left) of sister chromatid exchanges detected by anti‐CldU immunofluorescence in WT, *PARP1* ^−/−^, *PARP2* ^−/−^, *PARP1* ^−/−^ */PARP2* ^−/−^ and *XRCC1* ^−/−^ RPE‐1 cells treated with 1 μM CldU for 24 h followed by DMSO vehicle or 0.5 μM olaparib (PARPi) for a subsequent 24 h, as indicated. Scale bars are 1 μm. Data information: (A, B) Data are the means (±SEM) of 3 (A) or 4 (B) independent biological repeats with > 1,000 cells (technical replicates) scored by scanR software per sample per experiment. (C), data are the means (±SD) of three independent biological repeats, with 15 metaphases (technical replicates) scored per sample per experiment. Statistical significance was assessed using Prism 9 software by one‐way ANOVA with Tukey's multiple comparisons test (***P < 0.001; ****P < 0.0001). Source data are available online for this figure.

The presence of unrepaired SSBs in template DNA strands can result in replication fork collapse and the formation of DSBs (Kuzminov, 2001). To examine whether CldU induced DSBs, we measured the number of γH2AX foci (Rogakou *et al*, 1998, 1999) in RPE‐1 cells incubated with CldU for 24 h followed by a further 24 h without. Indeed, whereas the number of γH2AX foci were increased ~2‐fold in wild‐type S phase RPE‐1 cells, they were increased ~10‐fold in S phase RPE‐1 cells lacking either XRCC1 or both PARP1 and PARP2 (Fig 2A). γH2AX foci were also increased in S phase in RPE‐1 cells that were incubated continuously with PARPi (Fig 2B, *bar graph b*). Importantly, γH2AX foci were also increased if PARPi was present during the 24 h following ClU incorporation (Fig 2B, *bar graph f*), but not if PARPi was only present before (Fig 2B, *bar graph e*) or during (Fig 2B, *bar graphs c and d*) ClU incorporation. These data suggest that treatment with PARPi during the S phase that follows ClU incorporation, when this thymine analogue was present in template DNA strands, was necessary for DSB induction.

DSBs arising from the replication of template DNA strands containing SSBs can trigger increased sister chromatid exchanges (SCEs), as a result of the employment of homologous recombination‐mediated mechanisms for DSB repair (Dillehay *et al*, 1983; El‐Khamisy *et al*, 2005; Hoch *et al*, 2017). Indeed, the frequency of SCEs detected following the replication of ClU‐substituted template DNA was increased ~5‐fold in RPE‐1 cells lacking either XRCC1 or both PARP1 and PARP2, when compared to wild‐type RPE‐1 cells (Fig 2C). Similarly, incubation with PARPi during the 24 h that followed ClU incorporation triggered similar levels of SCEs, consistent with the induction of these recombination events during the replication of ClU‐substituted template DNA (Figs 2C and EV2C).

### CRISPR screens for regulators of olaparib‐induced CldU hypersensitivity

**Figure 3:**
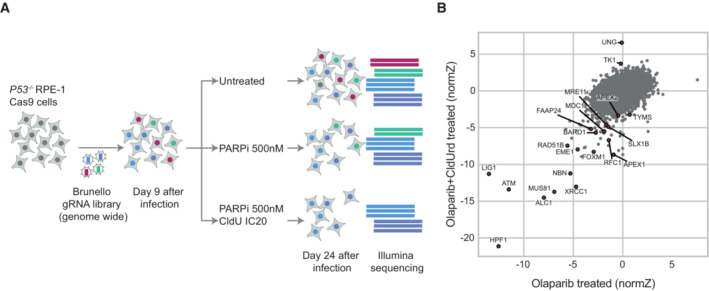
CRISPR screening to identify human genes affecting sensitivity to CldU and/or olaparib Schematic of the screen pipeline. Two independent clones of P53‐deficient RPE‐1 cells expressing Cas9 nuclease were infected with the Brunello library containing gRNAs targeting the whole genome (coverage 500×). After selection for infected cells and population sampling 9 days after infection, the starting cell populations were divided into three groups. One group was an untreated control, a second group was treated continuously with a sublethal concentration of olaparib (500 nM), and a third population was continuously treated with both 500 nM olaparib and 0.2 μM CldU; a combination that kills ~20% of cells (IC20). Infected cell populations were sampled every 3 days from days 9 to 24, genomic DNA isolated, and integrated gRNA abundances measured relative to those in the “pre‐treated” cell population at day 9 by deep sequencing. Results were analyzed by the Drug Z algorithm, a positive NormZ score stands for an enrichment of the guide targeting a specific gene in the treated condition vs non‐treated control. A negative NormZ score stands for a decrease in the abundance of the guide RNA in the treated condition vs. non‐treated control.Screen data. NormZ scores from cells treated only with olaparib are plotted on the X axis and those from cells treated with both olaparib and CldU are plotted on the Y axis. DNA damage response—related genes are highlighted. Source data are available online for this figure.

The requirement for both PARP1 and XRCC1 for cell proliferation in the presence of template DNA containing ClU suggested that when incorporated into genomic DNA, significant amounts of this nucleobase are converted into SSBs. To identify the mechanism by which this occurs, we conducted genome‐wide CRISPR screens to identify genes that affect PARPi‐induced CldU hypersensitivity (Fig 3A). For this, we employed an RPE‐1 cell line constitutively expressing Cas9 and deficient for p53 (Cas9‐*TP53* ^−/−^ RPE‐1 cells), as p53 deficiency enables better identification of some factors whose loss affects cell viability (Haapaniemi *et al*, 2018; Bowden *et al*, 2020; Awwad *et al*, 2023). Cas9‐*TP53* ^−/−^ RPE‐1 cells were transduced with the Brunello genome‐wide CRISPR knockout library and then either left untreated or treated chronically with 0.5 μM olaparib in the absence or presence of 0.2 μM CldU. These concentrations of PARPi and CldU were non‐toxic when applied alone but killed ~20% of Cas9‐*TP53* ^−/−^ RPE‐1 cells when applied together in the screening format.

**Figure EV3:**
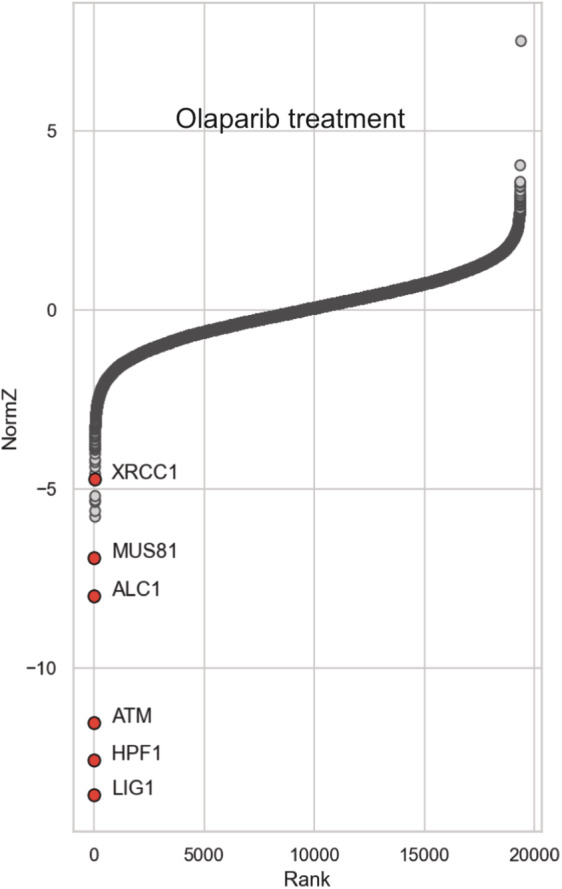
CRISPR screen identifying human genes affecting sensitivity to olaparib alone Rank plot representation of CRISPR‐Cas9 screen data. Results from cells treated with 0.5 μM olaparib alone are plotted and PARP1‐related hits are highlighted.

These synthetic enrichment/dropout screens identified various genes whose inactivation was predicted to decrease or increase PARPi‐induced sensitivity to CldU (Fig 3B). As expected, genes whose gRNAs decreased in relative abundance in cells treated with olaparib alone included those encoding LIG1, HPF1, ATM, ALC1 (CHDL1), MUS81, and XRCC1 (Fig EV3), consistent with their established roles in protecting cells from killing by PARP inhibitors (Zimmermann *et al*, 2018; DeWeirdt *et al*, 2020). Notably, the gRNAs for these genes were also those that were most depleted by co‐treatment of olaparib and CldU (Fig 3B). In contrast, the gRNAs that were most enriched when cells were treated with both CldU and olaparib were those targeting UNG (uracil DNA glycosylase) or TK1 (thymidine kinase; Fig 3B). Strikingly, these guides were not enriched by treatment with olaparib alone, indicating that UNG and TK activity specifically invoke cellular sensitivity to the combination of these drugs. TK1 phosphorylates thymidine and its analogues to generate nucleoside monophosphates, and may thus promote CldU sensitivity by increasing the level of CldUTP, and thus of chlorouracil incorporation into DNA (Bitter *et al*, 2020). In contrast, UNG is a DNA glycosylase that excises uracil from DNA during DNA base excision repair (Krokan *et al*, 2001).

### CldU hypersensitivity is triggered by S phase UNG activity on template DNA strands

**Figure 4:**
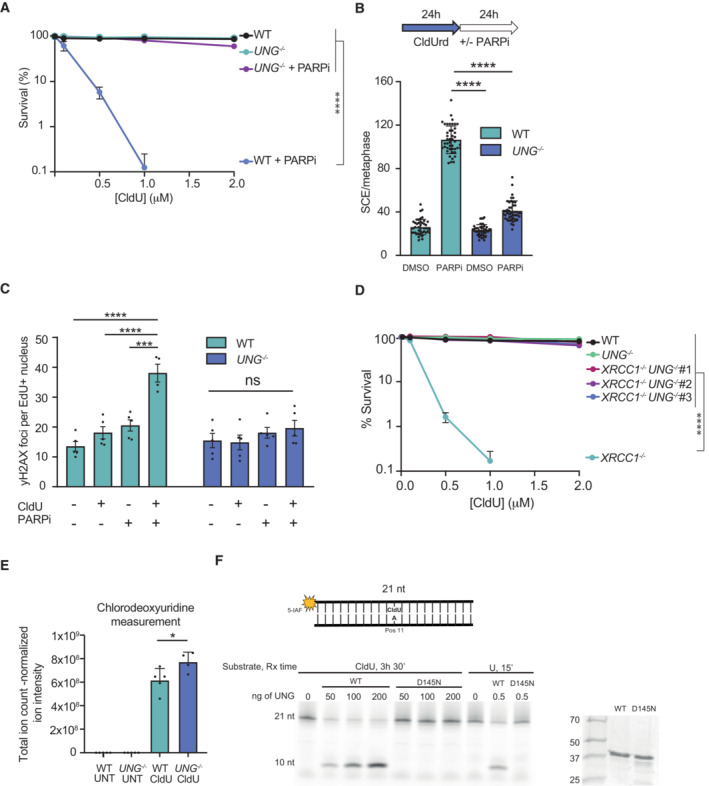
UNG deletion suppresses CldU‐induced DNA replication stress in SSBR‐deficient cells Clonogenic survival of the indicated cell lines treated continuously with 0.5 μM olaparib and the indicated concentrations of CldU for 10 days.Quantification of sister chromatid exchanges (SCEs) detected by anti‐CldU immunofluorescence in WT and *UNG* ^−/−^ RPE‐1 cells. Cells were treated with 1 μM CldU for 24 h and then with either DMSO vehicle or 0.5 μM olaparib (PARPi) for the subsequent 24 h.Quantification of yH2AX foci in WT and *UNG* ^−/−^ RPE‐1 cells treated with/without 1 μM CldU for 24 h and with/without 0.5 μM olaparib (PARPi) for 24 h, as indicated. yH2AX foci were scored in S phase cells, which were identified by EdU pulse labeling for the final 30 min.Clonogenic survival of WT, *UNG* ^−/−^, *XRCC1* ^−/−^, and three independent *XRCC1* ^−/−^ */UNG* ^−/−^ RPE‐1 clones (#6, #20, #27, labelled 1‐3 for simplicity) after continuous treatment with the indicated concentrations of CldU for 10 days.CldU levels in genomic DNA extracted from WT or *UNG* ^−/−^ RPE‐1 cells following treatment 1 μM CldU for 30 h. Genomic DNA was digested with Nucleoside Digestion Mix (NEB) and nucleoside composition quantified by LC–MS. CldU levels are shown.A 5‐Iodoacetamidofluorescein (5‐IAF) labeled 21‐mer oligodeoxyribonucleotide with ClU or U at position 11, as indicated, was incubated with the indicated amounts of purified wild‐type human histidine‐tagged UNG or histidine‐tagged catalytic mutant UNG^D145N^. DNA products were resolved by denaturing PAGE (left). Aliquots of recombinant UNG and UNG^D145N^ were resolved by SDS–PAGE and stained with Coomassie blue (right). Data information: (A, D) Data are the means (±SEM) of three independent biological repeats and statistical significance was assessed by two‐way ANOVA with Dunnett's multiple comparisons test. (B, C) Data are the means (±SD) of 3 (B) or 5 (C) independent biological repeats, with 15 metaphases (B) or > 1,000 cells (C) scored as technical replicates per sample per experiment. Statistical significance was determined by one‐way ANOVA with Tukey's multiple comparisons test. (E) Data are the means (±SD) of at least four dishes (technical replicates), with each replicate dish treated and processed independently. Statistical significance was determined by two‐tailed unpaired *t*‐test. *P < 0.05; **P < 0.001; ****P < 0.0001. Source data are available online for this figure.

**Figure EV4:**
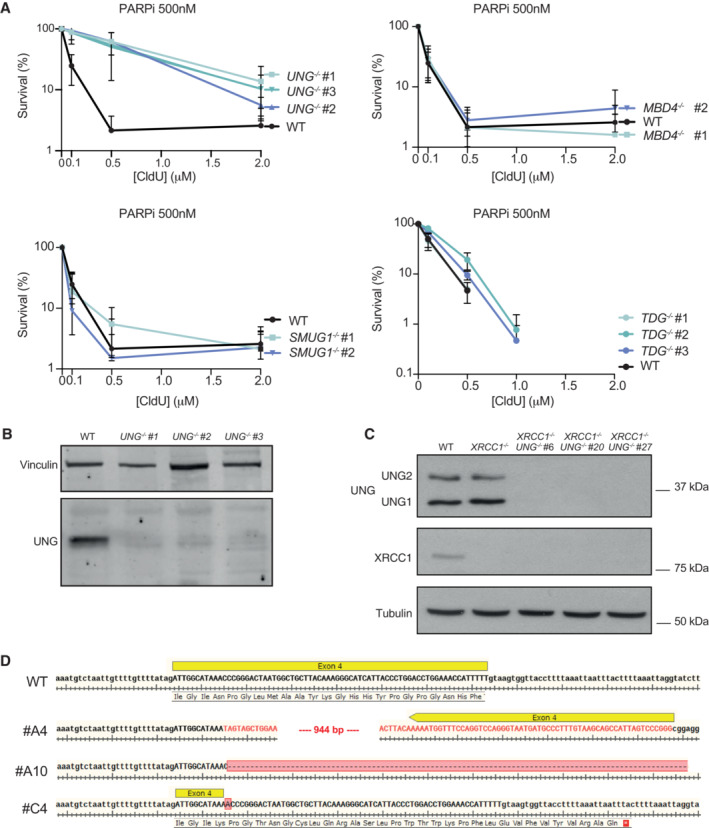
Suppression of CldU sensitivity is specific to loss of UNG Clonogenic survival of RPE‐1 cell lines deleted of the indicted DNA glycosylases following continuous treatment with 0.5 μM olaparib and the indicated concentrations of CldU. Data are the means (±SD) of three independent biological repeats.Immunoblotting verification of UNG protein levels in *UNG* ^−/−^ RPE‐1 cell lines generated by CRISPR‐Cas9 gene editing.Immunoblotting verification of UNG and XRCC1 protein levels in WT, *XRCC1* ^−/−^, and *XRCC1* ^−/−^/*UNG* ^−/−^ (clones #6, #20, #27) RPE‐1 cell lines.PCR amplification of genomic DNA surrounding the gRNA target sites followed by Sanger sequencing to confirm TDG gene editing in three individual RPE‐1 clones (#A4, #A10, #C4). Note that these clones are labeled 1–3, in panel (A) above. Lower case letters represent introns and upper case represent exons. DNA base substitutions, insertions, and deletions are highlighted in red.

To further assess the impact of UNG loss on CldU sensitivity, we generated *UNG* ^−/−^ RPE‐1 cells (Fig EV4A and B). Strikingly, whereas wild‐type RPE‐1 cells were exquisitely sensitive to CldU in the presence of olaparib, *UNG* ^−/−^ cells were not (Fig 4A). In contrast, deletion of any of three other human uracil glycosylases (SMUG1, TDG and MBD4) failed to noticeably affect CldU sensitivity (Fig EV4A and D). Importantly, UNG deletion also prevented the increased frequency of SCEs (Fig 4B) and induction of γH2AX foci (Fig 4C) following incubation of cells with CldU and olaparib, and also ablated the hypersensitivity of *XRCC1* ^−/−^ RPE‐1 cells to CldU (Figs 4D and EV4C). The simplest explanation for these data is that UNG activity excises genomic ClU, leading to SSB intermediates of BER that require PARP1 and XRCC1 for their rapid repair. Consistent with this idea, mass spectrometry revealed that CldU‐treated *UNG* ^−/−^ RPE‐1 cells contained ~30% more genomic ClU than did wild‐type RPE‐1 cells (Fig 4E). Moreover, recombinant wild‐type human UNG excised ClU from a short synthetic oligonucleotide, albeit with an efficiency > 100‐fold less than its canonical substrate, uracil (Fig 4F). In contrast, recombinant human UNG harboring a catalytic mutation that reduces UNG activity ~20‐fold (Mol *et al*, 1995) was unable to excise ClU (Fig 4F).

**Figure 5:**
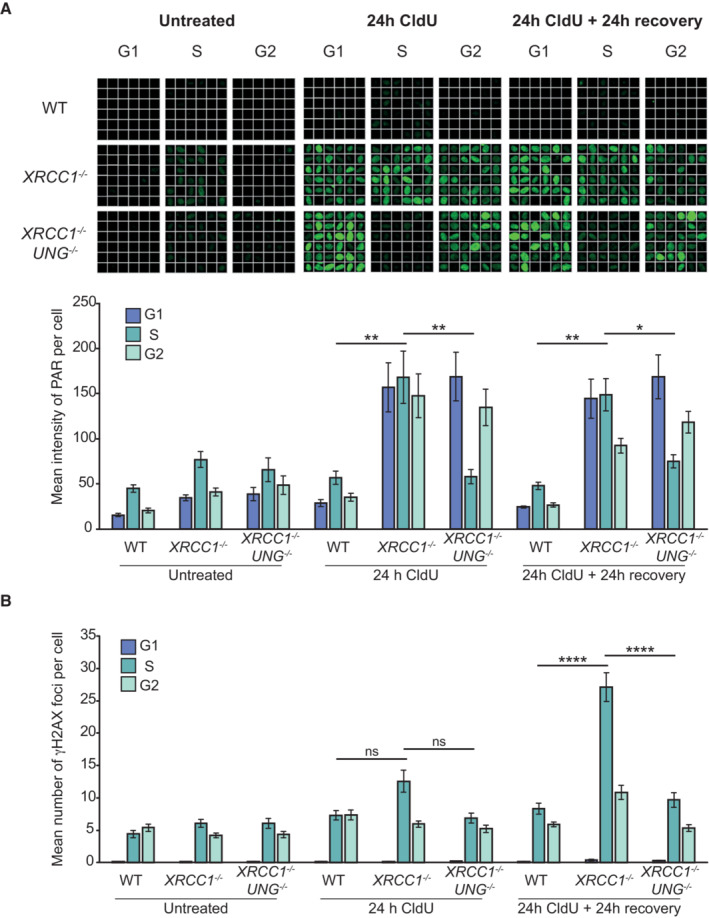
UNG deletion suppresses CldU‐induced SSBs and DSBs in SSBR‐deficient cells Representative single‐cell galleries from scanR high content imaging (top) and quantification (bottom) of poly(ADP‐ribose) in WT, *XRCC1* ^−/−^, and *XRCC1* ^−/−^ */UNG* ^−/−^ RPE‐1 cells in the absence of CldU treatment (“untreated”), after treatment with 10 μM CldU for 24 h (“24 h CldU”), and after treatment with CldU for 24 h followed by a further 24 h in the absence of CldU (“24 h CldU + 24 h recovery”). Cells were co‐incubated for the final 30 min with EdU to label S‐phase cells and with PARG inhibitor (PARGi) to prevent poly(ADP‐ribose) degradation. G1/G2 cells were distinguished based on their DAPI content. The width of each box (single‐cell) in the scanR image galleries is 10 μm.Quantification of γH2AX foci in WT, *XRCC1* ^−/−^, and *XRCC1* ^−/−^ */UNG* ^−/−^ RPE‐1 cells treated as in panel (A). Data information: Data are the mean (±SEM) of four independent biological repeats with > 1,000 cells (technical repeats) scored per sample per experiment. Statistical significance was assessed by one‐way ANOVA with Tukey's multiple comparisons test. *P < 0.05, **P < 0.01, ****P < 0.0001. Source data are available online for this figure.

**Figure EV5:**
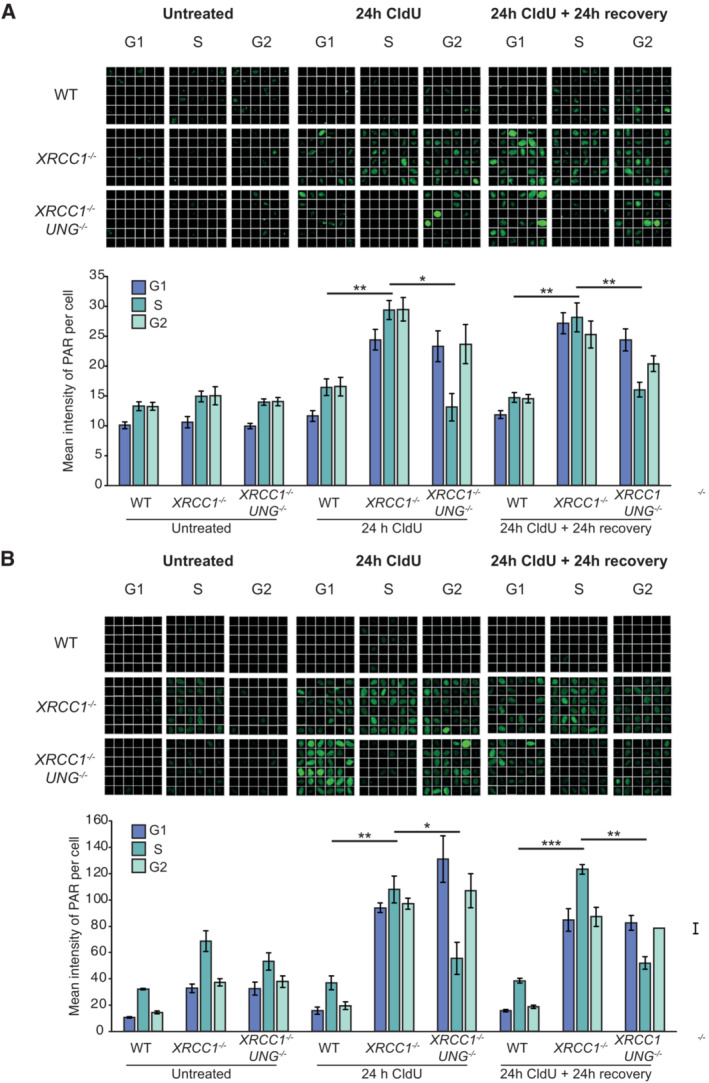
UNG deletion suppresses CldU‐induced SSB formation in SSBR‐deficient cells Representative single‐cell galleries from scanR high‐content imaging (top) and quantification (bottom) of poly(ADP‐ribose) in WT, *XRCC1* ^−/−^ or *XRCC1* ^−/−^ *UNG* ^−/−^ RPE‐1 cells following treatment with 10 μM CldU for 24 h followed by 24 h recovery where indicated. Cells were co‐incubated for the final 30 min with EdU to label cells in S‐phase, but PARGi was omitted. Note that ADP‐ribosylation was detected in *XRCC1* ^−/−^ cells even in the absence of PARGi.Representative images (top) and quantification (bottom) as above of poly(ADP‐ribose) in WT, *XRCC1* ^−/−^, and *XRCC1* ^−/−^ *UNG* ^−/−^ RPE‐1 cells following treatment with 1 μM CldU for 24 h followed by 24 h recovery where indicated. Cells were co‐incubated for the final 30 min with EdU to label cells in S‐phase and PARG inhibitor (PARGi) to prevent poly(ADP‐ribose) degradation. The width of each box (single‐cell) in the scanR image galleries is 10 μm. Data information: (A, B) Data are the mean (±SEM) of 4 (A) or 3 (B) independent biological repeats with > 1,000 cells (technical replicates) scored per sample per experiment by scanR software. Statistical significance was assessed by one‐way ANOVA with Tukey's multiple comparisons test. *P < 0.05, **P < 0.01, ***P < 0.001.

**Figure EV6:**
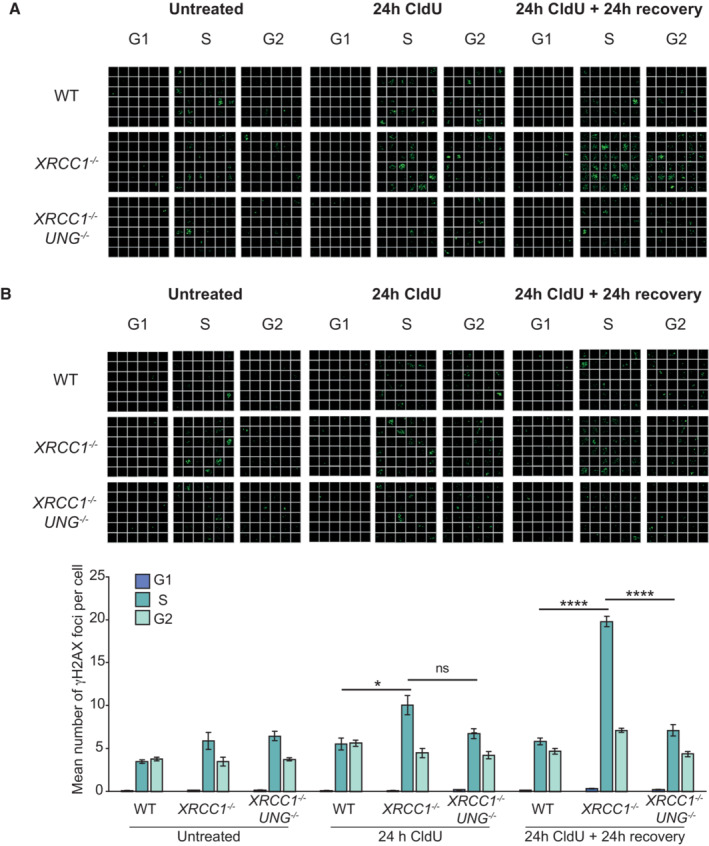
UNG deletion suppresses CldU‐induced DSB formation in SSBR‐deficient cells Representative single‐cell galleries from scanR high‐content imaging of H2AX‐pSer139 (γH2AX) foci in WT, *XRCC1* ^−/−^ or *XRCC1* ^−/−^ *UNG* ^−/−^ RPE‐1 cells following treatment with 10 μM CldU for 24 h followed by 24 h recovery where indicated. Cells were co‐incubated for the final 30 min with EdU to label cells in S‐phase.Representative images (top) and quantification (bottom) as above of H2AX‐pSer139 (γH2AX) foci in WT, *XRCC1* ^−/−^ or *XRCC1* ^−/−^ *UNG* ^−/−^ RPE‐1 cells following treatment with 1 μM CldU for 24 h followed by 24 h recovery, where indicated. Cells were co‐incubated for the final 30 min with EdU to label cells in S‐phase. Data information: Data are the means of four independent biological repeats (±SEM) with > 1,000 cells (technical replicates) scored per sample per experiment by scanR software. Statistical significance was assessed by one‐way ANOVA with Tukey's multiple comparisons test. *P < 0.05, ****P < 0.0001. The width of each box (a single‐cell) in the scanR image galleries is 10 μm.

Our data indicating that CldU cytotoxicity was restricted to the cell cycle that followed ClU incorporation suggested that only SSBs present in template DNA strands are cytotoxic. Alternatively, it could indicate that the induction of SSBs by UNG‐dependent BER only occurs in template DNA strands. To examine these possibilities, we quantified the level of CldU‐induced ADP‐ribosylation throughout the two cell cycles that followed addition of CldU, as an indirect measure of SSB levels. As expected, we detected little CldU‐induced ADP‐ribosylation in wild‐type RPE‐1 cells at any time point, consistent with the SSBR proficiency of these cells (Fig 5A). In contrast, we detected high levels of CldU‐induced ADP‐ribosylation at all time points in *XRCC1* ^−/−^ cells (Fig 5A), consistent with their SSBR defect during BER. Indeed, CldU‐induced ADP‐ribosylation was sufficiently high in *XRCC1* ^−/−^ cells to be detected even in the absence of poly(ADP‐ribose) glycohydrolase (PARG) inhibitor (Fig EV5A). More importantly, the level of ADP‐ribosylation in *XRCC1* ^−/−^ cells was similar in the first and second cell cycles following CldU addition, ruling out differences in SSB induction as an explanation for why CldU cytotoxicity was restricted to the second cell cycle (Figs 5A and EV5A). This was true even if we employed 10‐fold lower concentrations of CldU (1 μM), to better match the conditions employed in our toxicity experiments (Fig EV5B). Notably, UNG loss ablated CldU‐induced PARP activity only in those *XRCC1* ^−/−^ cells in S phase (Fig 5A), consistent with the S phase specificity and localization of UNG within the replisome (Otterlei *et al*, 1999; Hardeland *et al*, 2007). More importantly, UNG deletion ablated CldU‐induced PARP activity both in the S phase in which CldU was incorporated (“24 h CldU”) and in the S phase that followed (“24 h CldU + 24 h recovery”), suggesting that UNG is active both behind and ahead of DNA replication forks. Collectively, these data suggest that the UNG‐induced SSBs that are cytotoxic are those present specifically in the S phase that follows CldU incorporation, most likely because of their presence in template DNA strands ahead of approaching DNA replication forks. Indeed, consistent with this idea, whereas the level of UNG‐induced ADP‐ribosylation in *XRCC1* ^−/−^ cells was similar in both S phases, the level of UNG‐induced DSBs was highest in the S phase that followed CldU incorporation (Figs 5B, and EV6A and B).

### UNG‐induced BER intermediates in template DNA strands trigger DNA replication fork collapse

**Figure 6:**
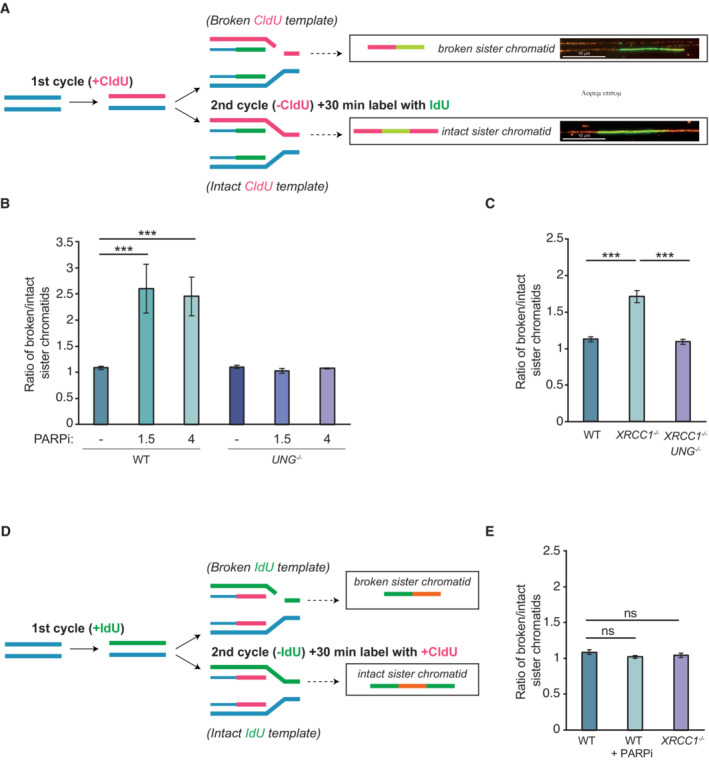
UNG triggers DNA replication fork collapse and sister chromatid breakage on CldU‐containing template DNA strands Schematic depicting experimental design. Cells were incubated with CldU for one cell cycle (24 h), washed and incubated in label‐free media for 1 h, and then pulse‐labeled with IdU for 0.5 h during the second cell cycle. Following DNA combing and immunodetection of CldU (red) and IdU (green), broken sister chromatids were detected as tracts of red (CldU) adjacent to tracts of yellow (IdU/CldU), and intact sister chromatids were detected as tracts of yellow (IdU/CldU) flanked by tracts of red (CldU). Scale bars are 10 μm.Ratio of broken/intact sister chromatids in WT and *UNG* ^−/−^ RPE‐1 cells following labeling as described in panel (A) and incubated or not with 20 μM olaparib (PARPi) for 1.5 or 4 h prior to and including the IdU pulse‐label.Ratio of broken/intact sister chromatids in WT, *XRCC1* ^−/−^, and *XRCC1* ^−/−^ */UNG* ^−/−^ RPE‐1 cells following labeling as described in panel (A).Schematic depicting an experimental design as in panel (A) but in which the order of DNA labeling is reversed. Cells were incubated with IdU for one cell cycle (24 h), washed and incubated in label‐free media for 1 h, and then pulse‐labeled with CldU for 0.5 h during the second cell cycle. Following DNA combing and immunodetection of IdU (red) and CldU (green), broken sister chromatids were detected as tracts of red (ldU) adjacent to tracts of yellow (IdU/CldU), and intact sister chromatids were detected as tracts of yellow (IdU/CldU) flanked by tracts of red (ldU).Ratio of broken/intact sister chromatids in WT and *XRCC1* ^−/−^ RPE cells incubated or not as indicated in 20 μM olaparib (PARPi) for 4 h prior to and including the CldU pulse label. Data information: (B, C, E) Data are the mean (±SEM) of three independent biological repeats, in which > 100 fibers (technical repeats) were scored per sample per experiment. Statistical significance was determined by one‐way ANOVA with Tukey's multiple comparisons *post hoc* test.***P < 0.001. Source data are available online for this figure.

To test more directly whether UNG‐induced BER intermediates in template DNA strands trigger replication fork collapse, we employed DNA combing. Thus, we cultured RPE‐1 cells for one cell cycle in the presence of CldU, and then in the second cell cycle pulse‐labeled DNA replication tracks with IdU to detect their progression on ClU‐containing template strands (Fig 6A). Replication fork collapse on a broken ClU‐containing template strand was indicated in these experiments by the detection of a broken sister chromatid containing an IdU‐labeled tract (nascent DNA; pale green) located at one end of a CldU‐labeled tract (template DNA; red; see Fig 6A, top cartoon). In contrast, intact replication forks on unbroken ClU‐containing template strands were indicated by the detection of an intact sister chromatid containing an IdU‐labeled tract (nascent DNA; pale green) flanked by two CldU‐labeled tracts (template DNA; red; see Fig 6A, bottom cartoon). In the absence of PARPi, ~50% of sister chromatids were broken, which likely reflects mechanical breakage during the combing procedure (Fig 6B). More importantly, olaparib treatment for either 1.5 or 4 h prior to analysis significantly increased the fraction of broken sister chromatids, and this increase was ablated by UNG loss (Fig 6B). A significant increase in broken sister chromatids was also observed in CldU‐treated *XRCC1* ^−/−^ RPE‐1 cells, and this increase was similarly ablated by UNG loss (Fig 6C).

To confirm that the increase in broken sister chromatids detected in the above studies resulted from BER‐induced breaks present in DNA template strands, we exploited our observation that IdU‐labeled DNA was not cytotoxic and thus not a substrate for UNG (see Fig 1D). We thus swapped the order of CldU and IdU labeling and examined whether either PARP inhibition or XRCC1 loss altered the number of broken sister chromatids arising from the replication of IdU‐containing template DNA (Fig 6D). As predicted, neither XRCC1 deletion nor olaparib treatment increased the number of broken sister chromatids during the replication of IdU‐containing template DNA, despite the presence of UNG substrate (ClU) in the nascent strands (Fig 6E). These data confirmed not only that UNG‐induced BER intermediates are a source of replication fork breakage, but also that these intermediates must be present in template DNA strands to cause this breakage.

### CldU increases olaparib sensitivity in BRCA1‐ and BRCA2‐defective cells

**Figure 7:**
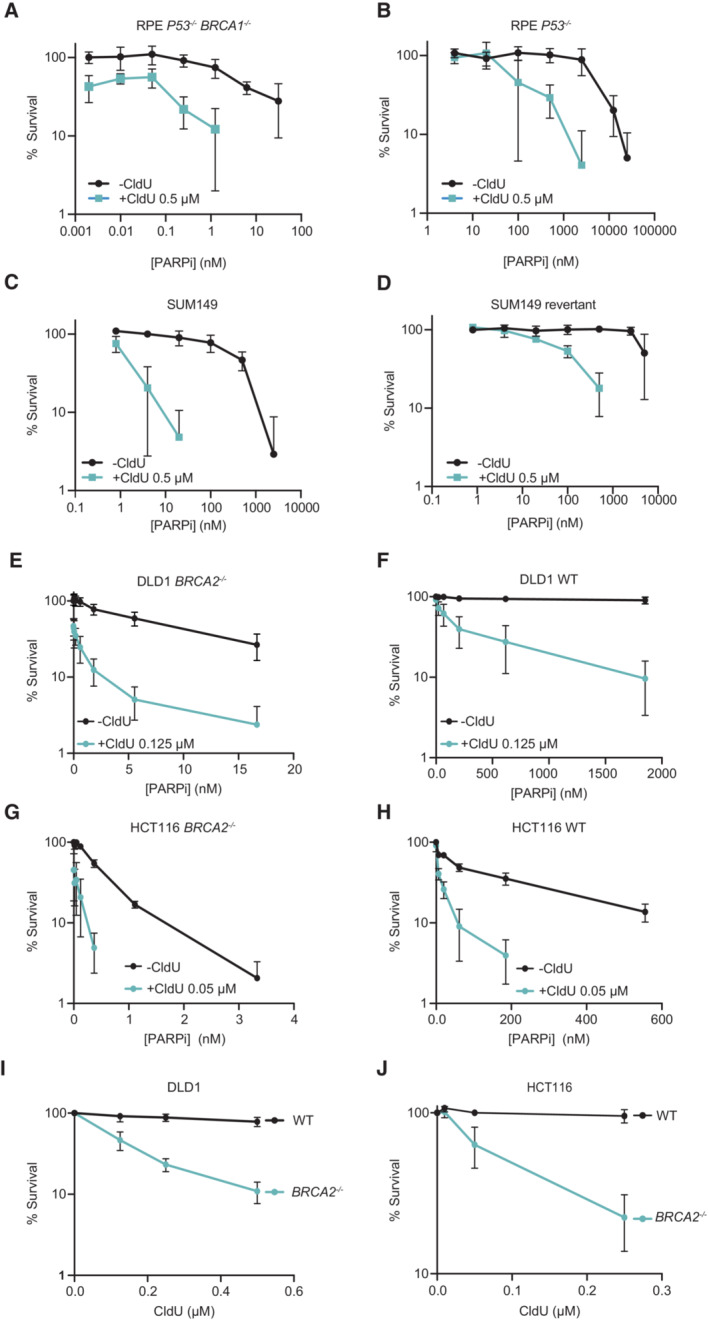
Hypersensitivity of BRCA1‐defective cells to a combination of sublethal concentrations of CldU and olaparib A–DClonogenic survival of *P53* ^−/−^ *BRCA1* ^−/−^ RPE‐1 cells (A), *P53* ^−/−^ RPE‐1 cells (B), BRCA1‐mutated SUM149 cells (C), and isogenic BRCA1‐revertant SUM149 cells (D), to continuous treatment with 0.5 μM CldU and/or the indicated concentrations of olaparib.E–HSurvival of DLD1 *BRCA2* ^−/−^ cells (E), DLD1 WT cells (F), HCT116 *BRCA2* ^−/−^ cells (G) and HCT116 WT cells (H), to 10–14 days continuous treatment with 0.125 or 0.05 μM CldU, respectively, and/or the indicated concentrations of olaparib. Cell survival was determined by Cell Titer Glo (Promega) (E,F) or by clonogenic survival (G,H).I, JSurvival of WT or *BRCA2* ^−/−^ DLD1 (I) or HCT116 (J) cells to 10–14 days continuous treatment with the indicated concentrations of CldU. Data are the means (±SD) of at least four independent biological (I) and technical (J) replicates. Cell survival was determined by Cell Titer Glo (Promega). Data information: (A–J) Data are the means (±SD) of 3 (A–H) or 4 (I) independent biological repeats. (J) are the means (±SD) of four dishes (technical replicates) from one biological replicate. Source data are available online for this figure.

Given the lethal impact of PARP inhibition during the replication of chlorouracil‐containing template DNA, we examined whether CldU might further sensitize BRCA‐defective cells to olaparib. Indeed, very low concentrations of olaparib (< 1 nM) that alone were not toxic in BRCA1‐deficient RPE‐1 cells, in accord with previous findings (Dev *et al*, 2018), were toxic to these cells if applied in the presence of 0.5 μM CldU (Fig 7A). In contrast, this drug combination was not toxic in BRCA1‐proficient RPE‐1 cells even if we employed up to 10‐fold higher concentrations (≤ 10 nM) of olaparib (Fig 7B). Similar results were observed for the BRCA1‐mutated cancer cell line SUM149PT (Elstrodt *et al*, 2006), in which otherwise sublethal concentrations of olaparib (≤ 10 nM) were selectively toxic in BRCA1‐deficient cells in the presence of 0.5 μM CldU (Fig 7C and D). Finally, we examined whether CldU might also increase PARPi sensitivity in BRCA2‐defective cells. Indeed, similar to BRCA1‐defective cells, olaparib concentrations (≤ 5 nM) that were largely sublethal in both BRCA2‐proficient and BRCA2‐deficient DLD1 cells when applied alone were selectively toxic to BRCA2‐deficient cells if combined with low concentrations of CldU (0.125 μM; Fig 7E and F). This was also true in BRCA2‐deficient HCT116 cells, albeit at much lower concentrations of olaparib (≤ 0.5 nM; Fig 7G and H). Notably, CldU was also selectively toxic to BRCA2‐defective cells even in the absence of olaparib (Fig 7I and J). Collectively, these data suggest that combinations of PARP inhibitor and/or CldU may provide novel therapeutic opportunities in BRCA1/2‐deficient settings.

## Discussion

We describe here an exquisite sensitivity of SSBR‐defective human cells to the thymidine analogue 5‐chloro‐2′‐deoxyuridine (CldU), induced by loss of either PARP activity or XRCC1. Such sensitivity was reported in CHO cells 30 years ago and was employed to clone the human XRCC1 gene (Dillehay *et al*, 1984; Thompson *et al*, 1990). Here, we have established the mechanism by which CldU induces hypersensitivity in SSBR‐defective cells, and we show that unrepaired BER intermediates in template DNA strands collapse DNA replication forks, leading to sister chromatid breakage. Consistent with this idea, the SSBR‐defective cells that were most sensitive to CldU were those resulting from PARP inhibition or lacking both PARP1 and PARP2, perhaps reflecting the requirement for PARP activity both for SSBR and for DNA replication fork protection (Chaudhuri & Nussenzweig, 2017; Hanzlikova & Caldecott, 2019). While previous studies have also addressed the impact of BER intermediates on DNA replication (Saleh‐Gohari *et al*, 2005; Ensminger *et al*, 2014), none to our knowledge has employed an approach that can discriminate between the impact of these intermediates when located in nascent DNA strands behind replication forks with those located in template DNA strands ahead of replication forks.

CRISPR genetic screens identified the uracil DNA glycosylase, UNG, as essential for triggering hypersensitivity to CldU in cells lacking PARP activity or XRCC1. This identifies unrepaired BER intermediates as the cytotoxic lesions. More importantly, our data implicate BER reactions arising close to DNA replication forks as the source of the cytotoxic SSBs, because UNG activity is S phase dependent and is coupled to the DNA replication machinery via interaction with PCNA (Otterlei *et al*, 1999; Hardeland *et al*, 2007). Consistent with this, UNG‐induced PARP activity following ClU incorporation was detected only in S phase. Importantly, we detected similar levels of UNG‐induced PARP activity during the S phase in which ClU was incorporated and during the S phase that followed, demonstrating that UNG‐induced BER occurs both in nascent DNA strands (during the first S phase) and in template DNA strands (during the second S phase). It is perhaps surprising that high levels of ClU persist into the second S phase in wild type cells, given the localization and rapidity of UNG‐induced BER behind DNA replication forks. This likely reflects the high level of ClU that is incorporated into genomic DNA, and the >100‐fold lower efficiency of UNG on this thymine analogue when compared to its canonical substrate, uracil. Indeed, we found that the amount of genomic ClU in wild‐type RPE‐1 cells was only ~25% less than that in *UNG* ^−/−^ RPE‐1 cells, 30 h after CldU incorporation (see Fig 4B), suggesting that ~75% of genomic CldU persisted into the following cell cycle even in UNG‐proficient cells.

UNG has been implicated previously in hypersensitivity to CldU, but it was concluded that this reflected thymidylate synthase inhibition and a consequent increase in uracil misincorporation, rather than direct excision of ClU by UNG (Brandon *et al*, 2000). This alternative scenario cannot explain our data, however, because neither PARP inhibition nor XRCC1 deletion greatly increased sensitivity to FldU, which is an equally if not more potent inhibitor of thymidylate synthase (Longley *et al*, 2003). Rather, we demonstrated here that ClU is indeed actively removed by UNG, albeit with >100‐fold lower efficiency than its canonical substrate, uracil. In contrast to CldU, neither PARP inhibition nor XRCC1 deletion increased sensitivity to the related thymidine analogues BrdU, FldU, or IdU, suggesting that the activity of UNG on these halogenated nucleotides is too low to generate SSBs at levels that are cytotoxic.

Critically, only UNG‐induced SSBs present during the S phase that followed ClU incorporation triggered DSBs and SCEs, suggesting that only unrepaired BER intermediates present in template DNA strands were cytotoxic. This, in turn, suggests that it is SSBs located ahead of DNA replication forks that trigger cell death, rather than those located behind forks, a conclusion consistent with our discovery that ClU increased the collapse of DNA replication forks only if present in DNA template strands. It should be noted, however, that our experiments measured fork collapse only in homologous recombination (HR)‐proficient cells, and that a different scenario may exist in BRCA‐defective cells in which post‐replicative gap filling by HR is reduced (Cong *et al*, 2021; Panzarino *et al*, 2021; Cong & Cantor, 2022). However, our data are consistent with the reported trans‐cell cycle impact of PARP inhibition in BRCA‐defective cells, in which single‐strand gaps formed behind DNA replication forks require a second S phase for DSB formation and cytotoxicity (Simoneau *et al*, 2021).

Although CldU resulted in elevated PARP activity throughout the cell cycle in *XRCC1* ^−/−^ cells, UNG was required for this PARP activity only during S phase. The CldU‐induced PARP activity detected in G1 and G2 most likely reflects another uracil DNA glycosylase (Krokan *et al*, 2002; Hardeland *et al*, 2007), with TDG being the most likely candidate because this enzyme is active specifically in G1/G2 (Hardeland *et al*, 2007). Importantly, the toxicity of CldU in SSBR‐defective cells was triggered entirely by UNG, indicating that the SSBs that were induced independently of UNG in G1/G2 were not cytotoxic and thus did not persist long enough to enter the following S phase. This likely reflects that, following ClU excision, SSBs are rapidly repaired even in SSBR‐defective cells. Consistent with this idea, although the half‐life of SSBs is increased 3–6‐fold (to ~30–90 min) in XRCC1‐defective cells (Thompson *et al*, 1990; Zdzienicka *et al*, 1992), this is still fast enough for most cells in G1/G2 to re‐join SSBs prior to their encounter with a DNA replication fork in the following S phase.

**Figure 8:**
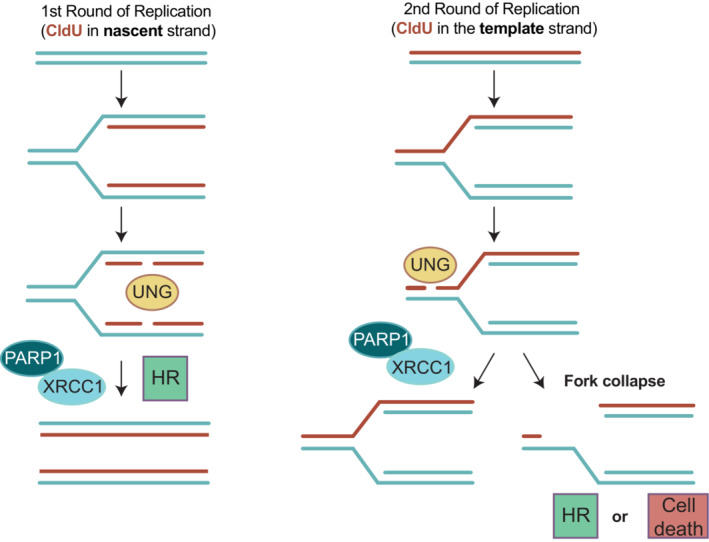
Model for the cytotoxic impact of UNG‐dependent BER intermediates in S phase *Left*, during the first round of replication in the presence of CldU, UNG initiates BER reactions behind DNA replication forks creating SSB intermediates that are rapidly repaired by PARP1/XRCC1‐dependent SSBR. In cells lacking XRCC1 or PARP1 activity these intermediates are repaired by alternative pathways, such as homologous recombination (HR)‐mediated gap repair. *Right*, during the subsequent S phase following CldU treatment, UNG initiates BER reactions on template strands ahead of DNA replication forks, creating SSB intermediates that are again rapidly repaired by PARP1/XRCC1‐dependent SSBR. In the absence of XRCC1 or PARP1 activity, however, the unrepaired SSB intermediates trigger DNA replication fork collapse and DSBs, inducing HR‐mediated DSB repair and SCE formation in HR‐proficient cells and cell death in HR‐deficient cells (e.g., BRCA1/BRCA2‐mutated).

Based on our data, we present a model in which UNG can excise ClU both ahead and behind DNA replication forks (Fig 8). This idea is consistent with previous biochemical experiments addressing the impact of RPA on UNG activity (Weiser *et al*, 2018). We propose that at physiological levels of uracil misincorporation, most if not all uracil is rapidly excised from nascent DNA strands behind DNA replication forks, a notion consistent with the observed localization of UNG behind DNA replication forks (Bjørås *et al*, 2017). Only in the presence of very high levels of substrate is UNG activity employed ahead of forks, such as following incubation with CldU as described here or perhaps in rare instances where uracil arises very close to an approaching fork as a result of “spontaneous” cytosine deamination. This is an important concept because it means that the opportunity for UNG activity immediately ahead of a DNA replication fork is limited, thereby minimizing the chance of DNA replication fork collapse.

Finally, our findings have important implications both for the use of CldU for DNA replication studies and for the possible utility of this nucleoside in cancer therapy. With respect to DNA replication studies, our data demonstrate that CldU incorporation leads rapidly to UNG‐induced incision of DNA in S phase, and thus where possible should be avoided. This is particularly true for experiments involving long time‐courses in which incorporated ClU persists into a second S phase. In contrast, IdU triggers relatively little BER and is thus a more appropriate thymidine analogue for the study of DNA replication dynamics. With respect to cancer therapy, PARP inhibitors are effective treatments for ovarian, breast, pancreatic, and prostate cancers with mutations in BRCA1 or BRCA2 (Fong *et al*, 2009; Tutt *et al*, 2021). We found that low concentrations of olaparib and CldU that are non‐toxic when applied separately are selectively toxic in BRCA1‐ and BRCA2‐defective cells if applied together. These data thus raise the possibility that low concentrations of CldU might improve the clinical efficacy of PARP inhibitors, perhaps enhancing the selective killing of cancer cells and reducing the likelihood of developing resistance. Intriguingly, our data also suggested that, in the case of BRCA2‐defective cancer cells at least, treatment with CldU alone may be a promising monotherapy.

## Materials and Methods

### Cell lines

Cell lines were checked regularly for mycoplasma contamination and authenticated by PCR analysis. Human RPE‐1 hTERT (referred to as RPE‐1), U2OS and SUM149PT cells were grown at 37°C, 5% CO_2_. RPE‐1 cells were cultured in Dulbecco's Modified Eagle Medium: Nutrient Mixture Ham's F‐12 (DMEM/F‐12, Sigma‐Aldrich), 10% (v/v) fetal bovine serum (FBS, BioSera), 100 U/ml penicillin, 100 μg/ml streptomycin (Sigma‐Aldrich), and 2 mM L‐glutamine. 10 μg/ml blasticidin (Sigma‐Aldrich) was used to select for Cas9 expressing cells. Cells were additionally cultured with 1.5 μg/ml of puromycin during the selection of the transductants in the CRISPR‐Cas9 screens. SUM149PT cells were cultured in Ham's F12 with 5% fetal bovine serum (FBS, BioSera), insulin (5 μg/ml), HEPES and hydrocortisone (1 μg/ml). DLD1 were cultured in RPMI 1640 medium supplemented with sodium bicarbonate, 10% (v/v) fetal bovine serum (FBS, BioSera), 100 U/ml penicillin, 100 μg/ml streptomycin (Sigma‐Aldrich), and 2 mM L‐glutamine. HCT116 were cultured in McCoy's 5A (Modified) Medium supplemented with 10% (v/v) fetal bovine serum (FBS, BioSera), 100 U/ml penicillin, 100 μg/ml streptomycin (Sigma‐Aldrich), and 2 mM L‐glutamine.

The generation of *XRCC1* ^−/−^ (clone #3), *XRCC1* ^−/−^/*PARP1* ^−/−^ (clone #D1), *PARP1* ^−/−^ (clone #G7) *PARP2* ^−/−^ (clone #A1), *PARP1* ^−/−^ */PARP2* ^−/−^ (clone #E6) RPE‐1 cells, and *XRCC1* ^−/−^ U2OS cells have been described elsewhere (Hanzlikova *et al*, 2017; Hoch *et al*, 2017; Polo *et al*, 2019). For generating *UNG* ^−/−^, *SMUG1* ^−/−^ and *MBD4* ^−/−^ RPE‐1 cells, 0.6 × 10^5^ RPE‐1 cells expressing Cas9 were seeded in 60 mm plates. Cells were transfected with a sgRNA targeting each gene (5′‐CAAAGCCCACGGGCACGTTG‐3′, 5′‐GGATTGTAGATGATGCCCAC‐3′, 5′‐TGGGACTAGGCGCTCACTAG‐3′, respectively) using RNAiMAX (Invitrogen) according to manufacturer's protocol. After 48–72 h, single cells were sorted into 96‐well plates on a FACSAria III (BD Biosciences) or a MoFlo Astrios (Beckman Coulter). Successfully gene‐edited clones were selected on the basis of TIDE analysis (https://tide‐calculator.nki.nl; Brinkman *et al*, 2014) and confirmed, when possible, by immunoblotting. *XRCC1* ^−/−^ */UNG* ^−/−^ cells (clones #6, #20 and #27) were generated by co‐transfecting *XRCC1* ^−/−^ (clone #3) RPE‐1 cells with gRNA vector (Addgene #41824) encoding UNG gDNA (5′‐CAAAGCCCACGGGCACGTTG‐3′) and pCas9‐GFP vector (Addgene #48138) using LTX transfection reagent, according to manufacturer instructions. TDG RPE‐1 cells (clone #A4, #A10 and #C10) were generated by co‐transfecting RPE‐1 cells with CRISPR RNA (Merck, 5′‐UAUAGAUUGGCAUAAACCC‐3′ and 5′‐UGUAGUUCCAGCUACUACGG‐3′) spanning exon 4 of TDG, transactivating CRISPR RNA (tracrRNA; Merck, TRACRRNA05N), and recombinant His‐SpCas9‐GFP protein using a NEON transfection system, as described previously for *FEN1* ^−/−^ cells (Vaitsiankova *et al*, 2022). 48 h later, single GFP‐positive cells were sorted in to 96‐well plates using FACS melody and individual clones were validated by Western blotting and, where indicated, by direct Sanger sequencing of PCR products.

### Chemicals

The chemicals used were PARPi (olaparib; ApexBio, A4154) and PARGi (Tocris, PDD 00017273) dissolved in DMSO at stock solution of 10 mM. BrdU (Merck, B5002), CldU (Merck, C6891), FldU (Merck, F0503), and IdU (Merck, I7125) were dissolved in DMEM‐F12 media at stock solution of 2.5 mM.

### Commercial antibodies

The antibodies employed for this work were as follows. Primary: Rat anti‐CldU (BioRad, OBT0030G; 1:200), mouse anti‐H2AX‐phosphoSer139 (Millipore, clone JBW301; 1:500) rat anti‐BrdU (Abcam, ab6326; 1:30), mouse anti‐BrdU (Becton Dickinson, 347580; 1:25), mouse anti‐single‐strand DNA (Millipore, MAB3034; 1:25), mouse anti‐UNG (Origene, TA503755; 1:500), rabbit anti‐vinculin (Abcam, ab91459; 1:5,000), rat anti‐tubulin (Abcam, ab6160; 1:5,000). Secondary: Goat anti‐rat Alexa Fluor 488 (Thermo Fisher, A11006; 1:1,000), donkey anti‐mouse Alexa Fluor 568 (Thermo Fisher, A10037; 1:1,000), goat anti‐mouse Alexa Fluor 488 (Thermo Fisher, A32723; 1:25), donkey anti‐rabbit Alexa Fluor 488 (Thermo Fisher, A21206; 1:1,000), goat anti‐rat Alexa Fluor 568 (Thermo Fisher, A11077; 1:25), donkey anti‐mouse Alexa Fluor 647 (Thermo Fisher, A31571; 1:25).

### Cloning and purification of recombinant macro (Af1521) ^MG^‐Fc antibody

An ORF encoding rabbit IgG Fc chain was codon optimized, synthesized (IDT) and cloned into pET28a vector with the addition of an ORF encoding the Af1521 macrodomain at the C‐terminus. The Af1521 macrodomain was subcloned from the plasmid pGEX4‐Af1521, which was kindly provided by Michael Nielsen. Site‐directed mutagenesis of Af1521 was performed using QuikChange Lightning Multi Site‐Directed Mutagenesis Kit (Agilent Technologies) to introduce the “MacroGreen” mutations K35E, G42E, I144R, and Y145R (Nowak *et al*, 2020; García‐Saura *et al*, 2021). The recombinant antibody was denoted Macro(Af1521)^MG^‐Fc, in which “MG” denotes the presence of the four “MacroGreen” mutations. Macro(Af1521)^MG^‐Fc was expressed in *E. coli* BL21 (DE3) by induction with 0.1 mM IPTG at 16°C overnight. Cells were resuspended in lysis buffer (10 mM Tris–HCl pH 7.5, 0.5 M NaCl, 0.1 mM EDTA, 0.1% NP‐40, 10% glycerol, 10 mM imidazole, 1 mM DTT) and sonicated. Cell lysate was clarified by centrifugation and the antibody was purified using Ni‐NTA beads (Qiagen). Beads were washed extensively by lysis buffer and washing buffer (10 mM Tris–HCl pH 7.5, 0.2 M NaCl, 30 mM imidazole, 1 mM DTT), bound proteins were eluted with elution buffer (10 mM Tris–HCl pH 7.5, 0.2 M NaCl, 500 mM imidazole, 1 mM DTT) and dialyzed overnight to dialysis buffer (10 mM Tris–HCl pH 7.5, 0.2 M NaCl, 10% glycerol, 1 mM DTT).

### Purification of recombinant wild type and mutant (D154N) human UNG

Plasmids encoding wild‐type UNG (pET28‐UNG) and UNG with a mutation in the catalytic domain (pET28‐UNG^D154N^) were kindly provided by Bodil Kavli (Norwegian University of Science and Technology; Scaramozzino *et al*, 2003). pET28‐UNG harbored a small deletion at the 5′‐end of the UNG ORF, which we corrected accordingly. The plasmids were transformed into the bacterial strain NiCo21(DE3) (NEB, C2529H) and grown at 37°C in TurboBroth (Molecular Dimensions, Sheffield, UK) to an OD_600_ of ~2.0. The cells were then chilled on ice for 15 min and expression induced at 30°C for 3 h by the addition of IPTG (1 mM). Cells were then resuspended in 80 ml Buffer A (20 mM Hepes pH 7.8, 500 mM NaCl, 5% glycerol, 0.2 mM DTT, 1 mM PMSF, 50 mM imidazole and protease inhibitors (Complete EDTA free, Roche) and sonicated. Cell lysate was clarified by centrifugation and the supernatant was collected, filtered, and added to 2 ml of Ni^2+^‐agarose beads (Cytiva, 17531801) at 4°C by gravity flow. The columns were washed with 25 column volumes of Buffer A, 7 column volumes of Buffer B (20 mM Hepes pH 7.8, 1 M NaCl, 5% glycerol, 0.2 mM DTT, 1 mM PMSF, 50 mM imidazole) and 25 column volumes of buffer A. UNG proteins were eluted in Elution Buffer (20 mM Hepes pH 7.8; 500 mM NaCl, 5% glycerol, 0.2 mM DTT, 150 mM imidazole) and snap frozen in liquid nitrogen. Peak protein fractions were further purified by gel filtration (Superdex 200 increase 10/300) on an Akta Pure system, in 20 mM Hepes pH 7.8, 500 mM NaCl, 1 mM DTT. The three peak 0.5 ml fractions were pooled and dialysed against 20 mM Tris–HCl pH 7.8 at 4°C, 150 mM NaCl, 5% glycerol, 1 mM DTT. The proteins were snap frozen in liquid nitrogen and stored in −80°C until use.

### Synthesis of CldU substrate

The nucleoside phosphoramidite of 5‐chloro‐2′‐deoxyuridine was synthesized as described (Münzel *et al*, 2011; Riml *et al*, 2017). Briefly, 5‐chloro‐2′‐deoxyuridine was dimethoxytritylated at the 5′ hydroxyl group using dimethoxytrityl chloride and 4‐dimethylaminopyridine in anhydrous pyridine, stirring at room temperature for 16 h. The intermediate was purified by flash column chromatography and was then phosphitylated at the 3′ hydroxyl group using 2‐cyanoethyl N,N‐diisoproylchlorophosphoramidite, DIPEA and 1‐methylimidazole in dichloromethane, stirring at 0°C – > room temperature for 16 h. The crude product was purified by flash column chromatography.

Solid phase synthesis of oligonucleotides was performed on an ABI394 Synthesizer using phenoxyacetyl (Pac) protected dA and 4‐isopropyl‐phenoxyacetyl (iPr‐Pac) protected dG (Glen Research), with other reagents supplied by Biosearch Technologies. Deprotection was achieved by treatment with ammonium hydroxide for 2 h at room temperature. Oligonucleotides were purified by reversed phase HPLC using a linear gradient of acetonitrile in 0.1 M triethylamine acetate using a Clarity 5 μm oligo‐RP column (Phenomenex).

### UNG activity assays

UNG activity was measured using a 21‐mer oligodeoxynucleotide: 5′ GTGAAATTGT**U**ATCCGCTCAG 3′ (U substrate) and 5′ GTGAAATTGT**CldU**ATCCGCTCAG 3′ (CldU substrate). The oligos were tagged with 5‐Iodoacetamidofluorescein (5‐IAF) as described (Zearfoss & Ryder, 2012). The oligos were then annealed with the complementary sequence (5′ CTGAGCGGATAACAATTTCAC 3′). 2 pmoles were incubated with the indicated amounts of UNG and UNG buffer (20 mM Tris‐Cl (pH 8.0), 1 mM EDTA, 1 mM DTT, 0.1 mg/ml BSA) for the indicated times. The reaction was stopped by adding NaOH (final concentration 200 mM) and TBE Urea Sample Buffer (2×), and the samples heated at 95°C for 10 min and then chilled on ice. The samples were loaded and resolved on 15% polyacrylamide–urea TBE gel, and the fluorescein‐labeled fragments imaged in the gel with a Typhoon Amersham 5 imager.

### Survival assays

For RPE‐1, U2OS or SUM149 cells, 300 cells were seeded per 10 cm dish/well (6‐well plate) in duplicate and 4 h/24 h later were continuously treated with the indicated concentrations of thymidine analogue (BrdU, CldU, IdU, FldU). Where stated, cells were continuously treated with 0.5 μM olaparib. Cells were grown for 7–12 days (or until colonies were visible), followed by fixation in ethanol and staining with 0.05% crystal violet. For each cell line, the percentage clonogenic survival was calculated by dividing the average number of colonies (> 50 cells) at each dose by the average number of colonies in the untreated condition. For HCT116 cells, 450 cells (WT) or 1,500 cells (BRCA2 deficient) were seeded per well/6‐well plates and 24 h later were continuously treated with the indicated concentrations of CldU and olaparib for 10 days followed by fixation in ethanol and staining with 0.05% crystal violet. For each cell line, the percentage survival was calculated by dividing the average stained area at each dose by the average stained area in the untreated condition. For DLD1, 150 cells (WT) or 450 cells (BRCA2‐deficient) were seeded per well in 96‐well plates and 24 h later were continuously treated with the indicated concentrations of CldU and olaparib for 11–14 days followed by cell viability estimation using Cell‐Titer Glo (Promega).

### Imaging and quantification of CldU/IdU immunofluorescence

Cells were seeded at 1.5 × 10^5^ per 6‐well plate on 13 mm coverslips and the next day treated or not with CldU (1 or 10 μM) or IdU (10 μM). Cells were washed with PBS, fixed in 4% formaldehyde for 15 min, followed by two washes in PBS and denaturation (2 M HCl, 0.5% Triton X‐100) for 30 min. Next, cells were neutralized in 0.1 M Na_2_B_4_O_7_ for 5 min, followed by two washes in PBS. Cells were blocked in BSA in PBS for 1 h followed by incubation with primary rat anti‐BrdU (CldU; Abcam, ab6326, 1:200) or mouse anti‐BrdU (IdU; Becton Dickinson, 347580, 1:200) for 2 h and extensive washing. Samples were incubated in secondary goat anti‐rat Alexa Fluor 568 (Thermo Fisher, A11077; 1:1,000) or goat anti‐mouse Alexa Fluor 488 (Thermo Fisher, A32723; 1:1,000) for 1 h, followed by extensive washing. DNA was counterstained with DAPI and the coverslips were mounted using Mowiol (Sigma Aldrich) mounting media. Images were acquired using an automated Olympus ScanR system, a motorized Olympus IX81 microscope with 40×/0.6 (LUCPLFLN 40× PH) dry objectives and Hamamatsu ORCA‐R2 digital CCD camera C10600. Images were collected and analyzed using ScanR automated software to quantify the fluorescence intensity of individual cell nuclei. All quantification was conducted on cells/samples (typically > 1,000 cells per sample per experiment) selected by scanR software in a random/unbiased manner.

### Sister chromatid exchange

Cells were treated as required, arrested in metaphase with 100 ng/ml colcemid (KaryoMax Gibco) for the last 2–3 h of the experiment, swollen in 75 mM KCl for 5 min and fixed in Carnoy's fixative (75% Methanol:25% Acetic Acid) before the preparation of metaphase spreads. For CldU labeling, slides were incubated in 2 M HCl for 30 min, washed twice in 100 mM borate buffer pH 8.5 for 10 min, blocked in 2% BSA in PBS for 30 min, and incubated with primary rat anti‐CldU (BioRad OBT0030G, 1:200) at 4°C overnight. Slides were washed in PBS, incubated with secondary goat anti‐rat Alexa Fluor 488 (Thermo) for 1 h. For EdU labeling, metaphase spreads were incubated with click chemistry reaction mix for 30 min, as described above. Slides were stained with DAPI for 10 min, mounted using Vectashield (VectorLabs) and the images were acquired using a TissueFAXS i‐Fluo system (TissueGnostics), first with a 20× objective to identify adequate metaphases, then with a 63× oil objective for high‐resolution image acquisition. For experiments with RPE cells, sister chromatid exchange events were counted manually for 15 full metaphases (containing at least 42 of the expected 46 clearly identifiable chromosomes) per replicate and the number of SCEs per metaphase plotted. For U2OS cells, the number of SCEs was counted for at least 500 chromosomes per replicate, and the results expressed as a ratio of SCEs/chromosome.

### γH2AX foci analysis

Cells were grown on 1.5H glass coverslips (Thorlabs) treated as required, pulse‐labeled with 10 μM EdU for the last 45 min and fixed with 4% PFA in PBS for 10 min. After two washes in 0.1 M glycine in PBS and permeabilization in 0.2% Triton–X 100 in PBS for 10 min, samples were blocked in 10% fetal bovine serum in 0.2% Triton in PBS for 30 min. The incorporated EdU was labeled with Alexa Fluor 488 azide (Thermo) in a click chemistry reaction containing 100 mM Tris pH 8.5, 1 mM copper sulphate, 100 mM ascorbic acid and 1 μM AF488 azide for 30 min. Samples were incubated with primary mouse anti‐H2AX‐phosphoSer139 (clone JBW301, Millipore, 1:500) for 2 h at room temperature, washed extensively in PBS, incubated with donkey anti‐mouse Alexa Fluor 568 (Thermo, 1:1,000) for 1 h, washed, stained with DAPI and the coverslips mounted in VectaShield (VectorLabs). Images were acquired on a customized TissueFAXS i‐Fluo system (TissueGnostics) mounted on a Zeiss AxioObserver 7 microscope (Zeiss) using a 20× Plan‐Neofluar objective (NA 0.5). Images were analyzed using StrataQuest software (TissueGnostics), gating correctly detected cells based on nuclear area and DAPI intensity. EdU‐positive populations were determined based on EdU vs DAPI scatterplots, and the number of γH2AX foci per nucleus was determined using the “dot detection” algorithm. Alternatively, γH2AX foci were imaged and quantified using ScanR automated software. The mean number of yH2AX foci per EdU‐positive nucleus was determined for thousands of cells per sample per experiment.

### CRISPR‐Cas9 screen

For the genome‐wide CRISPR‐Cas9 screen, two different clones of RPE‐1 TP53^KO^ Cas9 expressing cells were transduced at an MOI of 0.2 and 500‐fold coverage of the Brunello whole‐genome sgRNA library (Doench *et al*, 2016). Afterwards, transductants were selected with puromycin for 8 days before treatment with either DMSO, olaparib (500 nM) or olaparib (500 nM) + CldU (0.2 μM) for 15 days. Cells were subcultured every 3 days. Genomic DNA from cell pellets collected at the pre‐treatment and last treatment days was isolated using the QIAamp Blood Maxi Kit (Qiagen) and genome‐integrated sgRNA sequences were amplified by PCR using the Q5 Mastermix (New England Biolabs Ultra II) and i7 multiplexing barcoded primers. The final gel‐purified products were sequenced on Illumina HiSeq4000 system. Guide‐enrichment analysis was performed using DrugZ (Colic *et al*, 2019) to compare the different conditions.

### Immunoblotting

Cells were lysed in Laemmli buffer, boiled at 96°C for 5 min. Protein extracts were resuspended using a 29G needle and a 0.5 ml syringe. Equivalent amounts of protein (∼20 μg) were run in NuPAGE 4–12% Bis‐Tris Precast Protein Gels (Invitrogen). Then samples were transferred to 0.45 μm PVDF Blotting Membrane (Merck) o/n at 4°C, 50 mA. Membranes were blocked in 5%BSA‐TBST (TBS‐0.1% Tween 20) for 1 h, incubated with primary antibodies for 4 h at room temperature in 5%BSA‐TBST and washed (three times) in TBST. They were then incubated with the corresponding IRDye‐conjugated secondary antibodies (1/10,000 dilution) in 5%BSA‐TBST and washed (three times) in TBST and 1× in TBS buffer. Membranes were analyzed in Odyssey CLx with ImageStudio Odyssey CLx Software.

### Sample preparation for analysis of nucleotide composition by LC–MS

Cells (3 × 10^6^) were seeded in 150 mm plates, treated or not for 30 h with CldU (1 μM), collected, and DNA extracted using DNeasy Blood & Tissue Kit (Qiagen) supplemented with 10 μl RNAse (Roche). 2 μg genomic DNA was digested to mononucleosides by Nucleoside Digestion Mix (NEB), reactions were carried out in a total volume of 20 μl. After 1 h at 37°C, 80 μl of 50% Methanol were added to each sample which were spun for 15′ at 20,000 *g*. Supernatants were transferred to metabolomics vials and analyzed by LC–MS.

### LC–MS

Chromatographic separation of metabolites was achieved using a Millipore Sequant ZIC‐pHILIC analytical column (5 μm, 2.1 × 150 mm) equipped with a 2.1 × 20 mm guard column (both 5 mm particle size) with a binary solvent system. Solvent A was 20 mM ammonium carbonate, 0.05% ammonium hydroxide; Solvent B was acetonitrile. The column oven and autosampler tray were held at 40°C and 4°C, respectively. The chromatographic gradient was run at a flow rate of 0.200 ml/min as follows: 0–2 min: 80% B; 2–17 min: linear gradient from 80% B to 20% B; 17–17.1 min: linear gradient from 20% B to 80% B; 17.1–23 min: hold at 80% B. Samples were randomized and the injection volume was 5 μl. A pooled quality control (QC) sample was generated from an equal mixture of all individual samples and analyzed interspersed at regular intervals. Metabolites were measured with Vanquish Horizon UHPLC coupled to an Orbitrap Exploris 240 mass spectrometer (both Thermo Fisher Scientific) via a heated electrospray ionization source. The spray voltages were set to +3.5 kV/−2.8 kV, RF lens value at 70, the heated capillary held at 320°C, and the auxiliary gas heater held at 280°C. The flow rate for sheath gas, aux gas, and sweep gas were set to 40, 15 and 0, respectively. Data acquisition was performed in full scan mode with polarity switching at an Orbitrap resolution of 120,000, with mass range set to *m/z* = 70–900, AGC target set to standard and maximum injection time (Max IT) set to auto. Metabolite identities were confirmed using two parameters: (i) precursor ion m/z was matched within 5 ppm of theoretical mass predicted by the chemical formula; (ii) the retention time of metabolites was within 5% of the retention time of a purified standard run with the same chromatographic method. Chromatogram review and peak area integration were performed using the Thermo Fisher software Tracefinder 5.0 and the peak area for each detected metabolite was normalized against the total ion count (TIC) of that sample to correct any variations introduced from sample handling and instrument analysis. The normalized areas were used as variables for further statistical data analysis.

### Poly(ADP‐ribose) immunofluorescence

Cells were seeded at 1.5 × 10^5^ per 6‐well plate on 13 mm coverslips and the next day treated or not with CldU (1 or 10 μM). Where indicated, cells were treated with 10 μM EdU to pulse label cells and/or 20 μM PARGi to preserve nascent poly(ADP‐ribose) for the final 30 min. Cells were washed with PBS and incubated with pre‐extraction buffer (25 mM HEPES pH 7.4, 50 mM NaCl, 1 mM EDTA, 3 mM MgCl_2_, 0.3 M sucrose, 0.5% Triton X‐100) for 5 min on ice. Next, cells were fixed in cold 4% formaldehyde for 10 min, followed by two washes in PBS and permeabilization in 0.2% Triton‐X 100 in PBS for 5 min. Cells were blocked in BSA in PBS for 1 h followed by labeling of EdU using Click‐iT EdU Alexa 647 Imaging Kit (Invitrogen, C10640) following the manufacturer instructions. Cells were incubated for 2 h with recombinant Macro(Af1521)^MG^‐Fc antibody to detect ADP‐ribose, followed by incubation in donkey anti‐rabbit Alexa Fluor 488 (Thermo, 1:1,000) for 1 h. DNA was counterstained with DAPI and the coverslips were mounted using Mowiol (Sigma Aldrich) mounting media. Images were acquired using an automated Olympus ScanR system, a motorized Olympus IX81 microscope with 40×/0.6 (LUCPLFLN 40× PH) dry objectives and Hamamatsu ORCA‐R2 digital CCD camera C10600. Images were analyzed using ScanR analysis software to quantify the fluorescence intensity of individual cell nuclei and the cell cycle phases were determined by EdU and DAPI content. All quantification was conducted on cells/samples (typically > 1,000 cells per sample per experiment) selected by scanR software in a random/unbiased manner.

### DNA combing

RPE‐1 cells were labeled with 1 μM CldU/IdU for 24 h followed by a 1 h wash in label‐free media and a pulse‐label with 250 μM IdU/CldU for 30 min. Where indicated, cells were co‐treated with PARPi (20 μM olaparib) for the final 1.5 or 4 h. Next, cells were collected and resuspended in ice‐cold PBS to give a final concentration of 5 × 10^6^ cells/ml. 50 μl of this cell mix was pre‐warmed for 10 s at 50°C, combined with equal parts of molten low‐melting point agarose and pipetted into an agarose plug casting mold (BioRad). Once solidified, the plugs were transferred to round‐bottom tubes and incubated overnight at 42°C in proteinase K buffer (2 mg/ml proteinase K, 10 mM Tris–HCl pH 7.5, 100 mM EDTA, 0.5% SDS, 20 mM NaCl). Next, the DNA plugs are washed two times for 1 h in TE50 buffer (10 mM Tris–HCl pH 7.5, 50 mM EDTA, 100 mM NaCl) followed by two times for 1 h in TE buffer (10 mM Tris–HCl pH 7.5, 1 mM EDTA, 100 mM NaCl). The plug was then melted at 68°C for 20 min in 1 ml MES (35 mM MES hydrate, 150 mM MES sodium salt, 100 mM NaCl) and cooled to 42°C for 10 min. The samples were then incubated at 42°C overnight with the addition of 3 μl of β‐agarase (NEB). The DNA mix was then gently poured into combing reservoirs containing 1.2 ml MES and the genomic DNA was combed onto silanized coverslips (Genomic Vision) using a combing machine (Genomic Vision). Subsequently, the coverslips were baked for 2 h at 68°C followed by incubation in denaturing solution (0.5 M NaOH, 1 M NaCl) for 8 min. Next, the coverslips were dehydrated in 70, 90, and 100% ethanol for 1 min each, blocked for 1 h in 1% BSA in PBS, and then incubated with the antibodies listed below at 37°C in a humidified chamber. First, the coverslips were incubated with primary rat monoclonal anti‐BrdU (Abcam, ab6326; 1:30) and mouse monoclonal anti‐BrdU (Becton Dickinson, 347580; 1:25) for 1 h, followed by incubation with secondary goat anti‐mouse Alexa Fluor 488 (Thermo Fisher, A11001; 1:25) and goat anti‐rat Alexa Fluor 568 (Thermo Fisher, A11077; 1:25) for 45 min. The coverslips were then incubated with mouse monoclonal anti‐ssDNA antibody (Millipore, MAB3034; 1:25) for 2 h, followed with donkey anti‐mouse Alexa Fluor 647 (Thermo Fisher, A31571; 1:25) for 45 min. Between each antibody, the coverslips were washed three times for 3 min in PSBT (PBS with tween). Coverslips were then dehydrated in ethanol as above before mounting on microscope slides with fluoroshield (Merck). Finally, the slides were imaged using an Apotome widefield microscope (Zeiss) with ×40 oil objectives. ImageJ software was used to visualize, score, and measure the lengths of labeled replication tracks.

## Author contributions

**Almudena Serrano‐Benitez:** Conceptualization; data curation; formal analysis; investigation; methodology; writing – review and editing. **Sophie E Wells:** Conceptualization; data curation; formal analysis; investigation; methodology; writing – review and editing. **Lylah Drummond‐Clarke:** Investigation. **Lilian C Russo:** Methodology. **John Christopher Thomas:** Methodology. **Giovanna A Leal:** Investigation. **James Michael Edgerton:** Methodology. **Shankar Balasubramanian:** Methodology. **Ming Yang:** Methodology. **Christian Frezza:** Methodology. **Amit Gautam:** Methodology. **Jan Brazina:** Methodology. **Kamila Burdova:** Methodology. **Nicolas C Hoch:** Conceptualization; resources; data curation; formal analysis; supervision; funding acquisition; investigation; methodology; writing – review and editing. **Stephen P Jackson:** Conceptualization; resources; data curation; formal analysis; supervision; funding acquisition; investigation; methodology; writing – review and editing. **Keith W Caldecott:** Conceptualization; resources; data curation; formal analysis; supervision; funding acquisition; investigation; methodology; writing – original draft; project administration; writing – review and editing. **Mark Farrow:** Methodology.

## Disclosure and competing interests statement

The authors declare that they have no conflict of interest.

## Supporting information



Expanded View Figures PDF

PDF+

Source Data for Figure 1

Source Data for Figure 2

Source Data for Figure 3

Source Data for Figure 4

Source Data for Figure 5

Source Data for Figure 6

Source Data for Figure 7

## Data Availability

All materials, tools, and primary data are freely available from the corresponding authors on request. Screen data are available at Dryad (doi: 10.5061/dryad.dbrv15f67).

## References

[embj2022113190-bib-0001] Awwad SW , Serrano‐Benitez A , Thomas JC , Gupta V , Jackson SP (2023) Revolutionizing DNA repair research and cancer therapy with CRISPR–Cas screens. Nat Rev Mol Cell Biol 24: 1–18 36781955 10.1038/s41580-022-00571-x

[embj2022113190-bib-0002] Balmus G , Pilger D , Coates J , Demir M , Sczaniecka‐Clift M , Barros AC , Woods M , Fu B , Yang F , Chen E *et al* (2019) ATM orchestrates the DNA‐damage response to counter toxic non‐homologous end‐joining at broken replication forks. Nat Commun 10: 87 30622252 10.1038/s41467-018-07729-2 PMC6325118

[embj2022113190-bib-0003] Berti M , Chaudhuri AR , Thangavel S , Gomathinayagam S , Kenig S , Vujanovic M , Odreman F , Glatter T , Graziano S , Mendoza‐Maldonado R *et al* (2013) Human RECQ1 promotes restart of replication forks reversed by DNA topoisomerase I inhibition. Nat Struct Mol Biol 20: 347–354 23396353 10.1038/nsmb.2501 PMC3897332

[embj2022113190-bib-0004] Bitter EE , Townsend MH , Erickson R , Allen C , O'Neill KL (2020) Thymidine kinase 1 through the ages: a comprehensive review. Cell Biosci 10: 138 33292474 10.1186/s13578-020-00493-1 PMC7694900

[embj2022113190-bib-0005] Bjørås KØ , Sousa MML , Sharma A , Fonseca DM , Søgaard CK , Bjørås M , Otterlei M (2017) Monitoring of the spatial and temporal dynamics of BER/SSBR pathway proteins, including MYH, UNG2, MPG, NTH1 and NEIL1‐3, during DNA replication. Nucleic Acids Res 45: 8291–8301 28575236 10.1093/nar/gkx476 PMC5737410

[embj2022113190-bib-0006] Bowden AR , Morales‐Juarez DA , Sczaniecka‐Clift M , Agudo MM , Lukashchuk N , Thomas JC , Jackson SP (2020) Parallel CRISPR‐Cas9 screens clarify impacts of p53 on screen performance. Elife 9: e55325 32441252 10.7554/eLife.55325 PMC7244323

[embj2022113190-bib-0007] Brandon ML , Mi L‐J , Chaung W , Teebor G , Boorstein RJ (2000) 5‐Chloro‐2′‐deoxyuridine cytotoxicity results from base excision repair of uracil subsequent to thymidylate synthase inhibition. Mutat Res 459: 161–169 10725666 10.1016/s0921-8777(99)00061-0

[embj2022113190-bib-0008] Brinkman EK , Chen T , Amendola M , van Steensel B (2014) Easy quantitative assessment of genome editing by sequence trace decomposition. Nucleic Acids Res 42: e168 25300484 10.1093/nar/gku936 PMC4267669

[embj2022113190-bib-0009] Bryant HE , Schultz N , Thomas HD , Parker KM , Flower D , Lopez E , Kyle S , Meuth M , Curtin NJ , Helleday T (2005) Specific killing of BRCA2‐deficient tumours with inhibitors of poly(ADP‐ribose) polymerase. Nature 434: 913–917 15829966 10.1038/nature03443

[embj2022113190-bib-0010] Bryant HE , Petermann E , Schultz N , Jemth A‐S , Loseva O , Issaeva N , Johansson F , Fernandez S , McGlynn P , Helleday T (2009) PARP is activated at stalled forks to mediate Mre11‐dependent replication restart and recombination. EMBO J 28: 2601–2615 19629035 10.1038/emboj.2009.206 PMC2738702

[embj2022113190-bib-0011] Chaudhuri AR , Nussenzweig A (2017) The multifaceted roles of PARP1 in DNA repair and chromatin remodelling. Nat Rev Mol Cell Biol 18: 610–621 28676700 10.1038/nrm.2017.53 PMC6591728

[embj2022113190-bib-0012] Chaudhuri AR , Hashimoto Y , Herrador R , Neelsen KJ , Fachinetti D , Bermejo R , Cocito A , Costanzo V , Lopes M (2012) Topoisomerase I poisoning results in PARP‐mediated replication fork reversal. Nat Struct Mol Biol 19: 417–423 22388737 10.1038/nsmb.2258

[embj2022113190-bib-0013] Colic M , Wang G , Zimmermann M , Mascall K , McLaughlin M , Bertolet L , Lenoir WF , Moffat J , Angers S , Durocher D *et al* (2019) Identifying chemogenetic interactions from CRISPR screens with drugZ. Genome Med 11: 52 31439014 10.1186/s13073-019-0665-3 PMC6706933

[embj2022113190-bib-0014] Cong K , Cantor SB (2022) Exploiting replication gaps for cancer therapy. Mol Cell 82: 2363–2369 35568026 10.1016/j.molcel.2022.04.023 PMC9271608

[embj2022113190-bib-0015] Cong K , Peng M , Kousholt AN , Lee WTC , Lee S , Nayak S , Krais J , VanderVere‐Carozza PS , Pawelczak KS , Calvo J *et al* (2021) Replication gaps are a key determinant of PARP inhibitor synthetic lethality with BRCA deficiency. Mol Cell 81: 3128–3144 34216544 10.1016/j.molcel.2021.06.011 PMC9089372

[embj2022113190-bib-0016] Dev H , Chiang T‐WW , Lescale C , de Krijger I , Martin AG , Pilger D , Coates J , Sczaniecka‐Clift M , Wei W , Ostermaier M *et al* (2018) Shieldin complex promotes DNA end‐joining and counters homologous recombination in BRCA1‐null cells. Nat Cell Biol 20: 954–965 30022119 10.1038/s41556-018-0140-1 PMC6145444

[embj2022113190-bib-0017] DeWeirdt PC , Sangree AK , Hanna RE , Sanson KR , Hegde M , Strand C , Persky NS , Doench JG (2020) Genetic screens in isogenic mammalian cell lines without single cell cloning. Nat Commun 11: 752 32029722 10.1038/s41467-020-14620-6 PMC7005275

[embj2022113190-bib-0018] Dillehay LE , Thompson LH , Minkler JL , Carrano AV (1983) The relationship between sister‐chromatid exchange and perturbations in DNA replication in mutant EM9 and normal CHO cells. Mutat Res 109: 283–296 6843572 10.1016/0027-5107(83)90053-2

[embj2022113190-bib-0019] Dillehay LE , Thompson LH , Carrano AV (1984) DNA‐strand breaks associated with halogenated pyrimidine incorporation. Mutat Res 131: 129–136 6717468 10.1016/0167-8817(84)90052-x

[embj2022113190-bib-0020] Doench JG , Fusi N , Sullender M , Hegde M , Vaimberg EW , Donovan KF , Smith I , Tothova Z , Wilen C , Orchard R *et al* (2016) Optimized sgRNA design to maximize activity and minimize off‐target effects of CRISPR‐Cas9. Nat Biotechnol 34: 184–191 26780180 10.1038/nbt.3437 PMC4744125

[embj2022113190-bib-0021] El‐Khamisy SF , Saifi GM , Weinfeld M , Johansson F , Helleday T , Lupski JR , Caldecott KW (2005) Defective DNA single‐strand break repair in spinocerebellar ataxia with axonal neuropathy‐1. Nature 434: 108–113 15744309 10.1038/nature03314

[embj2022113190-bib-0022] Elstrodt F , Hollestelle A , Nagel JHA , Gorin M , Wasielewski M , van den Ouweland A , Merajver SD , Ethier SP , Schutte M (2006) BRCA1 mutation analysis of 41 human breast cancer cell lines reveals three new deleterious mutants. Cancer Res 66: 41–45 16397213 10.1158/0008-5472.CAN-05-2853

[embj2022113190-bib-0023] Ensminger M , Iloff L , Ebel C , Nikolova T , Kaina B , Löbrich M (2014) DNA breaks and chromosomal aberrations arise when replication meets base excision repairDNA and chromosome breakage at BER intermediates. J Cell Biol 206: 29–43 24982429 10.1083/jcb.201312078 PMC4085701

[embj2022113190-bib-0024] Farmer H , McCabe N , Lord CJ , Tutt ANJ , Johnson DA , Richardson TB , Santarosa M , Dillon KJ , Hickson I , Knights C *et al* (2005) Targeting the DNA repair defect in BRCA mutant cells as a therapeutic strategy. Nature 434: 917–921 15829967 10.1038/nature03445

[embj2022113190-bib-0025] Flores MJ , Bierne H , Ehrlich SD , Michel B (2001) Impairment of lagging strand synthesis triggers the formation of a RuvABC substrate at replication forks. EMBO J 20: 619–629 11157768 10.1093/emboj/20.3.619 PMC133471

[embj2022113190-bib-0026] Fong PC , Boss DS , Yap TA , Tutt A , Wu P , Mergui‐Roelvink M , Mortimer P , Swaisland H , Lau A , O'Connor MJ *et al* (2009) Inhibition of poly(ADP‐ribose) polymerase in tumors from BRCA mutation carriers. N Engl J Med 361: 123–134 19553641 10.1056/NEJMoa0900212

[embj2022113190-bib-0027] García‐Saura AG , Herzog LK , Dantuma NP , Schüler H (2021) MacroGreen, a simple tool for detection of ADP‐ribosylated proteins. Commun Biol 4: 919 34321589 10.1038/s42003-021-02439-w PMC8319303

[embj2022113190-bib-0028] Haapaniemi E , Botla S , Persson J , Schmierer B , Taipale J (2018) CRISPR‐Cas9 genome editing induces a p53‐mediated DNA damage response. Nat Med 24: 927–930 29892067 10.1038/s41591-018-0049-z

[embj2022113190-bib-0029] Haince J‐F , McDonald D , Rodrigue A , Déry U , Masson J‐Y , Hendzel MJ , Poirier GG (2008) PARP1‐dependent kinetics of recruitment of MRE11 and NBS1 proteins to multiple DNA damage sites. J Biol Chem 283: 1197–1208 18025084 10.1074/jbc.M706734200

[embj2022113190-bib-0030] Hanzlikova H , Caldecott KW (2019) Perspectives on PARPs in S Phase. Trends Genet 35: 412–422 31036342 10.1016/j.tig.2019.03.008

[embj2022113190-bib-0031] Hanzlikova H , Gittens W , Krejcikova K , Zeng Z , Caldecott KW (2017) Overlapping roles for PARP1 and PARP2 in the recruitment of endogenous XRCC1 and PNKP into oxidized chromatin. Nucleic Acids Res 45: 2546–2557 27965414 10.1093/nar/gkw1246 PMC5389470

[embj2022113190-bib-0032] Hanzlikova H , Kalasova I , Demin AA , Pennicott LE , Cihlarova Z , Caldecott KW (2018) The importance of poly(ADP‐ribose) polymerase as a sensor of unligated Okazaki fragments during DNA replication. Mol Cell 71: 319–331 29983321 10.1016/j.molcel.2018.06.004 PMC6060609

[embj2022113190-bib-0033] Hardeland U , Kunz C , Focke F , Szadkowski M , Schär P (2007) Cell cycle regulation as a mechanism for functional separation of the apparently redundant uracil DNA glycosylases TDG and UNG2. Nucleic Acids Res 35: 3859–3867 17526518 10.1093/nar/gkm337 PMC1920262

[embj2022113190-bib-0034] Hoch NC , Hanzlikova H , Rulten SL , Tetreault M , Komulainen E , Ju L , Hornyak P , Zeng Z , Gittens W , Rey SA *et al* (2017) XRCC1 mutation is associated with PARP1 hyperactivation and cerebellar ataxia. Nature 541: 87–91 28002403 10.1038/nature20790 PMC5218588

[embj2022113190-bib-0035] Hochegger H , Dejsuphong D , Fukushima T , Morrison C , Sonoda E , Schreiber V , Zhao GY , Saberi A , Masutani M , Adachi N *et al* (2006) Parp‐1 protects homologous recombination from interference by Ku and Ligase IV in vertebrate cells. EMBO J 25: 1305–1314 16498404 10.1038/sj.emboj.7601015 PMC1422167

[embj2022113190-bib-0036] Kouzminova EA , Kuzminov A (2012) Chromosome demise in the wake of ligase‐deficient replication. Mol Microbiol 84: 1079–1096 22582878 10.1111/j.1365-2958.2012.08076.x PMC3370103

[embj2022113190-bib-0037] Krokan HE , Otterlei M , Nilsen H , Kavli B , Skorpen F , Andersen S , Skjelbred C , Akbari M , Aas PA , Slupphaug G (2001) Properties and functions of human uracil‐DNA glycosylase from the UNG gene. Prog Nucleic Acid Res Mol Biol 68: 365–386 11554311 10.1016/s0079-6603(01)68112-1

[embj2022113190-bib-0038] Krokan HE , Drabløs F , Slupphaug G (2002) Uracil in DNA – occurrence, consequences and repair. Oncogene 21: 8935–8948 12483510 10.1038/sj.onc.1205996

[embj2022113190-bib-0039] Kuzminov A (2001) Single‐strand interruptions in replicating chromosomes cause double‐strand breaks. Proc Natl Acad Sci USA 98: 8241–8246 11459959 10.1073/pnas.131009198 PMC37427

[embj2022113190-bib-0040] Ledesma FC , Aguilera A (2006) Double‐strand breaks arising by replication through a nick are repaired by cohesin‐dependent sister‐chromatid exchange. EMBO Rep 7: 919–926 16888651 10.1038/sj.embor.7400774 PMC1559660

[embj2022113190-bib-0041] Longley DB , Harkin DP , Johnston PG (2003) 5‐Fluorouracil: mechanisms of action and clinical strategies. Nat Rev Cancer 3: 330–338 12724731 10.1038/nrc1074

[embj2022113190-bib-0042] Mol CD , Arvai AS , Slupphaug G , Kavli B , Alseth I , Krokan HE , Tainer JA (1995) Crystal structure and mutational analysis of human uracil‐DNA glycosylase: structural basis for specificity and catalysis. Cell 80: 869–878 7697717 10.1016/0092-8674(95)90290-2

[embj2022113190-bib-0043] Münzel M , Lischke U , Stathis D , Pfaffeneder T , Gnerlich FA , Deiml CA , Koch SC , Karaghiosoff K , Carell T (2011) Improved synthesis and mutagenicity of oligonucleotides containing 5‐hydroxymethylcytosine, 5‐formylcytosine and 5‐carboxylcytosine. Chemistry 17: 13782–13788 22069110 10.1002/chem.201102782

[embj2022113190-bib-0044] Nowak K , Rosenthal F , Karlberg T , Bütepage M , Thorsell A‐G , Dreier B , Grossmann J , Sobek J , Imhof R , Lüscher B *et al* (2020) Engineering Af1521 improves ADP‐ribose binding and identification of ADP‐ribosylated proteins. Nat Commun 11: 5199 33060572 10.1038/s41467-020-18981-w PMC7566600

[embj2022113190-bib-0045] Otterlei M , Warbrick E , Nagelhus TA , Haug T , Slupphaug G , Akbari M , Aas PA , Steinsbekk K , Bakke O , Krokan HE (1999) Post‐replicative base excision repair in replication foci. EMBO J 18: 3834–3844 10393198 10.1093/emboj/18.13.3834 PMC1171460

[embj2022113190-bib-0046] Panzarino NJ , Krais JJ , Cong K , Peng M , Mosqueda M , Nayak SU , Bond SM , Calvo JA , Doshi MB , Bere M *et al* (2021) Replication gaps underlie BRCA deficiency and therapy response. Cancer Res 81: 1388–1397 33184108 10.1158/0008-5472.CAN-20-1602 PMC8026497

[embj2022113190-bib-0047] Patel AG , Sarkaria JN , Kaufmann SH (2011) Nonhomologous end joining drives poly(ADP‐ribose) polymerase (PARP) inhibitor lethality in homologous recombination‐deficient cells. Proc Natl Acad Sci USA 108: 3406–3411 21300883 10.1073/pnas.1013715108 PMC3044391

[embj2022113190-bib-0048] Polo LM , Xu Y , Hornyak P , Garces F , Zeng Z , Hailstone R , Matthews SJ , Caldecott KW , Oliver AW , Pearl LH (2019) Efficient single‐strand break repair requires binding to both poly(ADP‐Ribose) and DNA by the central BRCT domain of XRCC1. Cell Rep 26: 573–581 30650352 10.1016/j.celrep.2018.12.082 PMC6334254

[embj2022113190-bib-0049] Riml C , Lusser A , Ennifar E , Micura R (2017) Synthesis, thermodynamic properties, and crystal structure of RNA oligonucleotides containing 5‐hydroxymethylcytosine. J Org Chem 82: 7939–7945 28707898 10.1021/acs.joc.7b01171

[embj2022113190-bib-0050] Rogakou EP , Pilch DR , Orr AH , Ivanova VS , Bonner WM (1998) DNA double‐stranded breaks induce histone H2AX phosphorylation on serine 139. J Biol Chem 273: 5858–5868 9488723 10.1074/jbc.273.10.5858

[embj2022113190-bib-0051] Rogakou EP , Boon C , Redon C , Bonner WM (1999) Megabase chromatin domains involved in DNA double‐strand breaks in vivo. J Cell Biol 146: 905–916 10477747 10.1083/jcb.146.5.905 PMC2169482

[embj2022113190-bib-0052] Saleh‐Gohari N , Bryant HE , Schultz N , Parker KM , Cassel TN , Helleday T (2005) Spontaneous homologous recombination is induced by collapsed replication forks that are caused by endogenous DNA single‐strand breaks. Mol Cell Biol 25: 7158–7169 16055725 10.1128/MCB.25.16.7158-7169.2005 PMC1190269

[embj2022113190-bib-0053] Scaramozzino N , Sanz G , Crance JM , Saparbaev M , Drillien R , Laval J , Kavli B , Garin D (2003) Characterisation of the substrate specificity of homogeneous vaccinia virus uracil‐DNA glycosylase. Nucleic Acids Res 31: 4950–4957 12907738 10.1093/nar/gkg672 PMC169932

[embj2022113190-bib-0054] Simoneau A , Xiong R , Zou L (2021) The trans cell cycle effects of PARP inhibitors underlie their selectivity toward BRCA1/2‐deficient cells. Genes Dev 35: 1271–1289 34385259 10.1101/gad.348479.121 PMC8415318

[embj2022113190-bib-0055] Somyajit K , Mishra A , Jameei A , Nagaraju G (2015) Enhanced non‐homologous end joining contributes toward synthetic lethality of pathological RAD51C mutants with poly (ADP‐ribose) polymerase. Carcinogenesis 36: 13–24 25292178 10.1093/carcin/bgu211

[embj2022113190-bib-0056] Sugimura K , Takebayashi S‐I , Taguchi H , Takeda S , Okumura K (2008) PARP‐1 ensures regulation of replication fork progression by homologous recombination on damaged DNA. J Cell Biol 183: 1203–1212 19103807 10.1083/jcb.200806068 PMC2606964

[embj2022113190-bib-0057] Thompson LH , Brookman KW , Jones NJ , Allen SA , Carrano AV (1990) Molecular cloning of the human XRCC1 gene, which corrects defective DNA strand break repair and sister chromatid exchange. Mol Cell Biol 10: 6160–6171 2247054 10.1128/mcb.10.12.6160-6171.1990 PMC362891

[embj2022113190-bib-0058] Tutt ANJ , Garber JE , Kaufman B , Viale G , Fumagalli D , Rastogi P , Gelber RD , de Azambuja E , Fielding A , Balmaña J *et al* (2021) Adjuvant olaparib for patients with BRCA1‐ or BRCA2‐mutated breast cancer. N Engl J Med 384: 2394–2405 34081848 10.1056/NEJMoa2105215 PMC9126186

[embj2022113190-bib-0059] Vaitsiankova A , Burdova K , Sobol M , Gautam A , Benada O , Hanzlikova H , Caldecott KW (2022) PARP inhibition impedes the maturation of nascent DNA strands during DNA replication. Nat Struct Mol Biol 29: 1–10 35332322 10.1038/s41594-022-00747-1 PMC9010290

[embj2022113190-bib-0060] Vrtis KB , Dewar JM , Chistol G , Wu RA , Graham TGW , Walter JC (2021) Single‐strand DNA breaks cause replisome disassembly. Mol Cell 81: 1309–1318 33484638 10.1016/j.molcel.2020.12.039 PMC7979477

[embj2022113190-bib-0061] Weiser BP , Rodriguez G , Cole PA , Stivers JT (2018) N‐terminal domain of human uracil DNA glycosylase (hUNG2) promotes targeting to uracil sites adjacent to ssDNA–dsDNA junctions. Nucleic Acids Res 46: 7169–7178 29917162 10.1093/nar/gky525 PMC6101581

[embj2022113190-bib-0062] Zdzienicka MZ , van der Schans GP , Natarajan AT , Thompson LH , Neuteboom I , Simons JW (1992) A Chinese hamster ovary cell mutant (EM‐C11) with sensitivity to simple alkylating agents and a very high level of sister chromatid exchanges. Mutagenesis 7: 265–269 1518409 10.1093/mutage/7.4.265

[embj2022113190-bib-0063] Zearfoss NR , Ryder SP (2012) End‐labeling oligonucleotides with chemical tags after synthesis. Methods Mol Biol 941: 181–193 23065562 10.1007/978-1-62703-113-4_14 PMC5026237

[embj2022113190-bib-0064] Zimmermann M , Murina O , Reijns MAM , Agathanggelou A , Challis R , Tarnauskaitė Ž , Muir M , Fluteau A , Aregger M , McEwan A *et al* (2018) CRISPR screens identify genomic ribonucleotides as a source of PARP‐trapping lesions. Nature 559: 285–289 29973717 10.1038/s41586-018-0291-z PMC6071917

