# Supplementary material for: Unrepaired base excision repair intermediates in template DNA strands trigger replication fork collapse and PARP inhibitor sensitivity
Source: EMBO J. 2023 Jul 26;42(18):e113190. doi: 10.15252/embj.2022113190 (PMC10505916; doi:10.15252/embj.2022113190)
Supplement: Supplementary file 1 — Expanded View Figures PDF [file EMBJ-42-e113190-s010.pdf]

Expanded View Figures

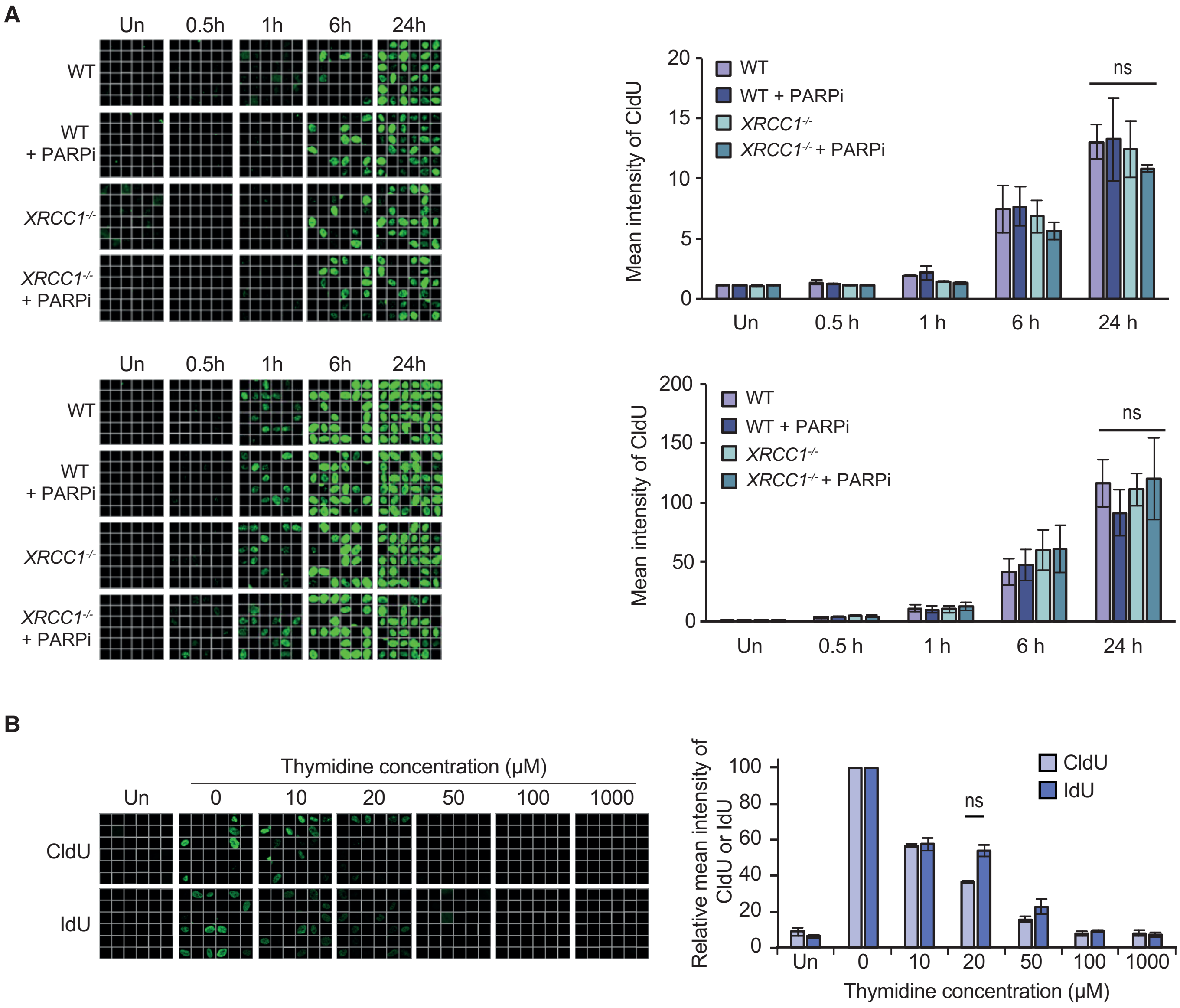

Figure EV1. CldU and IdU are incorporated at similar rates.

A Representative single-cell galleries from scanR high-content imaging (left) and quantification (right) of CldU in wild type (WT) and *XRCC1*$^{-/-}$ RPE-1 cells incubated with 1 μM (top), or 10 μM (bottom) CldU for the indicated times. CldU was detected following DNA denaturation by rat anti-BrdU (ab6326) antibody.

B Representative images (left) and quantification (right) as above of CldU or IdU in RPE-1 cells incubated for 1 h with 10 μM CldU or 10 μM IdU and the indicated concentrations of thymidine. CldU was detected as above and IdU was detected following DNA denaturation by mouse anti-BrdU (BD 347580) antibody. The width of each box (a single-cell) in the scanR image galleries is 10 μm.

Data information: (A, B) Data are the means (±SEM) of three independent biological repeats with > 1,000 cells (technical replicates) scored per sample per experiment, by scanR software. Statistical significance was assessed by (A) one-way ANOVA with Tukey's multiple comparisons test or (B) two-tailed paired *T*-test. ns, not significant; $P > 0.05$.

© 2023 The Authors

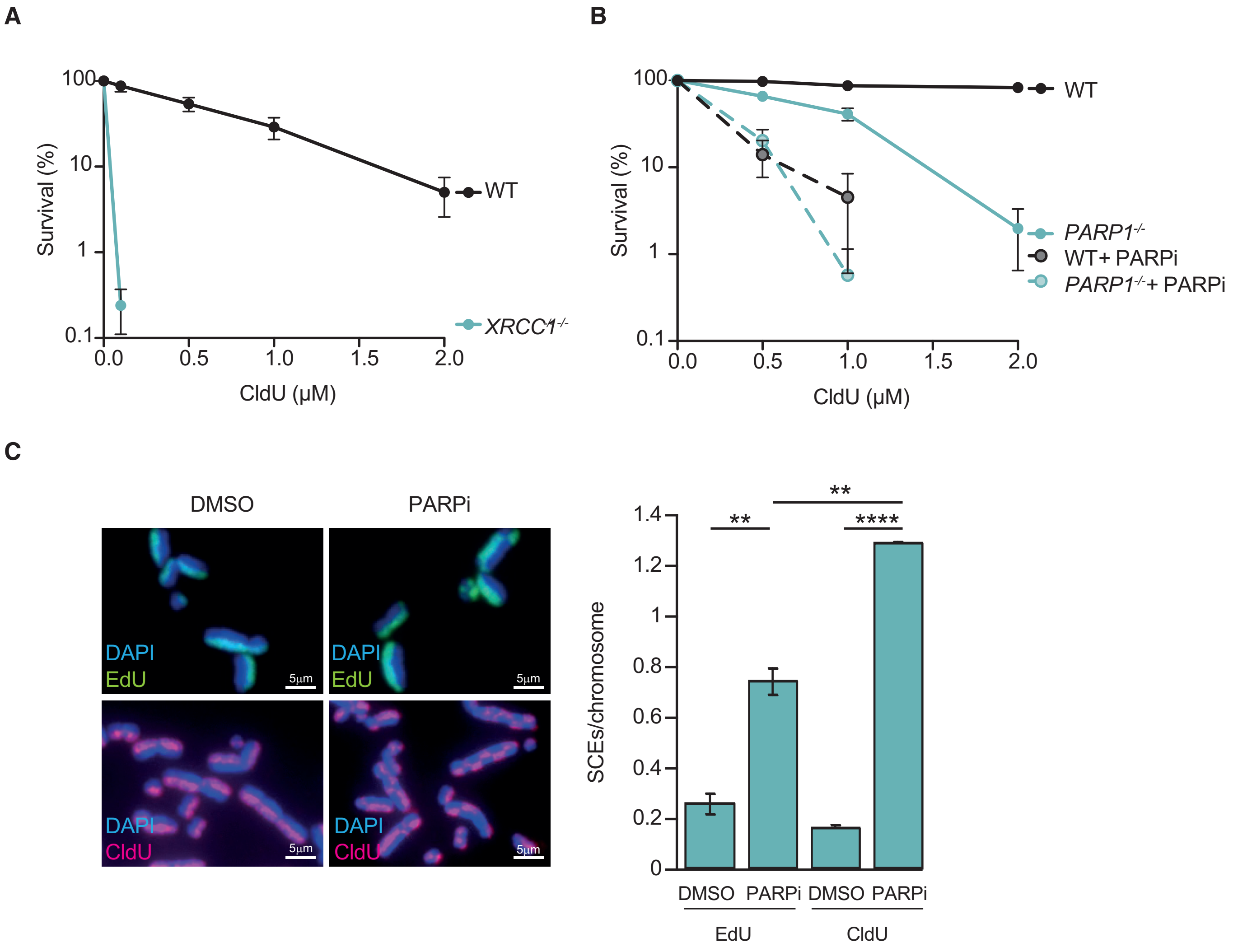

Figure EV2. Hypersensitivity to CldU in U2OS cells lacking XRCC1 or treated with PARP inhibitor.

A Clonogenic survival of wild type (WT) and *XRCC1*$^{-/-}$ U2OS cells following continuous treatment with the indicated concentrations of CldU.
B Clonogenic survival of WT and *PARP1*$^{-/-}$ U2OS cells following continuous treatment with the indicated concentrations of CldU in the absence or presence of 0.5 μM PARPi, as indicated.
C Representative images (left) and quantification (right) of sister chromatid exchanges detected by EdU click chemistry or anti-CldU immunofluorescence in U2OS cells treated with either EdU or CldU for the first 24 h and then with DMSO or 0.5 μM Ku58948 (PARPi) for the subsequent 24 h. Scale bars are 5 μm.

Data information: (A, B) Data are the mean of three independent biological repeats (±SEM). (C) Data are the means (±SEM) of two independent biological repeats, with 500 chromosomes (technical replicates) scored per sample per experiment. Statistical significance was determined by one-way ANOVA with Tukey's multiple comparisons test. $**P < 0.005$; $****P < 0.0001$.

© 2023 The Authors

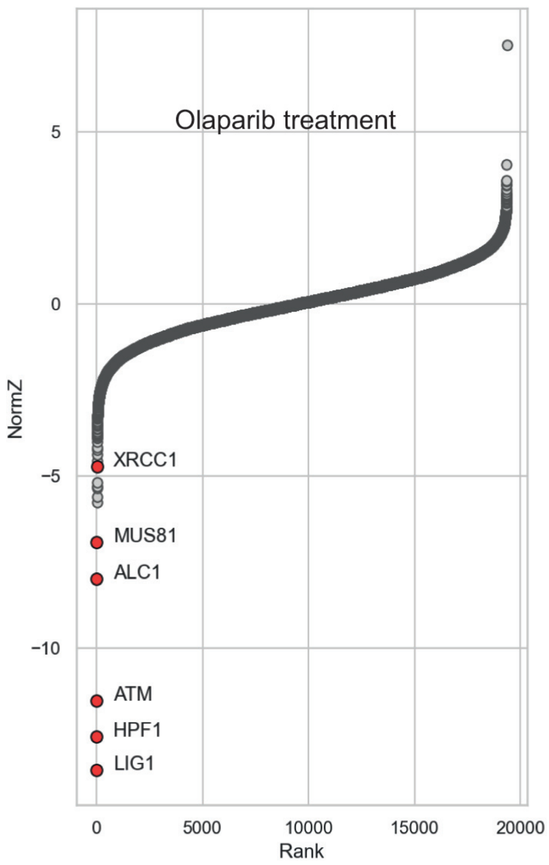

Figure EV3. CRISPR screen identifying human genes affecting sensitivity to olaparib alone.

Rank plot representation of CRISPR-Cas9 screen data. Results from cells treated with 0.5 μM olaparib alone are plotted and PARP1-related hits are highlighted.

 © 2023 The Authors

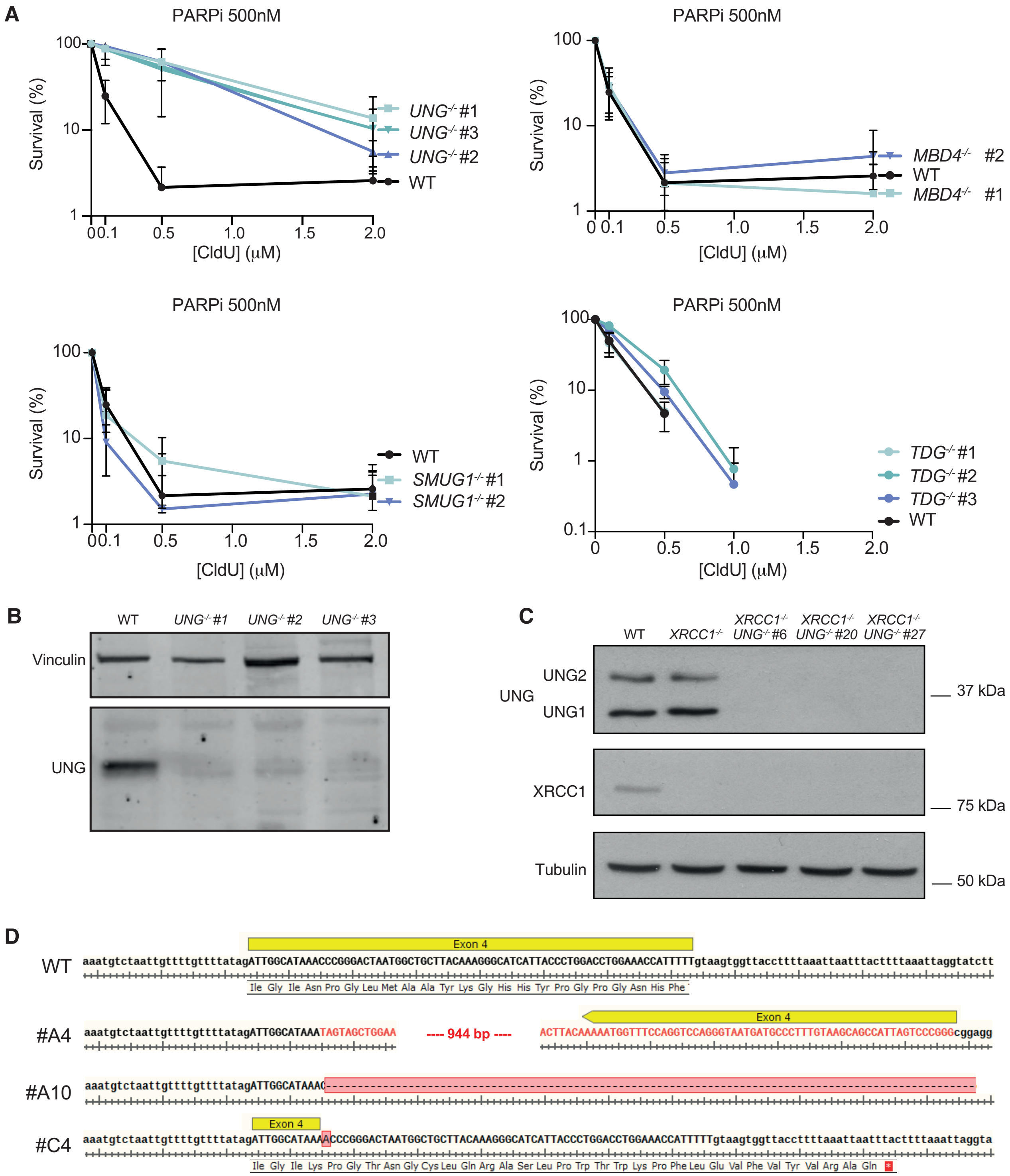

Figure EV4. Suppression of CldU sensitivity is specific to loss of UNG.

A Clonogenic survival of RPE-1 cell lines deleted of the indicted DNA glycosylases following continuous treatment with 0.5 μM olaparib and the indicated concentrations of CldU. Data are the means (±SD) of three independent biological repeats.

B Immunoblotting verification of UNG protein levels in *UNG*$^{-/-}$ RPE-1 cell lines generated by CRISPR-Cas9 gene editing.

C Immunoblotting verification of UNG and XRCC1 protein levels in WT, *XRCC1*$^{-/-}$, and *XRCC1*$^{-/-}$/*UNG*$^{-/-}$ (clones #6, #20, #27) RPE-1 cell lines.

D PCR amplification of genomic DNA surrounding the gRNA target sites followed by Sanger sequencing to confirm TDG gene editing in three individual RPE-1 clones (#A4, #A10, #C4). Note that these clones are labeled 1–3, in panel (A) above. Lower case letters represent introns and upper case represent exons. DNA base substitutions, insertions, and deletions are highlighted in red.

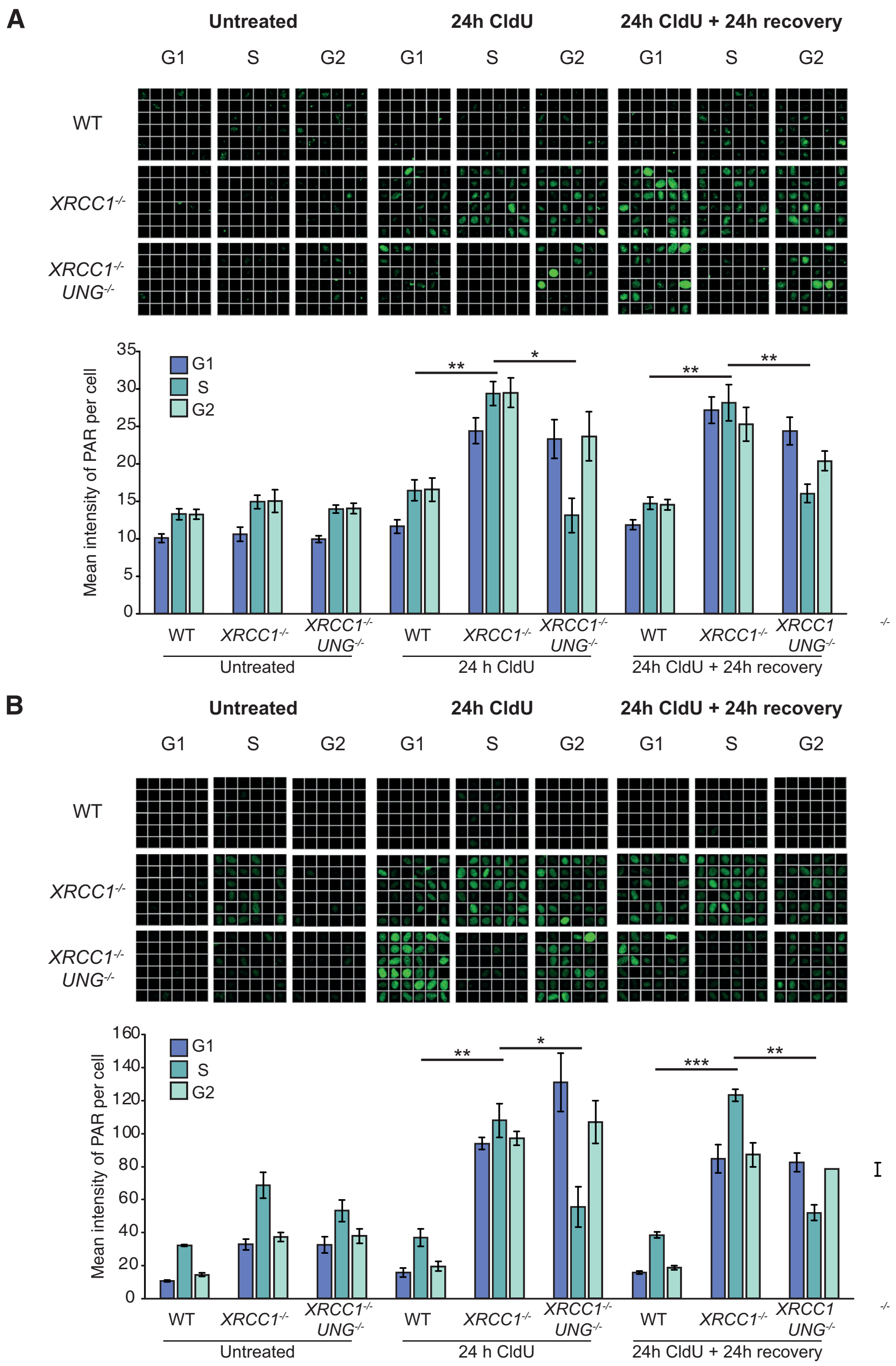

Figure EV5. UNG deletion suppresses CldU-induced SSB formation in SSBR-deficient cells.

A Representative single-cell galleries from scanR high-content imaging (top) and quantification (bottom) of poly(ADP-ribose) in WT, *XRCC1*$^{-/-}$ or *XRCC1*$^{-/-}$ *UNG*$^{-/-}$ RPE-1 cells following treatment with 10 μM CldU for 24 h followed by 24 h recovery where indicated. Cells were co-incubated for the final 30 min with EdU to label cells in S-phase, but PARGi was omitted. Note that ADP-ribosylation was detected in *XRCC1*$^{-/-}$ cells even in the absence of PARGi.

B Representative images (top) and quantification (bottom) as above of poly(ADP-ribose) in WT, *XRCC1*$^{-/-}$, and *XRCC1*$^{-/-}$ *UNG*$^{-/-}$ RPE-1 cells following treatment with 1 μM CldU for 24 h followed by 24 h recovery where indicated. Cells were co-incubated for the final 30 min with EdU to label cells in S-phase and PARG inhibitor (PARGi) to prevent poly(ADP-ribose) degradation. The width of each box (single-cell) in the scanR image galleries is 10 μm.

Data information: (A, B) Data are the mean (±SEM) of 4 (A) or 3 (B) independent biological repeats with > 1,000 cells (technical replicates) scored per sample per experiment by scanR software. Statistical significance was assessed by one-way ANOVA with Tukey's multiple comparisons test. *$P < 0.05$, **$P < 0.01$, ***$P < 0.001$.

 © 2023 The Authors

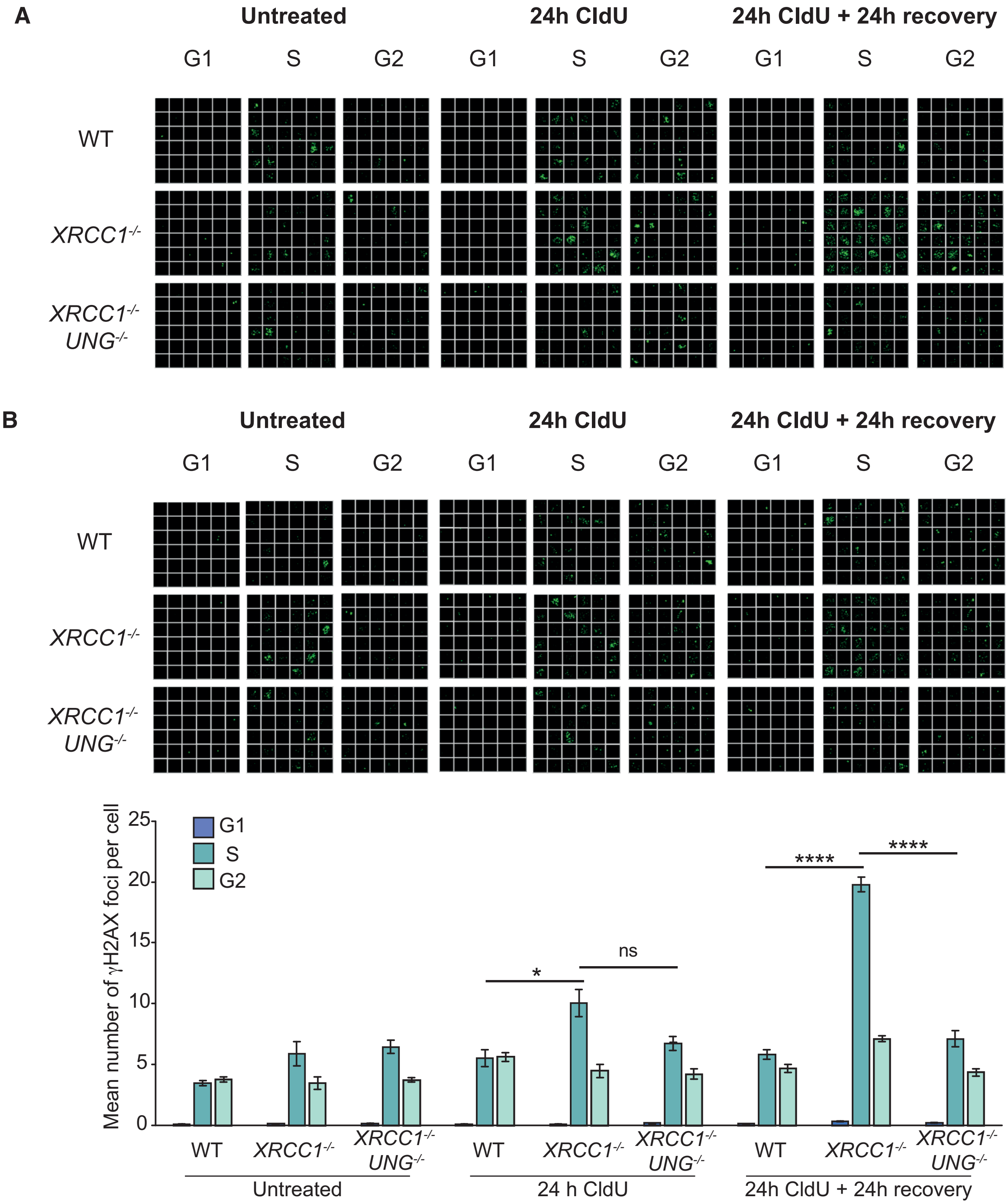

Figure EV6. UNG deletion suppresses CldU-induced DSB formation in SSBR-deficient cells.

A Representative single-cell galleries from scanR high-content imaging of H2AX-pSer139 (γH2AX) foci in WT, *XRCC1*$^{-/-}$ or *XRCC1*$^{-/-}$ *UNG*$^{-/-}$ RPE-1 cells following treatment with 10 μM CldU for 24 h followed by 24 h recovery where indicated. Cells were co-incubated for the final 30 min with EdU to label cells in S-phase.

B Representative images (top) and quantification (bottom) as above of H2AX-pSer139 (γH2AX) foci in WT, *XRCC1*$^{-/-}$ or *XRCC1*$^{-/-}$ *UNG*$^{-/-}$ RPE-1 cells following treatment with 1 μM CldU for 24 h followed by 24 h recovery, where indicated. Cells were co-incubated for the final 30 min with EdU to label cells in S-phase.

Data information: Data are the means of four independent biological repeats (±SEM) with > 1,000 cells (technical replicates) scored per sample per experiment by scanR software. Statistical significance was assessed by one-way ANOVA with Tukey's multiple comparisons test. *$P < 0.05$, ****$P < 0.0001$. The width of each box (a single-cell) in the scanR image galleries is 10 μm.

© 2023 The Authors
