# Supplementary material for: Unrepaired base excision repair intermediates in template DNA strands trigger replication fork collapse and PARP inhibitor sensitivity
Source: EMBO J. 2023 Jul 26;42(18):e113190. doi: 10.15252/embj.2022113190 (PMC10505916; doi:10.15252/embj.2022113190)
Supplement: Supplementary file 4 — Source Data for Figure 2 [file EMBJ-42-e113190-s007.zip › SD Figure 2/A/SD Figure 2A.pptx]

## Slide 1
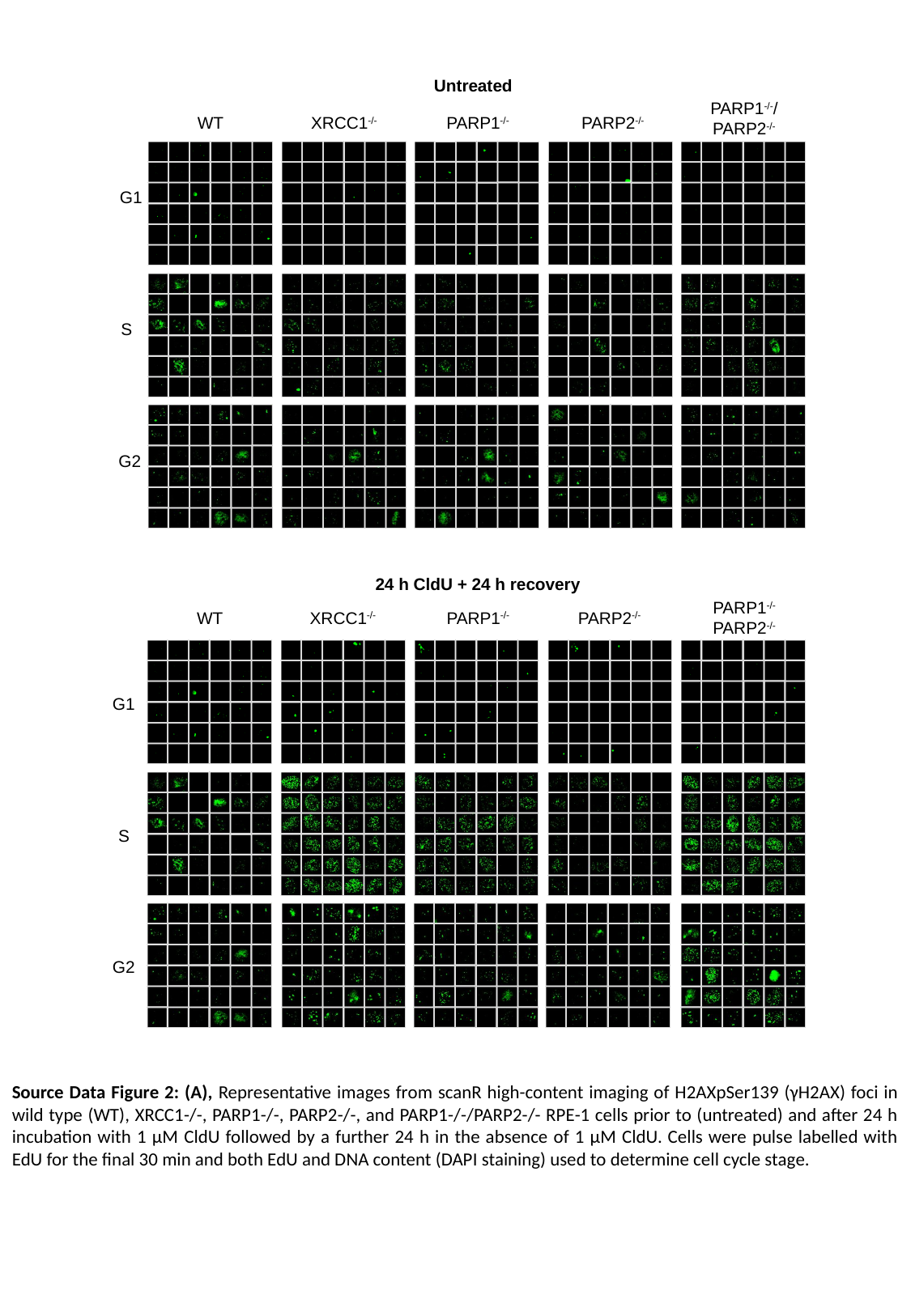

Untreated
PARP1-/-/PARP2-/-
WT
XRCC1-/-
PARP1-/-
PARP2-/-
G1
S
G2
24 h CldU + 24 h recovery
PARP1-/-
PARP2-/-
WT
XRCC1-/-
PARP1-/-
PARP2-/-
G1
S
G2
Source Data Figure 2: (A), Representative images from scanR high-content imaging of H2AXpSer139 (γH2AX) foci in wild type (WT), XRCC1-/-, PARP1-/-, PARP2-/-, and PARP1-/-/PARP2-/- RPE-1 cells prior to (untreated) and after 24 h incubation with 1 μM CldU followed by a further 24 h in the absence of 1 μM CldU. Cells were pulse labelled with EdU for the final 30 min and both EdU and DNA content (DAPI staining) used to determine cell cycle stage.
