# Supplementary material for: Unrepaired base excision repair intermediates in template DNA strands trigger replication fork collapse and PARP inhibitor sensitivity
Source: EMBO J. 2023 Jul 26;42(18):e113190. doi: 10.15252/embj.2022113190 (PMC10505916; doi:10.15252/embj.2022113190)
Supplement: Supplementary file 4 — Source Data for Figure 2 [file EMBJ-42-e113190-s007.zip › SD Figure 2/B/SD Figure 2B.pptx]

## Slide 1
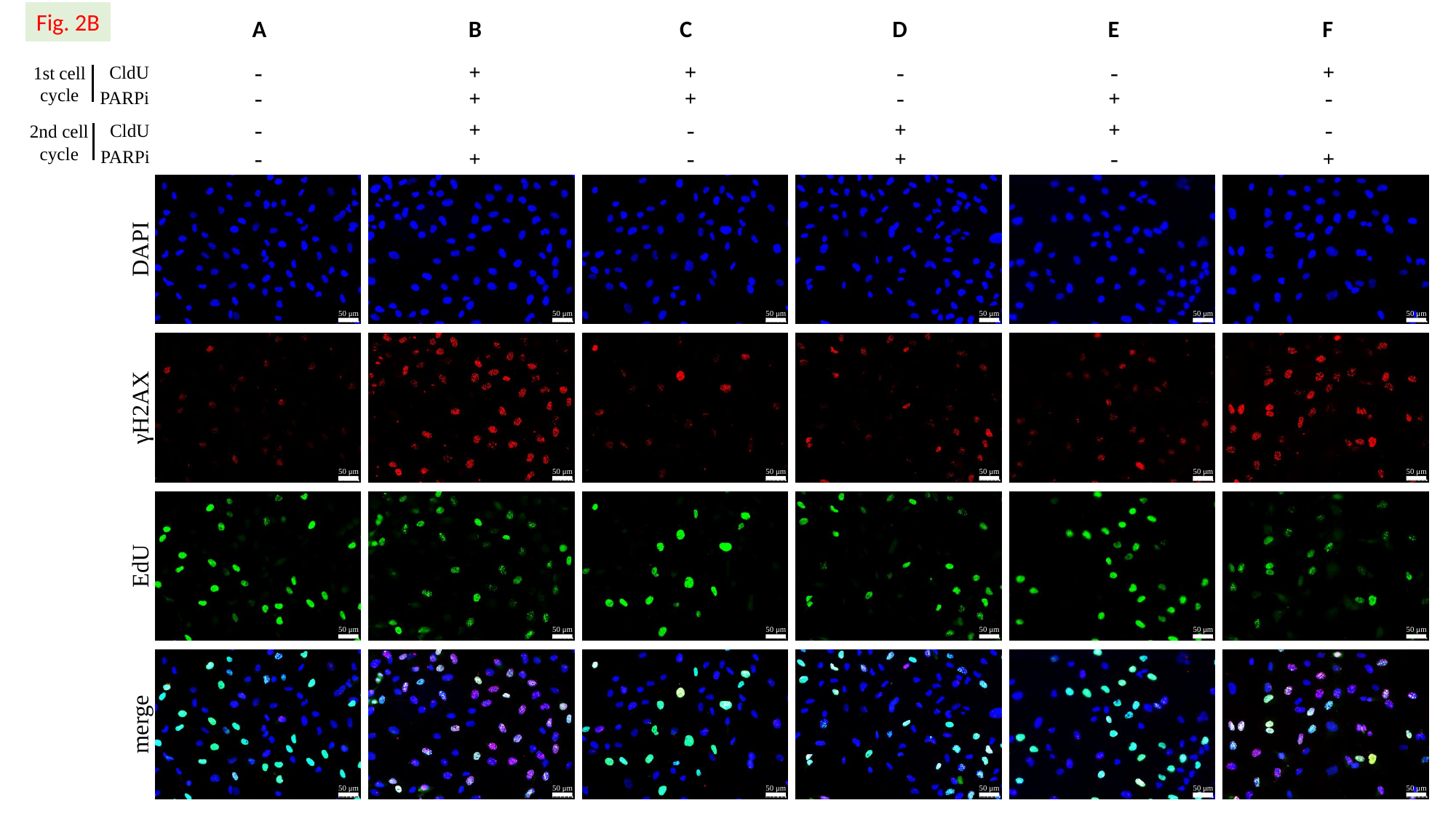

Fig. 2B
A
B
C
D
E
F
-
-
-
+
+
+
CldU
1st cell
cycle
-
-
-
+
+
+
PARPi
-
-
-
+
+
+
CldU
2nd cell
cycle
-
-
-
PARPi
+
+
+
DAPI
50 μm
50 μm
50 μm
50 μm
50 μm
50 μm
γH2AX
50 μm
50 μm
50 μm
50 μm
50 μm
50 μm
EdU
50 μm
50 μm
50 μm
50 μm
50 μm
50 μm
merge
50 μm
50 μm
50 μm
50 μm
50 μm
50 μm
