# Supplementary figures and images for: Unrepaired base excision repair intermediates in template DNA strands trigger replication fork collapse and PARP inhibitor sensitivity
Source: EMBO J. 2023 Jul 26;42(18):e113190. doi: 10.15252/embj.2022113190 (PMC10505916; doi:10.15252/embj.2022113190)

## Slide 1
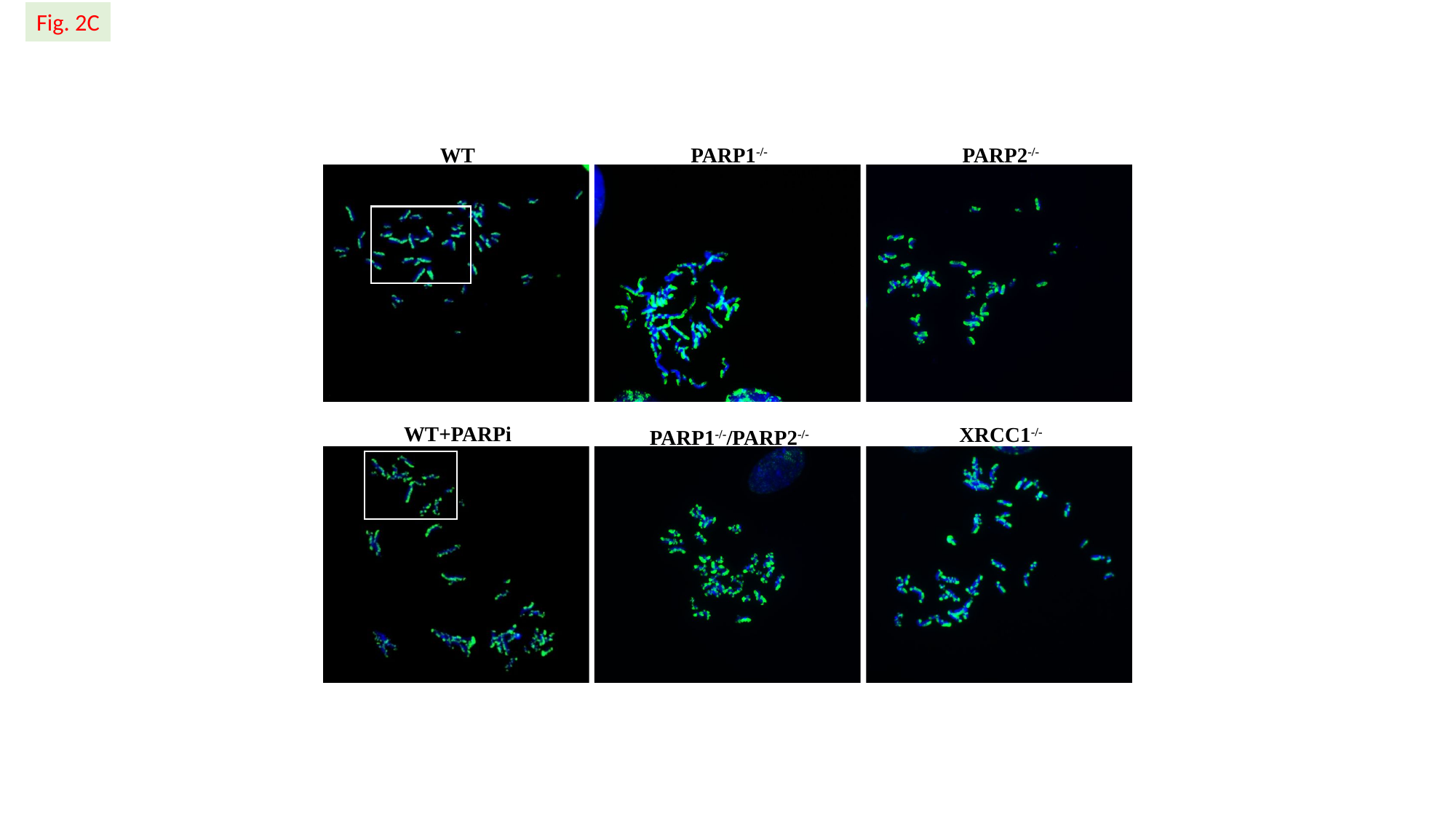

Fig. 2C
WT
PARP1-/-
PARP2-/-
WT+PARPi
XRCC1-/-
PARP1-/-/PARP2-/-

Supplement: Supplementary file 4 — Source Data for Figure 2 [file EMBJ-42-e113190-s007.zip › SD Figure 2/C/SD Figure 2C.pptx]

## Slide 1
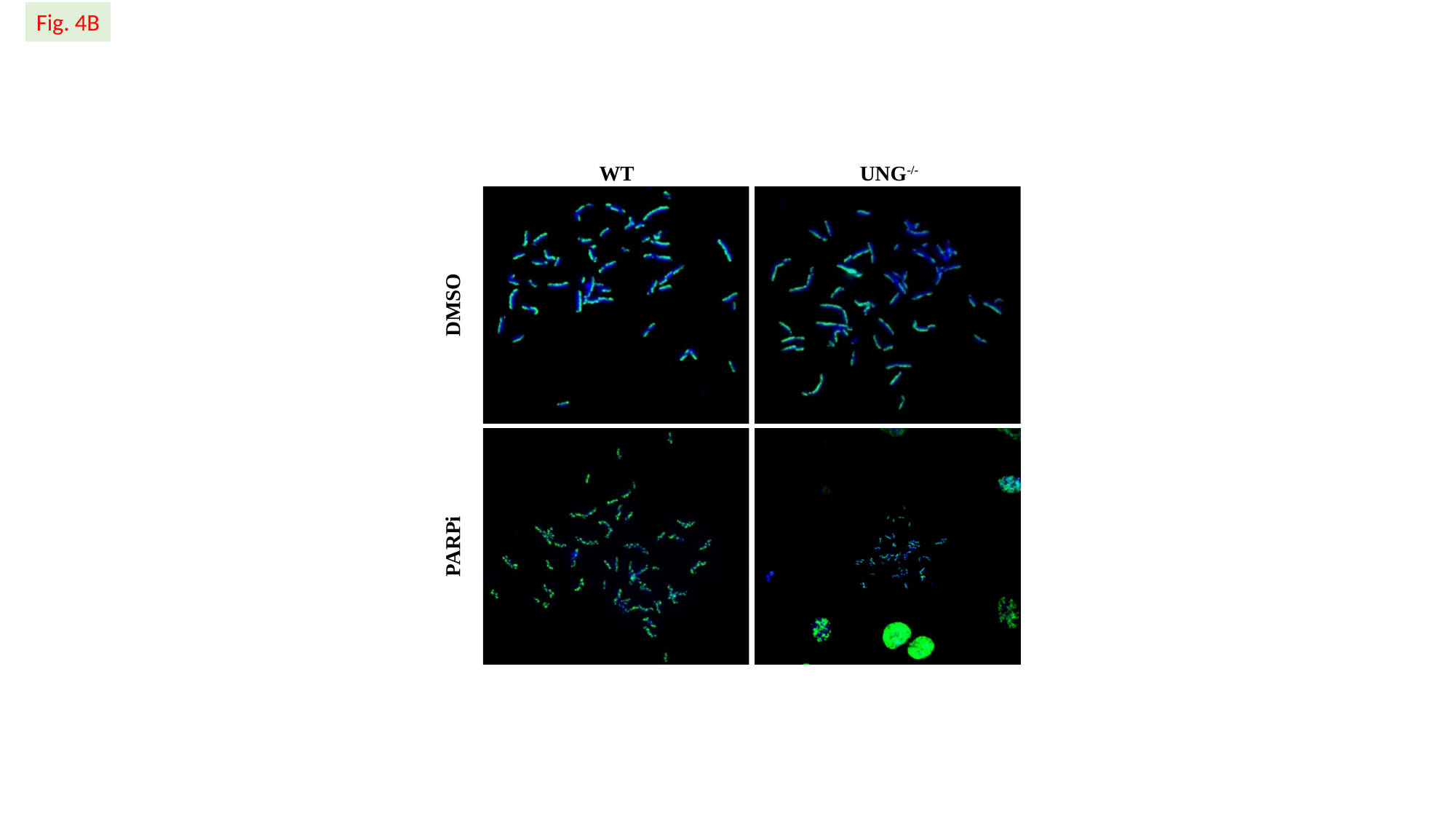

Fig. 4B
WT
UNG-/-
DMSO
PARPi

Supplement: Supplementary file 6 — Source Data for Figure 4 [file EMBJ-42-e113190-s004.zip › SD Figure 4/B/SD Figure 4B.pptx]
