# Supplementary material for: Unrepaired base excision repair intermediates in template DNA strands trigger replication fork collapse and PARP inhibitor sensitivity
Source: EMBO J. 2023 Jul 26;42(18):e113190. doi: 10.15252/embj.2022113190 (PMC10505916; doi:10.15252/embj.2022113190)
Supplement: Supplementary file 6 — Source Data for Figure 4 [file EMBJ-42-e113190-s004.zip › SD Figure 4/F/SD Figure 4F.pptx]

## Slide 1
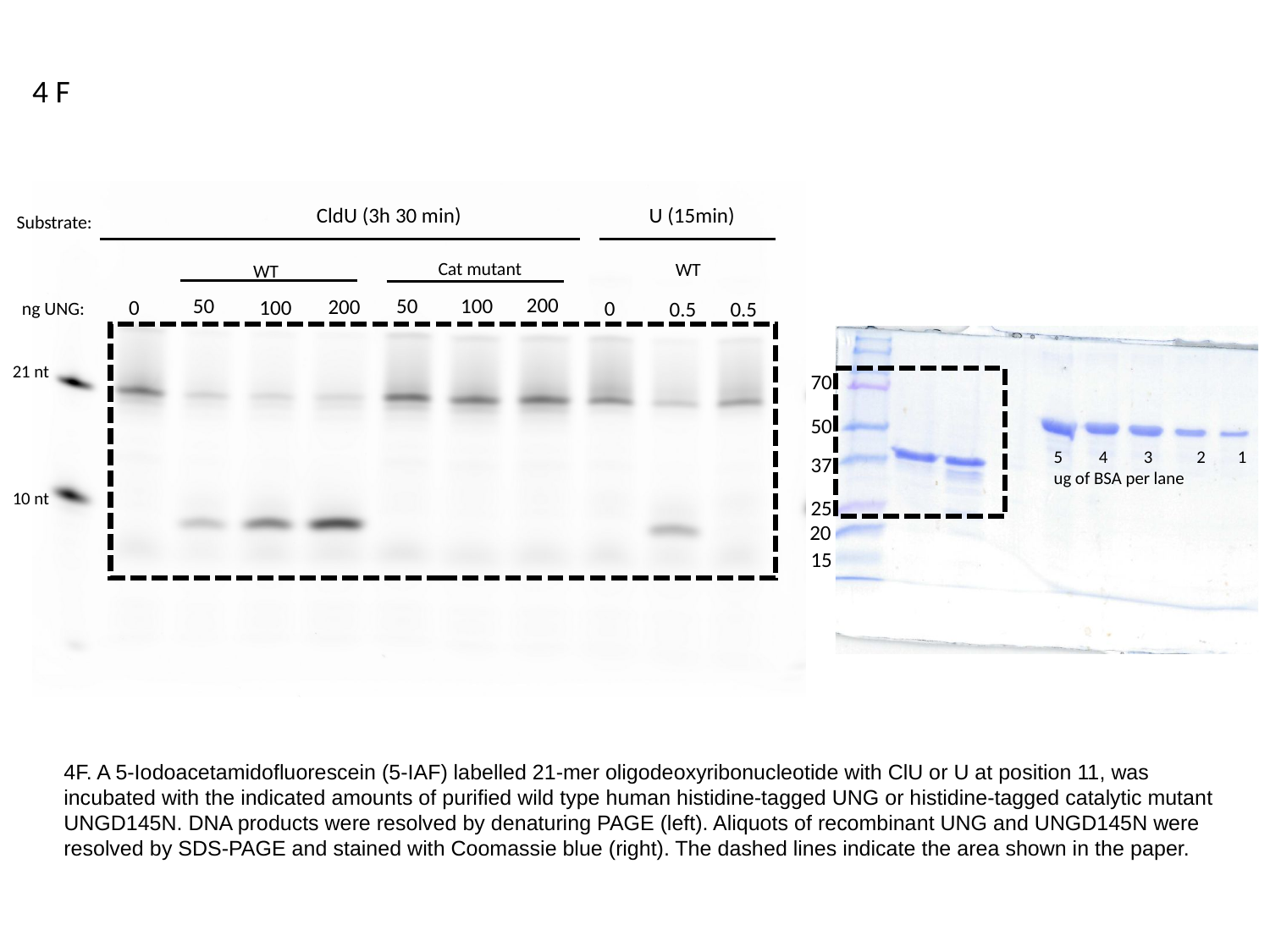

4 F
CldU (3h 30 min)
U (15min)
Substrate:
Cat mutant
WT
WT
200
50
50
100
200
0
100
0
0.5
0.5
ng UNG:
21 nt
10 nt
70
50
5 4 3 2 1
ug of BSA per lane
37
25
20
15
4F. A 5-Iodoacetamidofluorescein (5-IAF) labelled 21-mer oligodeoxyribonucleotide with ClU or U at position 11, was incubated with the indicated amounts of purified wild type human histidine-tagged UNG or histidine-tagged catalytic mutant UNGD145N. DNA products were resolved by denaturing PAGE (left). Aliquots of recombinant UNG and UNGD145N were resolved by SDS-PAGE and stained with Coomassie blue (right). The dashed lines indicate the area shown in the paper.
