# Supplementary material for: Unrepaired base excision repair intermediates in template DNA strands trigger replication fork collapse and PARP inhibitor sensitivity
Source: EMBO J. 2023 Jul 26;42(18):e113190. doi: 10.15252/embj.2022113190 (PMC10505916; doi:10.15252/embj.2022113190)
Supplement: Supplementary file 6 — Source Data for Figure 4 [file EMBJ-42-e113190-s004.zip › SD Figure 4/C/SD Figure 4C.pptx]

## Slide 1
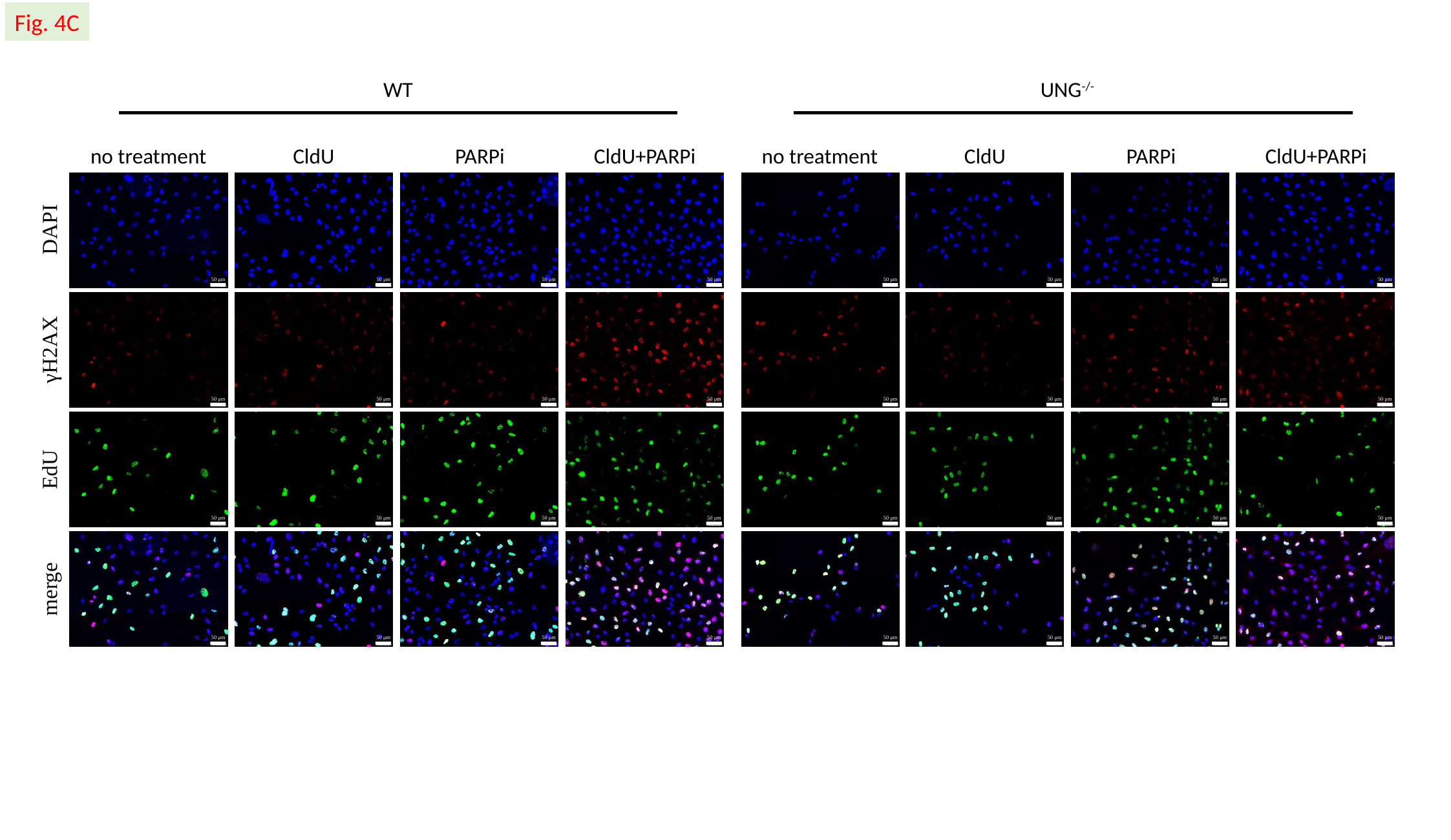

Fig. 4C
WT
UNG-/-
no treatment
CldU
PARPi
CldU+PARPi
no treatment
CldU
PARPi
CldU+PARPi
DAPI
50 μm
50 μm
50 μm
50 μm
50 μm
50 μm
50 μm
50 μm
γH2AX
50 μm
50 μm
50 μm
50 μm
50 μm
50 μm
50 μm
50 μm
EdU
50 μm
50 μm
50 μm
50 μm
50 μm
50 μm
50 μm
50 μm
merge
50 μm
50 μm
50 μm
50 μm
50 μm
50 μm
50 μm
50 μm
