# Supplementary material for: Unrepaired base excision repair intermediates in template DNA strands trigger replication fork collapse and PARP inhibitor sensitivity
Source: EMBO J. 2023 Jul 26;42(18):e113190. doi: 10.15252/embj.2022113190 (PMC10505916; doi:10.15252/embj.2022113190)
Supplement: Supplementary file 7 — Source Data for Figure 5 [file EMBJ-42-e113190-s002.zip › SD Figure 5/A/SD Figure 5A.pptx]

## Slide 1
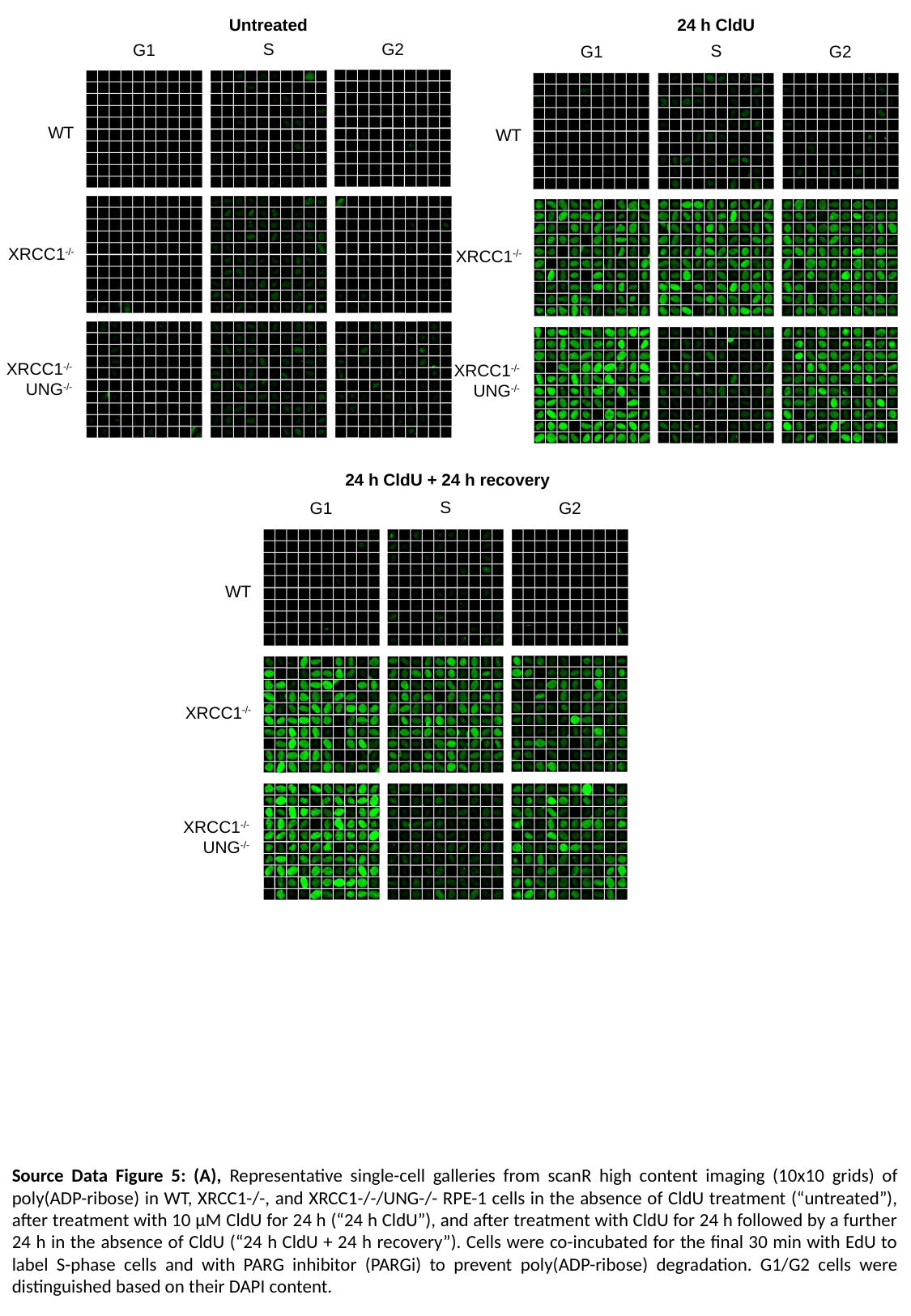

Untreated
24 h CldU
S
G2
G1
WT
XRCC1-/-
XRCC1-/-
UNG-/-
S
G2
G1
WT
XRCC1-/-
XRCC1-/-
UNG-/-
24 h CldU + 24 h recovery
S
G2
G1
WT
XRCC1-/-
XRCC1-/-
UNG-/-
Source Data Figure 5: (A), Representative single-cell galleries from scanR high content imaging (10x10 grids) of poly(ADP-ribose) in WT, XRCC1-/-, and XRCC1-/-/UNG-/- RPE-1 cells in the absence of CldU treatment (“untreated”), after treatment with 10 µM CldU for 24 h (“24 h CldU”), and after treatment with CldU for 24 h followed by a further 24 h in the absence of CldU (“24 h CldU + 24 h recovery”). Cells were co-incubated for the final 30 min with EdU to label S-phase cells and with PARG inhibitor (PARGi) to prevent poly(ADP-ribose) degradation. G1/G2 cells were distinguished based on their DAPI content.
