# Supplementary material for: Unrepaired base excision repair intermediates in template DNA strands trigger replication fork collapse and PARP inhibitor sensitivity
Source: EMBO J. 2023 Jul 26;42(18):e113190. doi: 10.15252/embj.2022113190 (PMC10505916; doi:10.15252/embj.2022113190)
Supplement: Supplementary file 10 [file EMBJ-42-e113190-s009.pdf]

THE EMBO JOURNAL

Unrepaired Base Excision Repair Intermediates in Template DNA Strands Trigger Replication Fork Collapse and PARP Inhibitor Sensitivity

Almudena Serrano-Benitez, Sophie Wells, Lylah Drummond-Clarke, Lilian Russo, John Thomas, Giovanna Leal, Mark Farrow, James Edgerton, Shankar Balsubramanian, Ming Yang, Christian Frezza, Amit Gautam, Jan Brazina, Kamila Burdova, Nicolas Hoch, Stephen Jackson, and Keith Caldecott
DOI: 10.15252/embj.2022113190

Corresponding author(s): Keith Caldecott (k.w.caldecott@sussex.ac.uk) , Stephen Jackson (spj13@cam.ac.uk), Nicolas Hoch (nicolas@iq.usp.br)

Review Timeline:

Submission Date:	2nd Dec 22
Editorial Decision:	16th Jan 23
Revision Received:	17th May 23
Accepted:	7th Jul 23

Editor: Hartmut Vodermaier

Transaction Report:

(Note: With the exception of the correction of typographical or spelling errors that could be a source of ambiguity, letters and reports are not edited. Depending on transfer agreements, referee reports obtained elsewhere may or may not be included in this compilation. Referee reports are anonymous unless the Referee chooses to sign their reports.)

Prof. Keith W Caldecott
University of Sussex
Genome Damage and Stability Centre
Science Park Road
Falmer
Brighton, Sussex BN1 9RQ
United Kingdom

16th Jan 2023

Re: EMBOJ-2022-113190
Base Excision Repair Intermediates in Template DNA Trigger Replication Fork Collapse

Dear Keith,

Thank you again for submitting your study on the impact of BER intermediates in replication template strands to The EMBO Journal. I have now heard back from three expert referees, whose reports you will find copied below. As you will see, all referees acknowledge the decisiveness of the presented results, and a majority also considers the findings and conclusions of the work topical and broadly significant. In this light, we would be happy to pursue an adequately revised version of the manuscript further for EMBO Journal publication. Key points in this regard would be to confirm the action of purified UNG on CldU-containing DNA (ref 1), extending the analysis of BRCA1-deficient cells to another, distinct HR-deficient background (ref 3), and to validate a few of the key results obtained with PARPi also with PARP deletion (ref 2). In addition, to address referee 2's conceptual/novelty concerns, referee 1 recommended to better describe that the topic of PARPi sensitivity has remained controversial and to add more discussion of the conflicting literature in this area.

Since it is our policy to consider only a single round of major revision and therefore important to fully answer to all comments at the time of resubmission, I would invite you to get back to me with a tentative response letter/revision plan already during the early stages of the revision work. On the basis of this response, we could then further discuss the revision requirements and how to best address the key concerns e.g. via a follow-up video call. I should add that we could also offer extension of the default three-months revision period if needed, with our 'scooping protection' (meaning that competing work appearing elsewhere in the meantime will not affect our considerations of your study) remaining of course valid also throughout this extension.

Detailed information on preparing, formatting and uploading a revised manuscript can be found below and in our Guide to Authors. Thank you again for the opportunity to consider this work for The EMBO Journal, and I look forward to hearing from you in due time.

With kind regards,

Hartmut

Hartmut Vodermaier, PhD
Senior Editor, The EMBO Journal
h.vodermaier@embojournal.org

*** PLEASE NOTE: All revised manuscript are subject to initial checks for completeness and adherence to our formatting guidelines. Revisions may be returned to the authors and delayed in their editorial re-evaluation if they fail to comply to the following requirements (see also our Guide to Authors for further information):

1) Every manuscript requires a Data Availability section (even if only stating that no deposited datasets are included). Primary datasets or computer code produced in the current study have to be deposited in appropriate public repositories prior to resubmission, and reviewer access details provided in case that public access is not yet allowed. Further information: embopress.org/page/journal/14602075/authorguide#dataavailability

2) Each figure legend must specify
- size of the scale bars that are mandatory for all micrograph panels
- the statistical test used to generate error bars and P-values
- the type error bars (e.g., S.E.M., S.D.)
- the number (n) and nature (biological or technical replicate) of independent experiments underlying each data point

- Figures may not include error bars for experiments with n<3; scatter plots showing individual data points should be used instead.

3) Revised manuscript text (including main tables, and figure legends for main and EV figures) has to be submitted as editable text file (e.g., .docx format). We encourage highlighting of changes (e.g., via text color) for the referees' reference.

4) Each main and each Expanded View (EV) figure should be uploaded as individual production-quality files (preferably in .eps, .tif, .jpg formats). For suggestions on figure preparation/layout, please refer to our Figure Preparation Guidelines: http://bit.ly/EMBOPressFigurePreparationGuideline

5) Point-by-point response letters should include the original referee comments in full together with your detailed responses to them (and to specific editor requests if applicable), and also be uploaded as editable (e.g., .docx) text files.

6) Please complete our Author Checklist, and make sure that information entered into the checklist is also reflected in the manuscript; the checklist will be available to readers as part of the Review Process File. A download link is found at the top of our Guide to Authors: embopress.org/page/journal/14602075/authorguide

7) All authors listed as (co-)corresponding need to deposit, in their respective author profiles in our submission system, a unique ORCiD identifier linked to their name. Please see our Guide to Authors for detailed instructions.

8) Please note that supplementary information at EMBO Press has been superseded by the 'Expanded View' for inclusion of additional figures, tables, movies or datasets; with up to five EV Figures being typeset and directly accessible in the HTML version of the article. For details and guidance, please refer to: embopress.org/page/journal/14602075/authorguide#expandedview

9) Digital image enhancement is acceptable practice, as long as it accurately represents the original data and conforms to community standards. If a figure has been subjected to significant electronic manipulation, this must be clearly noted in the figure legend and/or the 'Materials and Methods' section. The editors reserve the right to request original versions of figures and the original images that were used to assemble the figure. Finally, we generally encourage uploading of numerical as well as gel/blot image source data; for details see: embopress.org/page/journal/14602075/authorguide#sourcedata

At EMBO Press, we ask authors to provide source data for the main manuscript figures. Our source data coordinator will contact you to discuss which figure panels we would need source data for and will also provide you with helpful tips on how to upload and organize the files.

Further information is available in our Guide For Authors: https://www.embopress.org/page/journal/14602075/authorguide

In the interest of ensuring the conceptual advance provided by the work, we recommend submitting a revision within 3 months (16th Apr 2023). Please discuss the revision progress ahead of this time with the editor if you require more time to complete the revisions. Use the link below to submit your revision:

Link Not Available

Referee #1:

This paper from Caldecott and colleagues reports that CldU incorporation during S phase sensitizes cells to the absence of base excision repair (XRCC1 deletion or PARPi addition), but only if cells are allowed to enter a second S phase. The sensitivity is rescued in cells lacking the uracil DNA glycosylase UNG. They show that UNG is able to process CldU in the first and second S phase, arguing that only its presence in template strands is toxic. Consistent with this idea, fork collapse only occurs in the second S phase after CldU incorporation. Finally, they show that BRCA-deficient cells are more sensitive than WT cells to the combination of CldU and PARPi. Their data support a model in which BER intermediates are toxic during replication only when these are generated in the template strand, likely due to fork breakage when the replisome hits such a lesion. The resulting collapsed for would have to be repaired by HR. They also point out that CldU could synergize with PARPi in the treatment of HR-deficient tumors.

The effects shown in this manuscript are dramatic and clear cut, and given the current interest in the interplay of BER, PARPi, HR, and DNA replication, the message is timely and important. To corroborate the model that UNG exerts its effects by directly removing CldU from the genome, the authors should show that purified UNG is able to act on CldU-containing DNA. In addition,

some aspects of the data are surprising, and the authors should do a better job of explaining them based on current knowledge (see points 1-3).

Specific points

1. It is surprising that in Figure 2, the level of gH2AX staining is the same when PARPi was added only during the second cell cycle as when it is added to the first and second cell cycles. In the former scenario, why are many or most of the CldU lesions incorporated during the first cell cycle not repaired by BER before cells go through the second S phase? This is especially puzzling given that UNG is proposed to function in S phase behind the fork, via coupling to PCNA.

2. In Figure 5A, UNG deletion abolishes ssDNA formation in S phase but not in G1 or G2. This implies that other glycosylases can process CldU in other phases of the cell cycle. Why don't these other glycosylases act in S phase? Is their activity suppressed at this stage of the cell cycle? More importantly, if these other glycosylases can act in the G2 or next G1 after CldU has been incorporated in one S phase, why do the resulting breaks, which should not be repaired in the absence of Xrcc1, not cause breaks and toxicity in the next S phase, even in UNG-/- cells? In other words, how does the loss of UNG rescue sensitivity to CldU (Figure 4)?

3. Please comment on why Parp1/Parp2 deficient cells are more sensitive to CldU than Xrcc1-deficient cells (Figure 1E)

4. In Figure 5A, how do the authors idenitfy G1, S, and G2 cells?

Referee #2:

The PARPs activated during the DNA damage response, PARP1 and PARP2, have many different functions. While the best characterized is their role in the repair of DNA single strand breaks, there is growing evidence for functions during DNA replication. This situation is further complicated by the fact that PARP inhibitors have different effects than genetic deletion due, at least in part part, because of the ability of some inhibitors to trap PARP1/2 on chromatin. In this study the author have looked at the consequences of SSBs in nascent DNA behind the fork or in the template DNA ahead of the fork. The experimental strategy using a halogenated nucleoside that is a substrate for uracil DNA glycosylase is clever and the results presented pervade compelling evidence that SSBs in the template DNA ahead of the fork give rise to DSBs. That being said, the results primarily confirm existing paradigms and so while definitive, are somewhat lacking novelly and significance. Another weakness of the study is that beyond the survival curves shown in Fig.1, all the results use a PARP inhibitor rather than genetic inactivation. Studies with single and double PARP knockout strains should be carried out to determine the contribution of PARP trapping to DSB and SCE formation.

Referee #3:

In this manuscript, the authors show that a deficiency in single-strand break repair (SSBR) induced by loss of XRCC1 or inhibition of PARP1 causes an exquisite sensitivity to 5-chloro-2'-deoxyuridine (CldU), a thymidine analog widely used for DNA replication studies. Using a CRISPR screen, they discover that this exquisite sensitivity is due to the S phase activity of the uracil DNA glycosylase (UNG). Moreover, they show that the CldU incorporated into the replicating DNA becomes cytotoxic only in the following round of the cell cycle, when is located on the template DNA. Collectively, these studies support a model where a base excision repair (BER) intermediate created by UNG activity in the template DNA strand head of replication forks triggers fork breakage upon collision of the replication fork with this intermediate. Overall, the study is well-executed and has several important implications for the DNA repair and replication fields. First, it shows that BER intermediates have a different impact on replication fork stability depending on whether they are located on the nascent DNA behind replication forks or on the template DNA ahead of replication forks. Second, the authors' observation that CldU is toxic under certain conditions is relevant to the use of this thymidine analog for replication studies. Third, these studies open the tantalizing scenario that CldU could be used in cancer therapy to improve the clinical efficacy of PARPi in BRCA-deficient tumors. However, there are a few concerns that the authors should address to strengthen their findings and support their conclusions.

1. The experiments of Figure 1 nicely show that human cells lacking XRCC1 or treated with Olaparib have an exquisite sensitivity to CldU. These experiments were performed using the same concentrations for all the halogens (CldU, BrdU, FdU, and IdU). Are the IC50s different for these halogens? Is CldU more stable that BrdU, FdU, or IdU in solution? Does CldU permeate the cell membrane better than the other halogens? These questions should be considered and at least discussed in the manuscript as they are important for a proper interpretation of the results.

2. The authors should explain the lack of the CldU only sample in their CRISPR screening.

3. Figure 4B. The authors should measure whether chlorodeoxyuridine incorporation varies between wild-type and XRCC1 -/- or PARPi cells as this is important to show that the increased sensitivity of XRCC1-/- and PARPi cells to CldU is not related to increased CldU incorporation.

4. Figure 7. The authors should extend the analysis to BRCA2-deficient cells to test whether results obtained in BRCA1-deficient cells can be recapitulated in other HR-deficient backgrounds.

Dear Hartmut,

Please find enclosed our response to the referees. We believe we have now addressed all of the referees' queries, and we thank yourself and the referees for the opportunity to improve our manuscript.

Best wishes,
Keith

Referee #1:

This paper from Caldecott and colleagues reports that CldU incorporation during S phase sensitizes cells to the absence of base excision repair (XRCC1 deletion or PARPi addition), but only if cells are allowed to enter a second S phase. The sensitivity is rescued in cells lacking the uracil DNA glycosylase UNG. They show that UNG is able to process CldU in the first and second S phase, arguing that only its presence in template strands is toxic. Consistent with this idea, fork collapse only occurs in the second S phase after CldU incorporation. Finally, they show that BRCA-deficient cells are more sensitive than WT cells to the combination of CldU and PARPi. Their data support a model in which BER intermediates are toxic during replication only when these are generated in the template strand, likely due to fork breakage when the replisome hits such a lesion. The resulting collapsed for would have to be repaired by HR. They also point out that CldU could synergize with PARPi in the treatment of HR-deficient tumors.

The effects shown in this manuscript are dramatic and clear cut, and given the current interest in the interplay of BER, PARPi, HR, and DNA replication, the message is timely and important. To corroborate the model that UNG exerts its effects by directly removing CldU from the genome, the authors should show that purified UNG is able to act on CldU-containing DNA. In addition, some aspects of the data are surprising, and the authors should do a better job of explaining them based on current knowledge (see points 1-3).

We thank this referee for his/her positive and constructive comments and suggestions, which we address in detail below. As suggested, we have now determined that UNG does indeed remove ClU from DNA (please see new Fig.4F). We have also added relevant explanations for points 1-3 below and in the text.

Specific points

1. It is surprising that in Figure 2, the level of gH2AX staining is the same when PARPi was added only during the second cell cycle as when it is added to the first and second cell cycles. In the former scenario, why are many or most of the CldU lesions incorporated during the first cell cycle not repaired by BER before cells go through the second S phase? This is especially puzzling given that UNG is proposed to function in S phase behind the fork, via coupling to PCNA.

This is a great question, and we apologise for the lack of clarity in our original submission. The vast majority of CldU incorporated in the first cell cycle persists into the second cell cycle, presumably because it saturates the BER capability of the cell. For example, we found that 30 hours after incubation with 1uM CldU, the level of CldU in the genome of WT cells was only 25% less than that present in *UNG*$^{-/-}$ cells (Fig.4E). Thus, 75% of incorporated CldU persists at least 30 hours after it is incorporated, even in UNG-proficient cells. This makes sense, in that CldU would not be such an efficient S phase label if it was rapidly removed. The persistence of incorporated CldU may also reflect the fact that while we find that UNG can indeed remove ClU from DNA, it does so at much lower efficiency (>100-fold) than its canonical substrate, uracil (new Fig.4F). This is now discussed as requested, at the end of the second paragraph of the revised Discussion.

2. In Figure 5A, UNG deletion abolishes ssDNA formation in S phase but not in G1 or G2. This implies that other glycosylases can process CldU in other phases of the cell cycle. Why don't these other glycosylases act in S phase? Is their activity suppressed at this stage of the cell cycle? More importantly, if these other glycosylases can act in the G2 or next G1 after CldU has been incorporated in one S phase, why do the resulting breaks, which should not be repaired in the absence of Xrcc1, not cause breaks and toxicity in the next S phase, even in UNG-/- cells? In other words, how does the loss of UNG rescue sensitivity to CldU (Figure 4)?

Another great question. Indeed, UNG activity is restricted to S phase, and is replaced in G1/G2 by TDG, which is itself degraded in S-phase (Hardeland et al Nucleic Acids Research, 2007, Vol. 35, No. 11 3859–3867). UNG and TDG thus provide non-redundant uracil DNA glycosylase activity across the cell cycle (Hardeland et al Nucleic Acids Research, 2007, Vol. 35, No. 11 3859–3867). We do not yet know if TDG also excises ClU but it seems likely that it does, and that this is the source of the CldU-induced PARP activity in G1/G2. We believe that the reason that SSBs arising in G1 are not sufficient to cause toxicity in the next S phase is because they are repaired too quickly, even in the absence of XRCC1. SSB repair is slowed ~3-6-fold in the absence of XRCC1, typically increasing the half-life of SSBs to 30-90 min (Zdzienicka et al 1992; Thompson et al 1990). Although slower than in wild type cells, this is still sufficient time for most cells in G1/G2 to repair SSBs prior to entry into

the next S phase. We have now discussed this as requested (first paragraph on Page 10/11 of the revised manuscript).

3. Please comment on why Parp1/Parp2 deficient cells are more sensitive to CldU than Xrcc1-deficient cells (Figure 1E).

This we believe reflects the requirement for PARP activity for functions other than BER, such as the regulation of DNA replication fork reversal and/or processing at SSBs. We have now discussed this as requested (first paragraph of the revised Discussion).

4. In Figure 5A, how do the authors identify G1, S, and G2 cells?
S phase cells were distinguished by EdU pulse labelling and G1/G2 cells by measuring DAPI content, using scanR high-content imaging/microscopy.

Referee #2:

The PARPs activated during the DNA damage response, PARP1 and PARP2, have many different functions. While the best characterized is their role in the repair of DNA single strand breaks, there is growing evidence for functions during DNA replication. This situation is further complicated by the fact that PARP inhibitors have different effects than genetic deletion due, at least in part part, because of the ability of some inhibitors to trap PARP1/2 on chromatin. In this study the author have looked at the consequences of SSBs in nascent DNA behind the fork or in the template DNA ahead of the fork. The experimental strategy using a halogenated nucleoside that is a substrate for uracil DNA glycosylase is clever and the results presented pervade compelling evidence that SSBs in the template DNA ahead of the fork give rise to DSBs. That being said, the results primarily confirm existing paradigms and so while definitive, are somewhat lacking novelly and significance. Another weakness of the study is that beyond the survival curves shown in Fig.1, all the results use a PARP inhibitor rather than genetic inactivation. Studies with single and double PARP knockout strains should be carried out to determine the contribution of PARP trapping to DSB and SCE formation.

We thank the referee for his/her generally supportive comments. We believe that our paper is significant in that it discounts the emerging popular idea that SSBs/ssDNA gaps induced in human cells behind DNA replication forks are intrinsically cytotoxic (at least those arising during BER). Rather, our data argue that it is the induction or persistence of SSBs ahead of approaching forks that are cytotoxic. We agree that the latter is not a new concept but nonetheless this question has become contentious in recent years, and we believe our work elegantly demonstrates that this concept is true in human cells.

We have now added the requested experiments using PARP1 single/double mutants (new Fig.2A & 2C), which show that similar to cells treated with PARP inhibitor, PARP1 KO and PARP1/PARP2 KO cells exhibit elevated DSBs and SCEs following incubation with CldU. These data complement our clonogenic survival experiments and show that PARP trapping is not necessary for the phenotypes reported here.

Referee #3:

In this manuscript, the authors show that a deficiency in single-strand break repair (SSBR) induced by loss of XRCC1 or inhibition of PARP1 causes an exquisite sensitivity to 5-chloro-2'-deoxyuridine (CldU), a thymidine analog widely used for DNA replication studies. Using a CRISPR screen, they discover that this exquisite sensitivity is due to the S phase activity of the uracil DNA glycosylase (UNG). Moreover, they show that the CldU incorporated into the replicating DNA becomes cytotoxic only in the following round of the cell cycle, when is located on the template DNA. Collectively, these studies support a model where a base excision repair (BER) intermediate created by UNG activity in the template DNA strand head of replication forks triggers fork breakage upon collision of the replication fork with this intermediate. Overall, the study is well-executed and has several important implications for the DNA repair and replication fields. First, it shows that BER intermediates have a different impact on replication fork stability depending on whether they are located on the nascent DNA behind replication forks or on the template DNA ahead of replication forks. Second, the authors' observation that CldU is toxic under certain conditions is relevant to the use of this thymidine analog for replication studies. Third, these studies open the tantalizing scenario that CldU could be used in cancer therapy to improve the clinical efficacy of PARPi in BRCA-deficient tumors. However, there are a few concerns that the authors should address to strengthen their findings and support their conclusions.

We thank this referee for his/her positive and constructive comments and suggestions, which we address in detail below.

1. The experiments of Figure 1 nicely show that human cells lacking XRCC1 or treated with Olaparib have an exquisite sensitivity to CldU. These experiments were performed using the same concentrations for all the halogens (CldU, BrdU, FdU, and IdU). Are the IC50s different for these halogens? Is CldU more stable that BrdU, FdU, or IdU in solution? Does CldU permeate the cell membrane better than the other halogens? These

questions should be considered and at least discussed in the manuscript as they are important for a proper interpretation of the results.

These are an excellent set of related questions, which we felt warranted addressing experimentally. To do this, we chose CldU and IdU as examples of toxic and non-toxic thymidine analogues, respectively. We then compared exogenous thymidine for its ability to compete with and suppress the incorporation of CldU and IdU (measured by quantitative immunofluorescence). The rationale here is that if CldU uptake/incorporation is greater than that of IdU (accounting for its toxicity), then the concentration of exogenous thymidine needed to reduce CldU incorporation (e.g. by 50%) should be greater than required to reduce IdU incorporation by the same amount. However, we found that CldU and IdU incorporation were suppressed to similar extents over a range of exogenous thymidine concentrations. For example, the incorporation of both CldU and IdU (when present at 10μM) was reduced by ~50% if thymidine was also present at 10μM, suggesting that all three analogues are absorbed and incorporated with similar efficiencies. These data are now present in the new Supplementary Fig.1B (EV1B).

2. The authors should explain the lack of the CldU only sample in their CRISPR screening. We thank the reviewer for pointing this out. Wild type (WT) cells are extraordinarily sensitive to CldU treatment when combined with olaparib, but we would have needed extremely high concentrations of CldU alone to kill 20% of the cells. We were therefore concerned that the toxicity to CldU alone in WT cells at such high concentrations may involve “off target” effects unrelated to DNA repair, such as nucleotide imbalances resulting from thymidylate synthase inhibition. In light of these considerations, we decided not to include CldU-only in our CRISPR screening.

3. Figure 4B. The authors should measure whether chlorodeoxyuridine incorporation varies between wild-type and XRCC1 -/- or PARPi cells as this is important to show that the increased sensitivity of XRCC1-/- and PARPi cells to CldU is not related to increased CldU incorporation.

This is a very good point. We have now added these data in Supplementary Fig.1A (EV1A). We show that the incorporation of CldU is similar in WT and XRCC1-/- cells, irrespective of whether PARPi is present or not.

4. Figure 7. The authors should extend the analysis to BRCA2-deficient cells to test whether results obtained in BRCA1-deficient cells can be recapitulated in other HR-deficient backgrounds.

Another great idea. We have now added these data, which are similar to those obtained with BRCA1-defective cells, to the new Fig.7 of our revised manuscript (panels E-J). Moreover, in BRCA2-defective cells, we also detect hypersensitivity to CldU alone.

Prof. Keith W Caldecott
University of Sussex
Genome Damage and Stability Centre
Science Park Road
Falmer
Brighton, Sussex BN1 9RQ
United Kingdom

7th Jul 2023

Re: EMBOJ-2022-113190R
Unrepaired Base Excision Repair Intermediates in Template DNA Strands Trigger Replication Fork Collapse and PARP Inhibitor Sensitivity

Dear Keith,

Thank you for submitting your final revised manuscript for our consideration. I am pleased to inform you that in light of the positive re-review (copied below for you information), we have now accepted it for publication in The EMBO Journal.

Your article will be processed for publication in The EMBO Journal by EMBO Press and Wiley, who will contact you with further information regarding production/publication procedures and license requirements. You will also be provided with page proofs after copy-editing and typesetting of main manuscript and expanded view figure files.

Should you be planning a Press Release on your article, please get in contact with embojournal@wiley.com as early as possible, in order to coordinate publication and release dates.

Thank you again for this contribution to The EMBO Journal and congratulations on a successful publication! Please consider us again in the future for your most exciting work.

Yours sincerely,

Hartmut

Hartmut Vodermaier, PhD
Senior Editor, The EMBO Journal
h.vodermaier@embojournal.org

Referee #3:

The authors did an excellent job addressing my previous concerns. The manuscript is significantly improved and can be accepted without further changes.

EMBO Press Author Checklist

Corresponding Author Name: Keith W Caldecott
Journal Submitted to: EMBO J
Manuscript Number: EMBOJ-2022-113190

USEFUL LINKS FOR COMPLETING THIS FORM
The EMBO Journal - Author Guidelines
EMBO Reports - Author Guidelines
Molecular Systems Biology - Author Guidelines
EMBO Molecular Medicine - Author Guidelines

Reporting Checklist for Life Science Articles (updated January
This checklist is adapted from Materials Design Analysis Reporting (MDAR) Checklist for Authors. MDAR establishes a minimum set of requirements in transparent reporting in the life sciences (see Statement of Task: 10.31222/osf.io/9sm4x). Please follow the journal's guidelines in preparing your
Please note that a copy of this checklist will be published alongside your article.

Abridged guidelines for figures
1. Data
The data shown in figures should satisfy the following conditions:
- the data were obtained and processed according to the field's best practice and are presented to reflect the results of the experiments in an accurate and unbiased manner.
- ideally, figure panels should include only measurements that are directly comparable to each other and obtained with the same assay.
- plots include clearly labeled error bars for independent experiments and sample sizes. Unless justified, error bars should not be shown for technical
- if n<5, the individual data points from each experiment should be plotted. Any statistical test employed should be justified.
- Source Data should be included to report the data underlying figures according to the guidelines set out in the authorship guidelines on Data

2. Captions
Each figure caption should contain the following information, for each panel where they are relevant:
- a specification of the experimental system investigated (eg cell line, species name).
- the assay(s) and method(s) used to carry out the reported observations and measurements.
- an explicit mention of the biological and chemical entity(ies) that are being measured.
- an explicit mention of the biological and chemical entity(ies) that are altered/varied/perturbed in a controlled manner.
- the exact sample size (n) for each experimental group/condition, given as a number, not a range;
- a description of the sample collection allowing the reader to understand whether the samples represent technical or biological replicates (including how many animals, litters, cultures, etc.).
- a statement of how many times the experiment shown was independently replicated in the laboratory.
- definitions of statistical methods and measures:
  - common tests, such as t-test (please specify whether paired vs. unpaired), simple χ^2 tests, Wilcoxon and Mann-Whitney tests, can be unambiguously identified by name only, but more complex techniques should be described in the methods section;
  - are tests one-sided or two-sided?
  - are there adjustments for multiple comparisons?
  - exact statistical test results, e.g., P values = x but not P values < x;
  - definition of 'center values' as median or average;
  - definition of error bars as s.d. or s.e.m.

Please complete ALL of the questions below.
Select "Not Applicable" only when the requested information is not relevant for your study.

Materials

Newly Created Materials	Information included in the manuscript?	In which section is the information available? (Reagents and Tools Table, Materials and Methods, Figures, Data Availability Section)
New materials and reagents need to be available; do any restrictions apply?	Yes	All reagents/tools are decribed in materials and methods. All reagents are available, as stated in the Data Availability section.

Antibodies	Information included in the manuscript?	In which section is the information available? (Reagents and Tools Table, Materials and Methods, Figures, Data Availability Section)
For **antibodies** provide the following information:<br>- Commercial antibodies: RRID (if possible) or supplier name, catalogue number and or/clone number<br>- Non-commercial: RRID or citation	Yes	All antibodies and their catalogue numbers and sources are described in the Materials and Methods section

DNA and RNA sequences	Information included in the manuscript?	In which section is the information available? (Reagents and Tools Table, Materials and Methods, Figures, Data Availability Section)
**Short novel DNA or RNA including primers, probes**: provide the sequences.	Yes	All oligo/primer sequences are provided in the materials and methods section.

Cell materials	Information included in the manuscript?	In which section is the information available? (Reagents and Tools Table, Materials and Methods, Figures, Data Availability Section)
**Cell lines:** Provide species information, strain. Provide accession number in repository **OR** supplier name, catalog number, clone number, and/**OR** RRID.	Yes	All cell line details are included in the Materials and Methods section
**Primary cultures:** Provide species, strain, sex of origin, genetic modification status.	Not Applicable	
Report if the cell lines were recently **authenticated** (e.g., by STR profiling) and tested for mycoplasma contamination.	Yes	Cell lines are checked regularly for mycoplasma contamination and authenticated by PCR analysis. This is stated in the materials and Methods section.

Experimental animals	Information included in the manuscript?	In which section is the information available? (Reagents and Tools Table, Materials and Methods, Figures, Data Availability Section)
**Laboratory animals or Model organisms:** Provide species, strain, sex, age, genetic modification status. Provide accession number in repository **OR** supplier name, catalog number, clone number, **OR** RRID.	Not Applicable	
**Animal observed in or captured from the field:** Provide species, sex, and age where possible.	Not Applicable	
Please detail **housing and husbandry conditions**.	Not Applicable	

Plants and microbes	Information included in the manuscript?	In which section is the information available? (Reagents and Tools Table, Materials and Methods, Figures, Data Availability Section)
**Plants:** provide species and strain, ecotype and cultivar where relevant, unique accession number if available, and source (including location for collected wild specimens).	Not Applicable	
**Microbes:** provide species and strain, unique accession number if available, and source.	Not Applicable	

Human research participants	Information included in the manuscript?	In which section is the information available? (Reagents and Tools Table, Materials and Methods, Figures, Data Availability Section)
If collected and within the bounds of privacy constraints report on age, sex and gender or ethnicity for all study participants.	Not Applicable	

Core facilities	Information included in the manuscript?	In which section is the information available? (Reagents and Tools Table, Materials and Methods, Figures, Data Availability Section)
If your work benefited from core facilities, was their service mentioned in the acknowledgments section?	Not Applicable	These are stated in the Acknowledgements

Design

Study protocol	Information included in the manuscript?	In which section is the information available? (Reagents and Tools Table, Materials and Methods, Figures, Data Availability Section)
If study protocol has been **pre-registered, provide DOI in the manuscript**. For clinical trials, provide the trial registration number **OR** cite DOI.	Not Applicable	
Report the **clinical trial registration number** (at ClinicalTrials.gov or equivalent), where applicable.	Not Applicable	

Laboratory protocol	Information included in the manuscript?	In which section is the information available? (Reagents and Tools Table, Materials and Methods, Figures, Data Availability Section)
Provide DOI OR other citation details if **external detailed step-by-step protocols** are available.	Not Applicable	

Experimental study design and statistics	Information included in the manuscript?	In which section is the information available? (Reagents and Tools Table, Materials and Methods, Figures, Data Availability Section)
Include a statement about **sample size** estimate even if no statistical methods were used.	Yes	All sample sizes etc are stated in the figure legends
Were any steps taken to minimize the effects of subjective bias when allocating animals/samples to treatment (e.g. **randomization procedure**)? If yes, have they been described?	Yes	All quantification by scanR/software is conducted on cells/samples selected in a randomised/unbiased manner. This is stated in the materials and methods section
Include a statement about **blinding** even if no blinding was done.	Yes	All quantification by scanR is effectively conducted 'blind' because the cells are chosen and scored by the automated software.
Describe **inclusion/exclusion criteria** if samples or animals were excluded from the analysis. Were the criteria pre-established?<br><br>If sample or data points were omitted from analysis, report if this was due to attrition or intentional exclusion and provide justification.	Not Applicable	
For every figure, are **statistical tests** justified as appropriate? Do the data meet the assumptions of the tests (e.g., normal distribution)? Describe any methods used to assess it. Is there an estimate of variation within each group of data? Is the variance similar between the groups that are being statistically compared?	Yes	Statistical tests etc are stated in the legends

Sample definition and in-laboratory replication	Information included in the manuscript?	In which section is the information available? (Reagents and Tools Table, Materials and Methods, Figures, Data Availability Section)
In the figure legends: state number of times the experiment was **replicated** in laboratory.	Yes	these are stated in the legends
In the figure legends: define whether data describe **technical or biological replicates**.	Yes	we state in the legends the number of independent experimental (biological) repeats, and where relevant the number of technical repeats (e.g. the number of cells scored per sample per experiment)

Ethics

Ethics	Information included in the manuscript?	In which section is the information available? (Reagents and Tools Table, Materials and Methods, Figures, Data Availability Section)
Studies involving **human participants**: State details of **authority granting ethics approval** (IRB or equivalent committee(s), provide reference number for approval.	Not Applicable	
Studies involving **human participants**: Include a statement confirming that **informed consent** was obtained from all subjects and that the experiments conformed to the principles set out in the WMA Declaration of Helsinki and the Department of Health and Human Services Belmont Report.	Not Applicable	
Studies involving **human participants:** For publication of **patient photos**, include a statement confirming that consent to publish was obtained.	Not Applicable	
Studies involving experimental **animals**: State details of **authority granting ethics approval** (IRB or equivalent committee(s), provide reference number for approval. Include a statement of compliance with ethical regulations.	Not Applicable	
Studies involving **specimen and field samples:** State if relevant **permits** obtained, provide details of authority approving study; if none were required, explain why.	Not Applicable	

Dual Use Research of Concern (DURC)	Information included in the manuscript?	In which section is the information available? (Reagents and Tools Table, Materials and Methods, Figures, Data Availability Section)
Could your study fall under dual use research restrictions? Please check biosecurity documents and list of **select agents and toxins** (CDC): https://www.selectagents.gov/sat/list.htm	Not Applicable	
If you used a select agent, is the security level of the lab appropriate and reported in the manuscript?	Not Applicable	
If a study is subject to dual use research of concern regulations, is the name of the **authority granting approval and reference number** for the regulatory approval provided in the manuscript?	Not Applicable	

Reporting

The MDAR framework recommends adoption of discipline-specific guidelines, established and endorsed through community initiatives. Journals have their own policy about requiring specific guidelines and recommendations to complement MDAR.

Adherence to community standards	Information included in the manuscript?	In which section is the information available? (Reagents and Tools Table, Materials and Methods, Figures, Data Availability Section)
State if relevant guidelines or checklists (e.g., **ICMJE, MIBBI, ARRIVE, PRISMA**) have been followed or provided.	Not Applicable	
For **tumor marker prognostic studies,** we recommend that you follow the **REMARK** reporting guidelines (see link list at top right). See author guidelines, under 'Reporting Guidelines'. Please confirm you have followed these guidelines.	Not Applicable	
For **phase II and III randomized controlled trials**, please refer to the **CONSORT** flow diagram (see link list at top right) and submit the CONSORT checklist (see link list at top right) with your submission. See author guidelines, under 'Reporting Guidelines'. Please confirm you have submitted this list.	Not Applicable	

Data Availability

Data availability	Information included in the manuscript?	In which section is the information available? (Reagents and Tools Table, Materials and Methods, Figures, Data Availability Section)
Have **primary datasets** been deposited according to the journal's guidelines (see 'Data Deposition' section) and the respective accession numbers provided in the Data Availability Section?	Not Applicable	
Were **human clinical and genomic datasets** deposited in a public access-controlled repository in accordance to ethical obligations to the patients and to the applicable consent agreement?	Not Applicable	
Are **computational models** that are central and integral to a study available without restrictions in a machine-readable form? Were the relevant accession numbers or links provided?	Not Applicable	
If publicly available data were reused, provide the respective **data citations in the reference list**.	Not Applicable	
